# Identification of key genes in hepatitis B associated hepatocellular carcinoma based on WGCNA

**DOI:** 10.1186/s13027-021-00357-4

**Published:** 2021-03-16

**Authors:** Chang Liu, Qinghai Dai, Qian Ding, Min Wei, Xiaohong Kong

**Affiliations:** 1 School of Medicine, Nankai University, Tianjin, China; 2 Nankai University Second People’s Hospital, Nankai University, Tianjin, China

**Keywords:** HBV, HCC, WGCNA, Mitosis, Cell cycle, K-M survival

## Abstract

Chronic Infection of Hepatitis B virus (HBV) is one risk factor of hepatocellular carcinoma (HCC). Much effort has been made to research the process of HBV-associated HCC, but its molecular mechanisms of carcinogenesis remain vague. Here, weighted gene co-expression network analysis (WGCNA) was employed to explore the co-expressed modules and hub/key genes correlated to HBV-associated HCC. We found that genes of the most significant module related to HBV-associated HCC were enriched in DNA replication, p53 signaling pathway, cell cycle, and HTLV-1 infection associated pathway; these cellular pathways played critical roles in the initiation and development of HCC or viral infections. Furthermore, seven hub/key genes were identified based on the topological network analysis, and their roles in HCC were verified by expression and Kaplan-Meier survival analysis. Protein-protein interaction and KEGG pathway analysis suggested that these key genes may stimulate cellular proliferation to promote the HCC progression. This study provides new perspectives to the knowledge of the key pathways and genes in the carcinogenesis process of HBV-associated HCC, and our findings provided potential therapeutic targets and clues of the carcinogenesis of HBV-associated HCC.

**Supplementary Information:**

The online version contains supplementary material available at 10.1186/s13027-021-00357-4.

## Introduction

Hepatocellular carcinoma (HCC), which is also known as primary liver cancer, is one of the common malignant tumors, and the 2nd leading cause of cancer-related death worldwide [1, 2]. The risk factors of HCC are chronic infections of hepatitis viruses, diet polluted with aflatoxin, obesity, type two diabetes, use and abuse of alcohol and tobacco [3–5]. Among the hepatitis viruses associated with HCC, hepatitis B virus (HBV) is responsible for about 80% of virus-related HCC cases, particularly in East Asia and Africa [6]. HBV causes acute and chronic liver infections, which can then lead to liver cirrhosis and HCC. The carcinogenesis of HBV-associated HCC is a complex process, which can be summarized into the following stages: the host inflammatory reaction against HBV, interaction with endogenous mutagens, integration of viral genome DNA into cellular DNA genome, and alternating gene expression in multiple ways [7–9]. Studies of the carcinogenesis molecular process and comparisons of the difference of genetic expression between HBV-associated HCC tissues and adjacent normal tissues, will help us find interesting and important clues of the carcinogenesis, and may provide potential therapeutic targets of HBV-associated HCC. Up to date genomic technologies such as gene-array or next-generation sequence facilitate the detection of the whole genome expression.

High-throughput hybridization array- and sequencing-based experiments generate vast amount of data on the molecular abundance of RNAs (mRNAs, miRNAs, lncRNAs, cirRNAs), genomic DNAs, and proteins in absolute or relative terms. Public databases such as GEO and ArrayExpress store these data and provide information for the research community to reuse [10–12], and many data analysis tools and methods have been developed to reanalyze or reuse these data in order to get some new interesting results. Among these methods, weighted gene co-expression network analysis (WGCNA) is a powerful systems biological method to find co-expressed modules and hub/key genes in transcriptomics, proteomic and metabolomic studies [13–15]. Gene co-expression network analysis enables us to systematically analyze large, high-dimensional data sets. WGCNA groups genes into a module/network according to pairwise correlations between genes of their similar expression profile; furthermore, these models may correlate to some special clinical traits of interest, such as tumor stages, ages, gender and other biological characteristics or traits that we are interested in.

The objective of the present study is to explore novel genes or pathways relating HBV-related HCC through the data mining of public databases. In this study, we reanalyzed a GEO data set (GSE121248) of the HBV induced HCC and adjacent normal tissues, and then constructed a gene co-expression network based on WGCNA and then identified 21 modules based on the gene expression data sets. According to the results of WGCNA, there were six modules significantly correlated to the tumor trait in our study; especially the turquoise module, which is distinguished in WGCNA, is the most significant module correlated to the tumor trait. The co-expression network of these genes in the turquoise module was analyzed by Cytoscape network topological analysis tool “cytoHubba” plugin to get the hub or key genes in the network. Finally, seven hub/key genes were found correlating to HBV-associated HCC tumor trait; based on TCGA database tools, these key genes’ expression levels showed significant differences between people without HCC and HCC patients, and the expression levels of these hub/key genes also influence the survival of HCC patients significantly. With the help of WGCNA and net topological algorithms, several new key genes correlating to HBV associated HCC were found, while these genes have not been paid close attention to in the original paper that the GEO data set came from; we also predicted the functions of these genes and hope to provide some useful information to interested researchers. These genes or other cellular factors associated to these key genes could be the biomarkers and potential therapeutic targets of HBV-associated HCC.

## Material and methods

### Data processing

The gene expression data set GSE121248 (https://www.ncbi.nlm.nih.gov/geo/query/acc.cgi?acc=GSE121248), which is provided by Hui KM [16], was downloaded from the Gene Expression Omnibus (GEO) database [17]. Briefly, 107 tissue samples included 70 chronic hepatitis B induced HCC and 37 adjacent normal tissues. All the tissues were obtained from patients who underwent partial hepatectomy as a treatment for HCC. To assess recurrence, all treated HCC patients were monitored by routine clinical follow up once every 3 months. The detailed information of the study population was provided in supplementary files of Hui KM’s original article [16] and our supplementary files. The RNAs were extracted from all tissue samples, and the reversed transcription DNAs were hybridized on the human U133 plus 2.0 arrays (Affymetrix, Santa Clara, CA, USA). Affyand-related R packages were used to process all the raw data. Robust Multi-array Average approach was used to normalize the background of raw data according to the package instruction. The expression set was processed through the *nsFilter* function to filter features exhibiting little variation or a consistently low signal across samples [18].

### Weighted gene co-expression network analysis (WGCNA)

The freely accessible R package WGCNA (v 1.66) was taken to co-expression analysis [15]. According to the instruction, one-step network construction and module detection was taken; 15,139 annotated genes were used to construct the network. According to the software instruction, the module eigengene expression, adjacency matrix heatmap, Module-Trait relationships, and other related parameters/results were calculated and visualized. Briefly, the network type is chosen as an unsigned network, and the correlation method is Pearson correlation.

### GO term and KEGG pathway enrichment analysis

Based on Bioconductor packages “clusterProfiler”, GO term enrichment analysis including biological process, cellular component and molecular function, was used to explore the biologic significance of selected module genes. With the same Bioconductor packages “clusterProfiler”, we also performed the KEGG pathway enrichment analysis of the selected module genes [19].

### Key genes identification of the selected module

To identify hub/key genes of the selected module, the Cytoscape software (3.7.1) was utilized to construct of the network of the module genes. The important nodes (key genes) were predicted and explored by “cytoHubba” plugin [20]. The topological algorithms of “cytoHubba” consist of Degree, Edge Percolated Component (EPC), Maximum Neighborhood Component (MNC), Density of Maximum Neighborhood Component (DMNC), Maximal Clique Centrality (MCC) and centralities based on shortest paths, such as Bottleneck (BN), EcCentricity, Closeness, Radiality, Betweenness, and Stress, were applied to get respective top 20 ranked genes set. Then, the intersection of nine top 20 ranked genes sets were identified as the key genes.

### Expression on box plots

The website GEPIA (http://gepia.cancer-pku.cn/index.html) was taken to box plot the expression of the key genes [21]. According to the web tutorial, the liver hepatocellular carcinoma (LIHC) dataset was chosen; log_2_(TPM + 1) was used as log scale; jitter size was 0.4; the normal data for control was match GTEx data and TCGA normal.

### Kaplan-Meier survival analysis

KM-plotter (http://kmplot.com/analysis/) was employed to perform the survival analysis of the combination and respective key genes [22]. Briefly, Liver Cancer RNA-seq data set was selected; patient groups were split by median expression of the gene (auto select best cutoff); overall survival was applied.

### Prediction of the functions of key genes

The GeneMANIA Cytoscape plugin was used to predict the functions of key genes [23, 24]. The identified key genes were inputted as a query gene set. Based on *Homo sapiens* database of GeneMANIA, a network of query genes and result genes was constructed and visualized by Cytoscape. The distinctive relationships between the genes, including co-expression, co-localization, genetic interaction, pathway, physical interaction, shared protein domains, and predicted relations, were indicated by distinct color lines. In order to show the KEGG pathways of the key/hub genes, an R/Bioconductor package named Pathview was applied to visualize the concerned KEGG pathways [25].

## Results

### Data processing

**Fig. 1:**
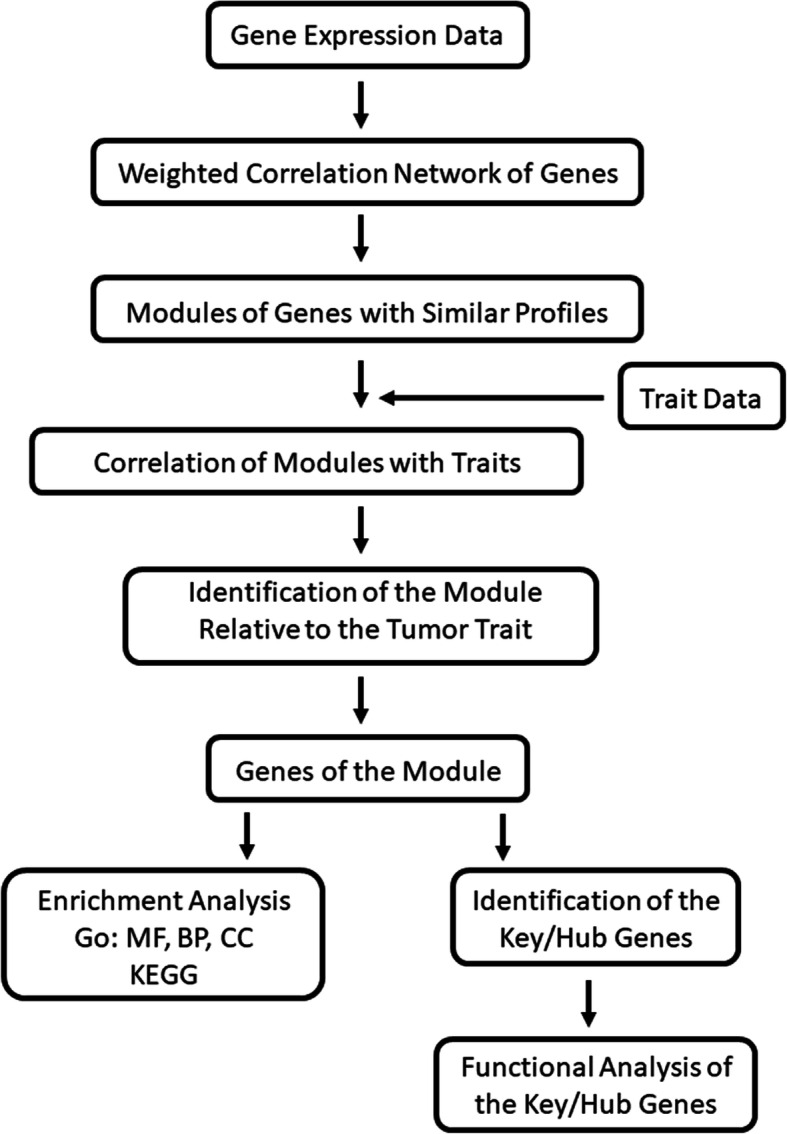
Flowchart of the Study. Abbreviation: GO, Gene Ontology; MF, Molecular Function; BP, Biological Process; CC, Cellular Component; KEGG, Kyoto Encyclopedia of Genes and Genomes

GSE121248 raw files (.CEL format), which contains a total of 107 tissue sample were downloaded from the NCBI website (ftp://ftp.ncbi.nlm.nih.gov/geo/series/GSE121nnn/GSE121248/). The files were then transferred to expression matrix using the RMA algorithm based on R language, including background correction, normalization and summarization. (Supplementary Figure 1: (A) Box plot of relative log expression (RLE) and (B) Box plot of normalized unscaled standard errors (NUSE)). After annotation and *nsFilter* processing, there were 15,139 genes from all 54,675 probes for further WGCNA analysis. In order to give an outline of our study design, a workflow is shown in Fig. 1.

### Weighted gene co-expression network identification of modules construction

**Fig. 2:**
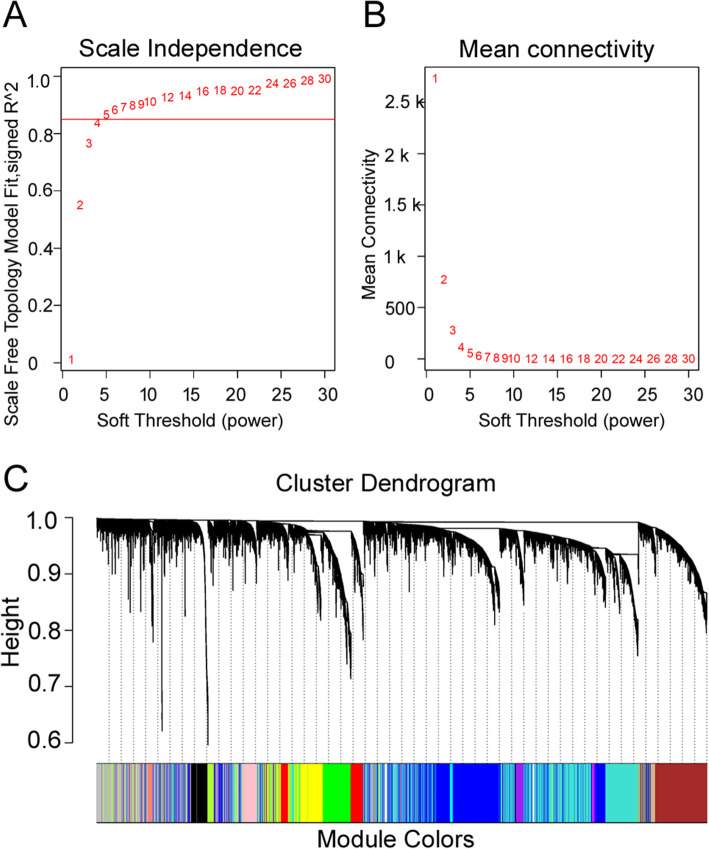
Construction of Weighted Gene Co-Expression Network Identification of Modules. **a** Scale independence of network topology for different soft-thresholding powers. The approximate scale-free topology can be attained at the soft-thresholding power of 5. **b** Mean connectivity of network topology for different soft-thresholding powers. Numbers in the plots indicate the corresponding soft thresholding powers. **c** DEGs clustering and module screening based on gene expression pattern. The top was gene dendrogram and the bottom was genes’ modules with different colors. A total of 21 modules were identified

The co-expression network was constructed from the filtered annotated genes, in which 21 modules were identified. Before the net construction, the samples were clustered to see if there are any obvious outliers, and it appears that there was no outlier (see supplementary Figure 2). As shown in Fig. 2(a) and (b), the soft threshold power 5 was chosen to define the adjacency matrix based on the criterion of approximate scale-free topology, and the minimum module size was 21. The modules with different colors were shown in Fig. 2(c); the module grey (MEgrey) is reserved for genes outside of all modules. To show the co-expression relationship between the genes on genome level, 400 genes were randomly selected to plot the network heatmap as shown in supplementary Figure 3.

### Correlation between each module and clinical traits

**Fig. 3:**
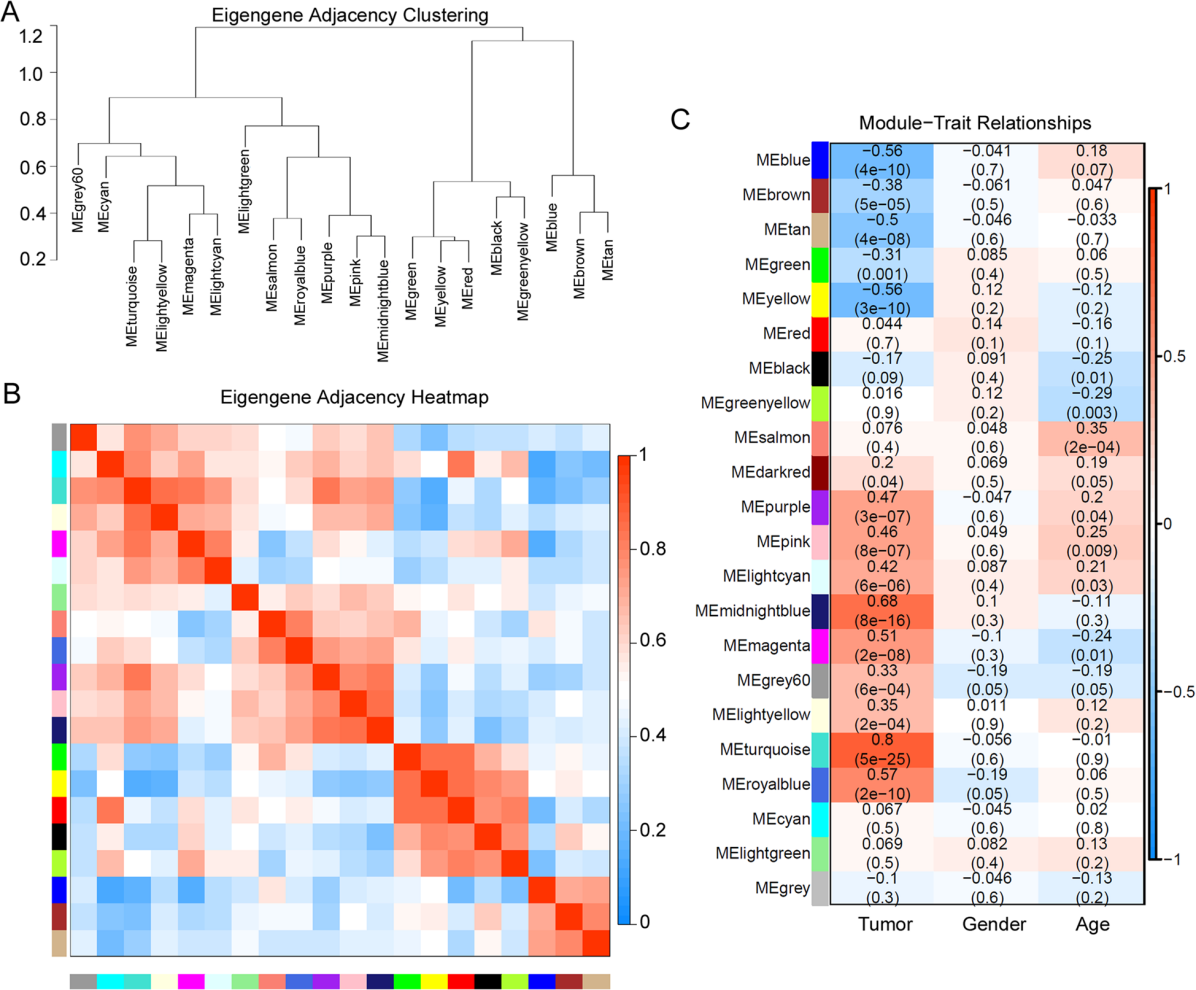
Gene Modules Identified by Weighted Gene Co-Expression Network Analysis. **a** Dendrogram of consensus module eigengenes obtained by WGCNA on the consensus correlation. **b** Heatmap plot of the adjacencies of modules. Red represents positive correlation and blue represents negative correlation. **c** Relationships of consensus module eignegenes and clinical traits. The module name is shown on the left side of each cell. Numbers in the table report the correlations of the corresponding module eigengenes and traits, with the *p* values printed below the correlations in parentheses. The table is color coded by correlation according to the color legend. Intensity and direction of correlations are indicated on the right side of the heatmap (red, positively correlated; blue, negatively correlated)

To figure out the interactions among these 20 co-expressed modules (except MEgrey, which is reserved for genes outside of all modules by WGCNA), we analyzed the connectivity of eigengenes. As shown in Fig. 3(a), a cluster analysis was performed; 20 modules were classified into two clusters, and each cluster contains two main branches. Figure 3(a) and (c) showed a significant difference among the 21 modules. There are multiple modules related to the clinical trait between tumor samples and adjacent normal samples. The module-trait heatmap represents the correlations of the module eigengenes with traits. When that correlation is positive, it means the eigengene increases with increasing trait. Generally, in a signed network, where all genes in a module are positively correlated with the eigengene, it means that all genes in the module should follow the same pattern of increasing expression with increasing trait values; on the contrary, in an unsigned network, which is actually the case in our study, there are also some genes that have the opposite behavior compared with the eigengene. It means that we do not know for sure if the expression of genes in the modules actually increases or decreases, but we know the expression definitely changes. As shown in Fig. 3, the midnight blue, magenta, turquoise, royal blue modules were positively related to the tumor trait, especially the turquoise module (MEturquoise) was the most significantly relative module to the tumor trait (correlations 0.8, *p* value 5 × 10^− 25^). On the other hand, the blue, tan, yellow modules were negatively related to the tumor trait. There was no module significantly related to the gender trait, and it is releasable. Interestingly, the salmon module (MEsalmon) was slightly related to the age trait.

### Functional enrichment and pathway analysis of the key module genes

**Fig. 4:**
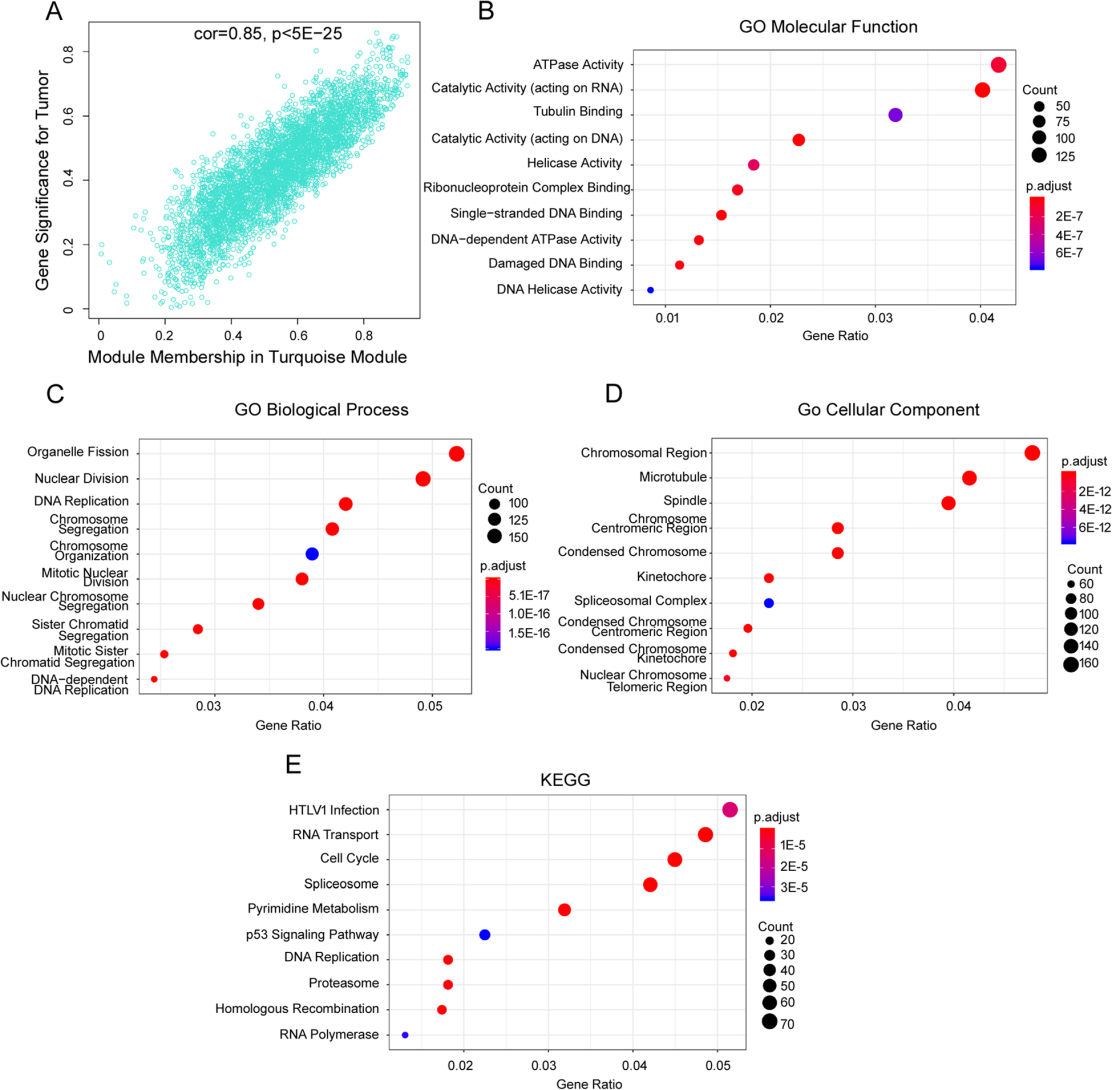
Functional Enrichment and Pathway Analysis of the Key Module Genes. **a** Relationship between module membership in turquoise module and gene significance for tumor. **b** GO MF molecular function enrichment analysis result of turquoise module genes. **c** GO BP biological process enrichment analysis result of turquoise module genes. **d** GO CC cellular component enrichment analysis result of turquoise module genes. **e** KEGG enrichment analysis results of turquoise module genes. Pathway names are shown on the left. The size of the round represented the number of genes enriched in the corresponding pathway. The color of the round represented the adjusted *p* value

Because MEturquoise is the most relevant module related to the tumor trait, we did the further functional assay of the genes in turquoise module. As shown in Fig. 4(a), the higher module membership genes in MEturquoise are the more significant genes for the tumor trait; they have a strong positive correlated relationship. Then, we did GO term enrichment analysis of MEturquoise genes. As to molecular functions in Fig. 4(b), the MEturquoise genes considerably enriched in ATPase activity, catalytic activity (acting on RNA or DNA), helicase activity, macromolecule binding (such as tubulin, ribonucleoprotein complex, single-stranded DNA, damaged DNA). For biological process as shown in Fig. 4(c), the MEturquoise genes significantly enriched in organelle fission, nuclear division, chromosome functions (such as segregation, organization). With regard to cellular component in Fig. 4(d), the MEturquoise genes significantly enriched in a chromosomal and microtubule region, including normal or condensed chromosome, centromere, kinetochore, telomere. In order to get the pathway involved MEturquoise genes, KEGG pathway analysis was taken. As shown in Fig. 4(e), several significant enriched pathways were found, including HTLV1 infection, RNA transport, cell cycle, spliceosome, pyrimidine metabolism, p53 signaling pathway, DNA replication and so on.

### Network analysis of MEturquoise genes

**Fig. 5:**
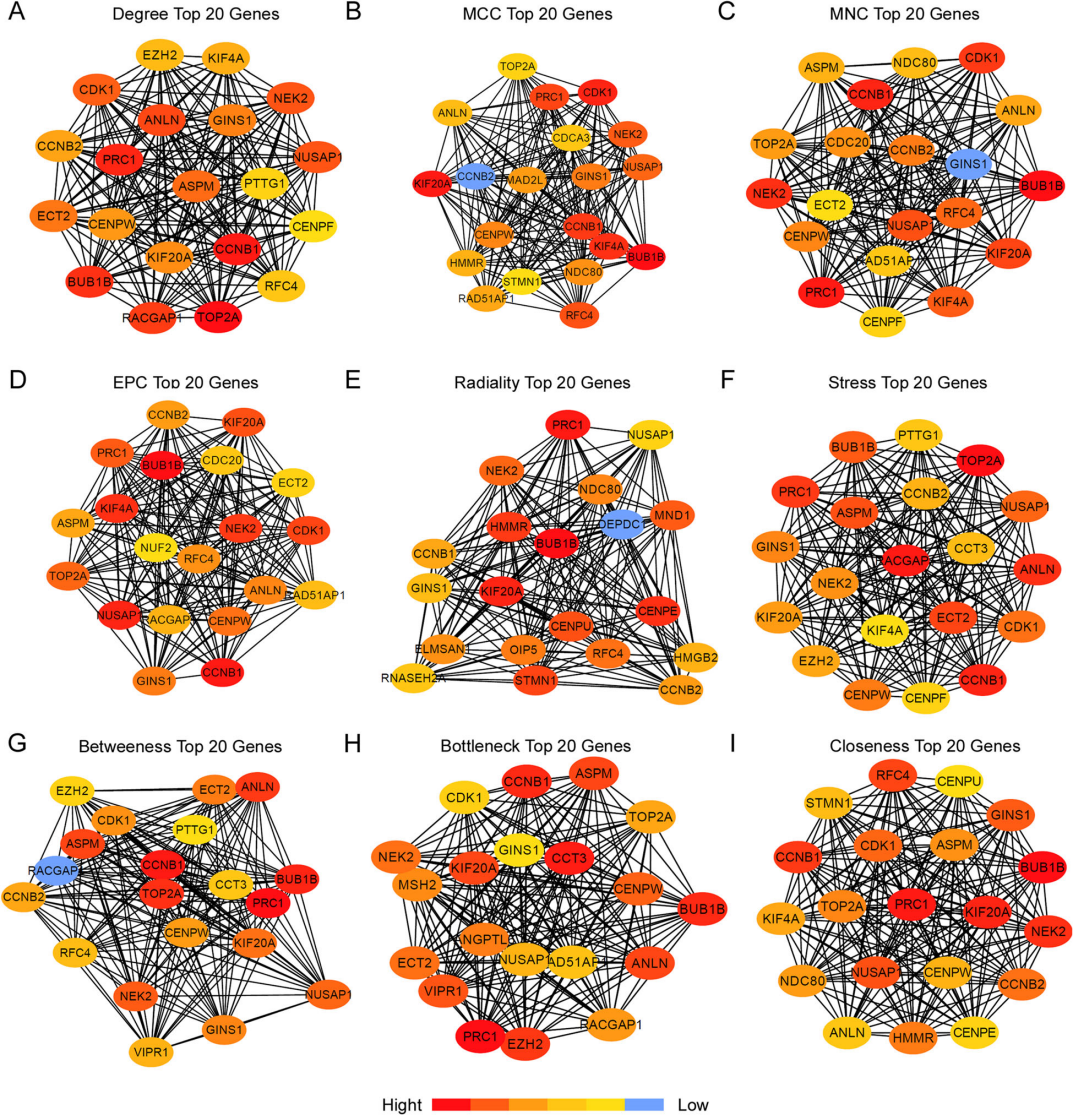
The Network of Top Ranked Genes through Different Topological Algorithms. **a** Degree top 20 genes network. **b** MCC top 20 genes network. **c** MNC top 20 genes network. **d** EPC top 20 genes network. **e** Radiality top 20 genes network. **f** Stress top 20 genes network. **g** Betweenness top 20 genes network. **h** Bottleneck top 20 genes network. **i** Closeness top 20 genes network. Different colors represented distinct ranks, and lines between the genes showed co-expression relationship

In order to identify the hub genes or key genes of the module turquoise, the MEturquoise network file was imported into the Cytoscape. The module net, which has 3,600,506 edges, is too huge to be analyzed on a personal computer. The net was firstly cut off by edge weight (more than 0.1) into 92,188 edges, and then the cutoff net was analyzed by “cytoHubba” Cytoscape plugin. There are 12 topologic algorithms available in cytoHubba plugin. The top ranking 20 genes’ sets of each topologic algorithm were all obtained, and then the intersections of all the sets were taken. There were three sets getting none or too few genes with other sets, so they were discarded. The sub networks of the top 20 genes of other nine topological algorithms were shown in Fig. 5. The intersections of all nine genes’ sets were these seven genes: *CCNB1, GINS1, PRC1, KIF20A, NUSAP1, NEK2, BUB1B*. These seven genes were considered as hub genes or key genes involved in HBV associated HCC in our study, and Table 1 showed the detail information of these genes. The annotation of these 7 key genes was given by GEPIA website [21].

**Table 1:** Key Genes Involved in HBV Associated HCC

Gene	Ensembl ID	Description	Alias	Summary
CCNB1	ENSG00000134057.14	cyclin B1	CCNB	The protein encoded by this gene is a regulatory protein involved in mitosis. The gene product complexes with p34(cdc2) to form the maturation-promoting factor (MPF). Two alternative transcripts have been found, a constitutively expressed transcript and a cell cycle-regulated transcript, that is expressed predominantly during G2/M phase. The different transcripts result from the use of alternate transcription initiation sites.
GINS1	ENSG00000101003.9	GINS complex subunit 1	PSF1	The yeast heterotetrameric GINS complex is made up of Sld5 (GINS4; MIM 610611), Psf1, Psf2 (GINS2; MIM 610609), and Psf3 (GINS3; MIM 610610). The formation of the GINS complex is essential for the initiation of DNA replication in yeast and Xenopus egg extracts.
PRC1	ENSG00000198901.13	protein regulator of cytokinesis 1	ASE1	This gene encodes a protein that is involved in cytokinesis. The protein is present at high levels during the S and G2/M phases of mitosis but its levels drop dramatically when the cell exits mitosis and enters mitosis G1 phase. It is located in the nucleus during interphase, becomes associated with mitotic spindles in a highly dynamic manner during mitosis, and localizes to the cell mid-body during cytokinesis. This protein has been shown to be a substrate of several cyclin-dependent kinases (CDKs). It is necessary for polarizing parallel microtubules and concentrating the factors responsible for contractile ring assembly. Alternative splicing results in multiple transcript variants.
KIF20A	ENSG00000112984.11	kinesin family member 20A	MKLP2, RAB6KIFL	–
NUSAP1	ENSG00000137804.12	nucleolar and spindle associated protein 1	ANKT, BM037, LNP, NUSAP, PRO0310p1, Q0310, SAPL	NUSAP1 is a nucleolar-spindle-associated protein that plays a role in spindle microtubule organization.
NEK2	ENSG00000117650.12	–	–	–
BUB1B	ENSG00000156970.12	BUB1 mitotic checkpoint serine/threonine kinase B	BUB1beta, BUBR1, Bub1A, MAD3L, MVA1, SSK1, hBUBR1	This gene encodes a kinase involved in spindle checkpoint function. The protein has been localized to the kinetochore and plays a role in the inhibition of the anaphase-promoting complex/cyclosome (APC/C), delaying the onset of anaphase and ensuring proper chromosome segregation. Impaired spindle checkpoint function has been found in many forms of cancer.

### Roles of the key genes in the process of HHC

**Fig. 6:**
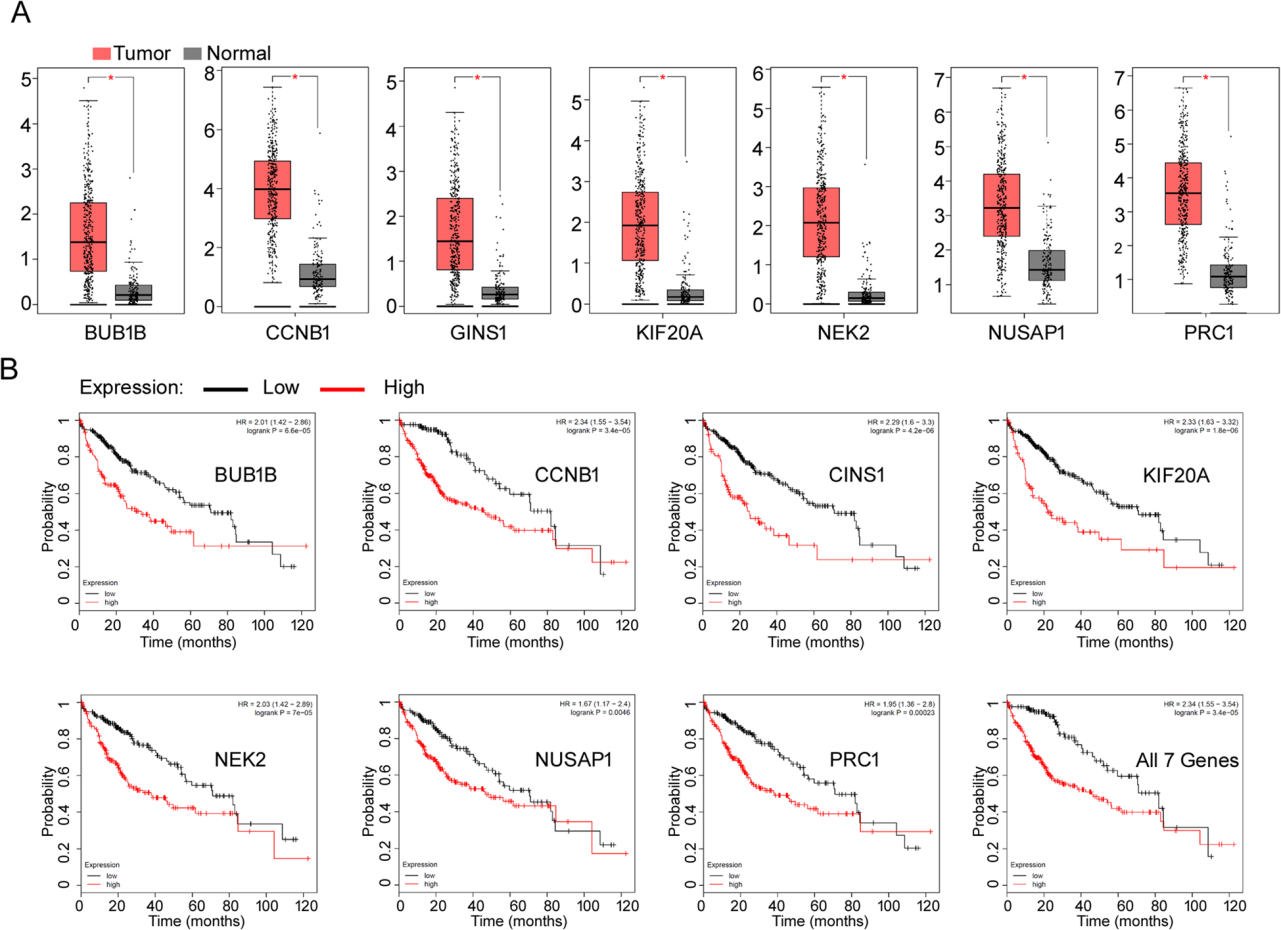
Roles of the Key Genes in the Process of HBV Associated HCC. **a** Boxplot of key genes’ expression between HCC tissues and normal tissues. “*” indicated *p* value is less than 0.05. **b** Kaplan-Meier curves of key genes in HCC patients. Red and black curves represent High- and Low-risk groups, respectively

To analyze the functions of key genes, publicly available data and tools from TCGA and GTEx databases were applied to analyze whether these expression levels of 7 key genes were substantially different between healthy people and HCC patients and whether the expression levels of 7 key genes may influence the survival of HCC patient. As showing in Fig. 6(a), the expression levels of all seven key genes in 369 HCC tumor tissues are significantly higher than the respective gene expression level in 160 normal tissues. As shown in Fig. 6(b), the patients who have higher levels of expression of the key genes show shorter overall survival periods compared with lower expression patients.

### Functional prediction of the key genes involved in HBV associated HCC

**Fig. 7:**
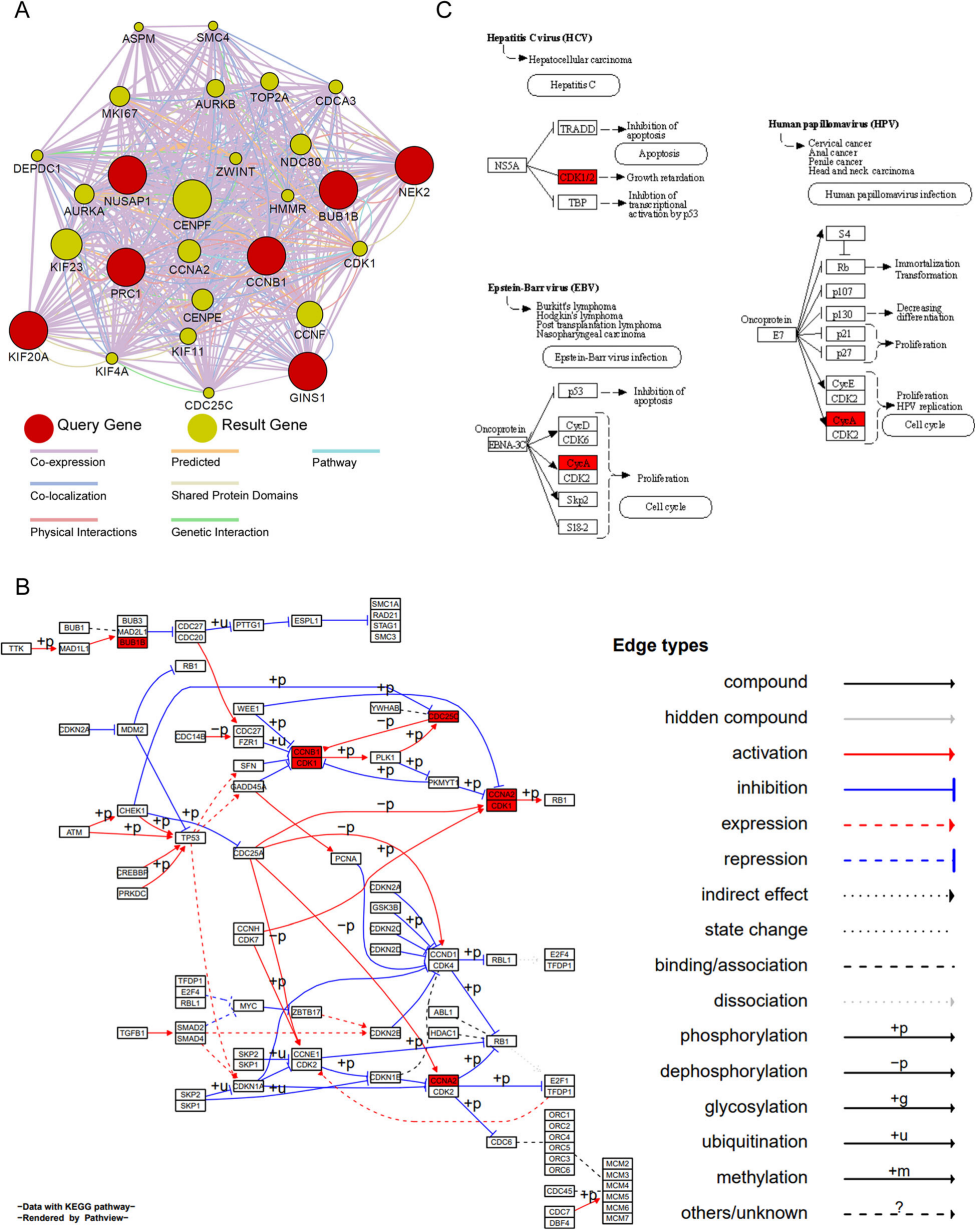
Functional Prediction of the Key Genes Involved in HBV Associated HCC. **a** Protein-Protein-Interaction (PPI) Network of the Key Genes Involved in HBV Associated HCC. GeneMANIA was applied to predict the function of key genes and construct the PPI network. The red rounds indicated key genes (query genes), and the khaki rounds indicated result genes. The different color lines between the rounds indicated distinct relationships between corresponding genes/proteins, such as co-expression, co-localization, physical interactions, predicted relations, shared protein domains, genetic interactions and pathway. **b** Visualization of the CELL CYCLE (hsa04110) KEGG pathway. The genes in the PPI network are highlighted in red. The different relations between proteins in the pathway are indicated in distinct types of lines. **c** The VIRAL CARCINOGENESIS pathways (hsa05203) related to the genes in PPI network. The genes in the PPI network are also highlighted in red

To predicting the functions of 7 key genes, GeneMANIA plugin (version 3.5.0) was applied to find the result genes and construct the PPI net. As showing in Fig. 7(a), 20 result genes were found to relate to the key genes/query genes; they are *CENPF, KIF23, CCNF, CCNA2, CENPE, NDC80, MKI67, AURKA, TOP2A, AURKB, KIF11, CDK1, CDCA3, HMMR, ZWINT, KIF4A, DEPDC1, CDC25C, SMC4, ASPM*. Most of the result genes are centromere proteins or involved in cell cycle processing. GeneMANIA predicts seven different relationships between genes/proteins based on published papers, including co-expression, physical interactions, co-localization, predicted relations, shared protein domains, genetic interactions and pathway. These relationships were indicted as distinct colors as shown in Fig. 7(a). From the Fig. 7(a), the co-expression is the main relationships among key genes and result genes. The result is intelligible, because all the key genes are come from the same co-expression module; besides the co-expression, there are other important relations predicted for us, such as co-localization, physical interactions, predicted, shared protein domains, genetic interactions; you can get more detailed relations in the supplementary file (supplementary Tables 1, 2, 3). Interestingly, the predicted relation was based on Stein’s work about protein interaction network on cancer data analysis, so it indicated these genes played critical roles in a carcinogenesis process [26].

### Key pathway analysis of the genes involved in HBV associated HCC

Based on the results of key genes’ PPI net as shown in Fig. 7(a), seven key/hub genes and 20 results genes were used to search the key KEGG pathways; as the result of Fig. 7(b), the CELL CYCLE (hsa04110) pathway was regarded as the key pathway in our study. The genes *BUB1B, CCNB1, CDK1, CDC25C* and *CCNA2*, which were also in PPI net, take essential places in the CELL CYCLE (hsa04110) pathway. This result implicated that HBV might influence the cell cycle pathway through those above-mentioned genes, and eventually effect the process of HCC. Two genes in PPI net, namely *CDK1* and *CycA* (*CCNA2*), were also found in the VIRAL CARCINOGENESIS (hsa05203) KEGG pathways. As we can see in Fig. 7(c), non-structural proteins of HCV, EBV or HPV can stimulate the cellular proliferation through two above genes. The genes in our PPI net were not found in the VIRAL CARCINOGENESIS pathway of HBV, but our results implicated that HBV might promote proliferation of HCC tumor cells in a similar way.

## Discussion

In our studies, WGCNA was employed to explore gene expression alternation between HBV associated HCC tissues and adjacent normal tissues. There are many advantages of WGCNA over other traditional methods for differential expression analysis, because it focuses on co-expression patterns and the functional relevant modules that consist of related genes will be discovered. Key/hub genes in the modules related to some specific traits may serve as clinical detective biomarkers or therapeutic targets [27–29].

HCC is a common consequence of HBV chronic infection. HBV‘s prolonged infection can change the expression of genes of hepatic cells in different ways. HBV-DNA integrations randomly distributed among chromosomes into host chromosomes, and the integrations of HBV will alternate the expression of cellular genes near the integrating sites [30]. Noncoding RNAs, such as miRNAs, lncRNAs and circRNAs, also take parts in the pathogenesis of HBV-associated HCC [31–33]. Viral protein HBx plays important roles in hepatocarcinogenesis by interfering with telomerase activity [34], affecting hepatocellular apoptosis [35, 36], and up-regulating the transcriptional activation of human telomerase transcriptase [37]. Interesting, our GO cellular component enrichment results of genes of module turquoise, which is the co-expression module most positively related to the tumor trait, showed that some genes of this module were enriched in the telomeric region.

In our study, modules changed significantly between HBV associated HCC tissues and normal adjacent tissues consisted of the midnight blue, magenta, turquoise, royal blue modules which were up-regulated in HCC, whereas the blue, tan, yellow modules which were down-regulated in HCC. These up-regulated modules and down-regulated modules mentioned above were classified into two main groups through eigengene adjacency clustering as shown in Fig. 2(a). Among these modules, the turquoise module is the most significant module related to the tumor trait. According to the KEGG results, the turquoise module, enriched in DNA replication, p53 signaling pathway, cell cycle, especially HTLV-1 infection associated pathway, increased in HBV-associated HCC tissues. These important processes, including the acceleration of genome DNA replication, the misregulation of tumor suppressor p53, and the abnormal cell cycle-associated pathway, all played critical roles in the initiation and development of HCC. Furthermore, the enrichment of HTLV-1 infection associated pathway indicated that some signaling pathways associated with viral infections were also significantly activated in HBV-associated HCC tissues.

According to the topological network analysis from the turquoise model, seven key genes were identified playing critical roles in the network. They are *CCNB1, GINS1, PRC1, KIF20A, NUSAP1, NEK2,* and *BUB1B*. *CCNB1* is the gene, which encodes a regulatory protein involved in mitosis, and it is expressing predominantly during G2/M phase [38]. It was reported that *CDK1-CCNB1* enables MPS1 kinetochore localization to create a spindle checkpoint-permissive state [39].*GINS1* encodes subunit 1 of the GINS complex, and the complex is essential for the initiation of DNA replication [40]. It has been reported that the high expression of *KIF20A* is associated with poor prognosis of glioma patients, and *KIF20A* can be a potential immunotherapeutic target for glioma [41, 42]. *PRC1* encodes a protein involved in the cytokinesis process, and the protein maintains a high level during the S and G2/M phases of mitosis [43]. *NUSAP1* encodes a nucleolar-spindle-associated protein playing a role in spindle microtubule organization [44]. *NEK2* has shown it is involved in some different cancers: *NEK2* promotes aerobic glycolysis in multiple myeloma [45]; targeting *NEK2* attenuates glioblastoma growth and radioresistance [46]; *NEK2* can be a prognostic biomarker of hepatocellular carcinoma [47]. *BUB1B* encodes a kinase playing a role in spindle checkpoint function. The kinase localizes to the kinetochore and plays a role in the inhibition of the anaphase-promoting complex/cyclosome (APC/C) [48]. It has been reported that individuals having biallelic *TRIP13* or *BUB1B* mutations are prone to having embryonal tumors, and their cells display severe spindle assembly checkpoint (SAC) impairment [49]. In our expression and survival analyses, all 7 key/hub genes, which were identified in our selected module, can be the biomarkers and potential therapeutic targets of HBV-associated HCC.

According to genes’/proteins’ multifunctional relations, we constructed a protein-protein interaction net consisting of 27 genes (our seven key/hub genes as query genes, and 20 result genes). For total 27 genes, we search KEGG for their pathway information. Finally, we focused on CELL CYCLE (hsa04110) pathway and VIRAL CARCINOGENESIS (hsa05203) KEGG pathways. Genes *BUB1B, CCNB1, CDK1, CDC25C* and *CCNA2* play important roles in cell cycle regulation; HCV, EBV and HPV can stimulate cell proliferation through *CDK1* or *CCNA2*. These genes were not found in HBV carcinogenesis pathways, but our results implied that there should be an unknown regulation of cell cycle in HBV-related HCC. Specially, the function of *BUB1B* and *CCNB1* in HBV-related HCC has attracted the attention of some researchers in recent years [50–52]; HBV may regulate these two genes to influence cell cycle progression promoting the development of HCC.

In summary, we provide a systematic biological interpretation of gene expression data derived from HBV associated HCC tissues and adjacent normal tissues. Based on WGCNA, there were 21 modules identified, and the turquoise module which was the most significant module relating to the tumor trait was selected to be analyzed in detail. Our results showed that the turquoise module, enriched in DNA replication, p53 signaling pathway, cell cycle, and HTLV-1 infection associated pathway, was activated in HBV-associated HCC tissues. Seven hub/key genes were identified; pathway analysis implicates that these key genes may stimulate cellular proliferation to promote the HBV-related HCC progression. All of our findings provide new perspectives to the understanding of pathways and genes underlying HBV-associated HCC, and experimental verification is needed to validate our predictions.

## Supplementary Information

**Additional file 1.**

**Additional file 2.**

## Data Availability

All data, models, or codes generated or used during the study are available from the corresponding author (Chang Liu: changliu@nankai.edu.cn) by request.
