# Supplementary material for: Identification of key genes in hepatitis B associated hepatocellular carcinoma based on WGCNA
Source: Infect Agent Cancer. 2021 Mar 16;16:18. doi: 10.1186/s13027-021-00357-4 (PMC7962393; doi:10.1186/s13027-021-00357-4)
Supplement: Supplementary file 2 — Additional file 2. [file 13027_2021_357_MOESM2_ESM.docx]

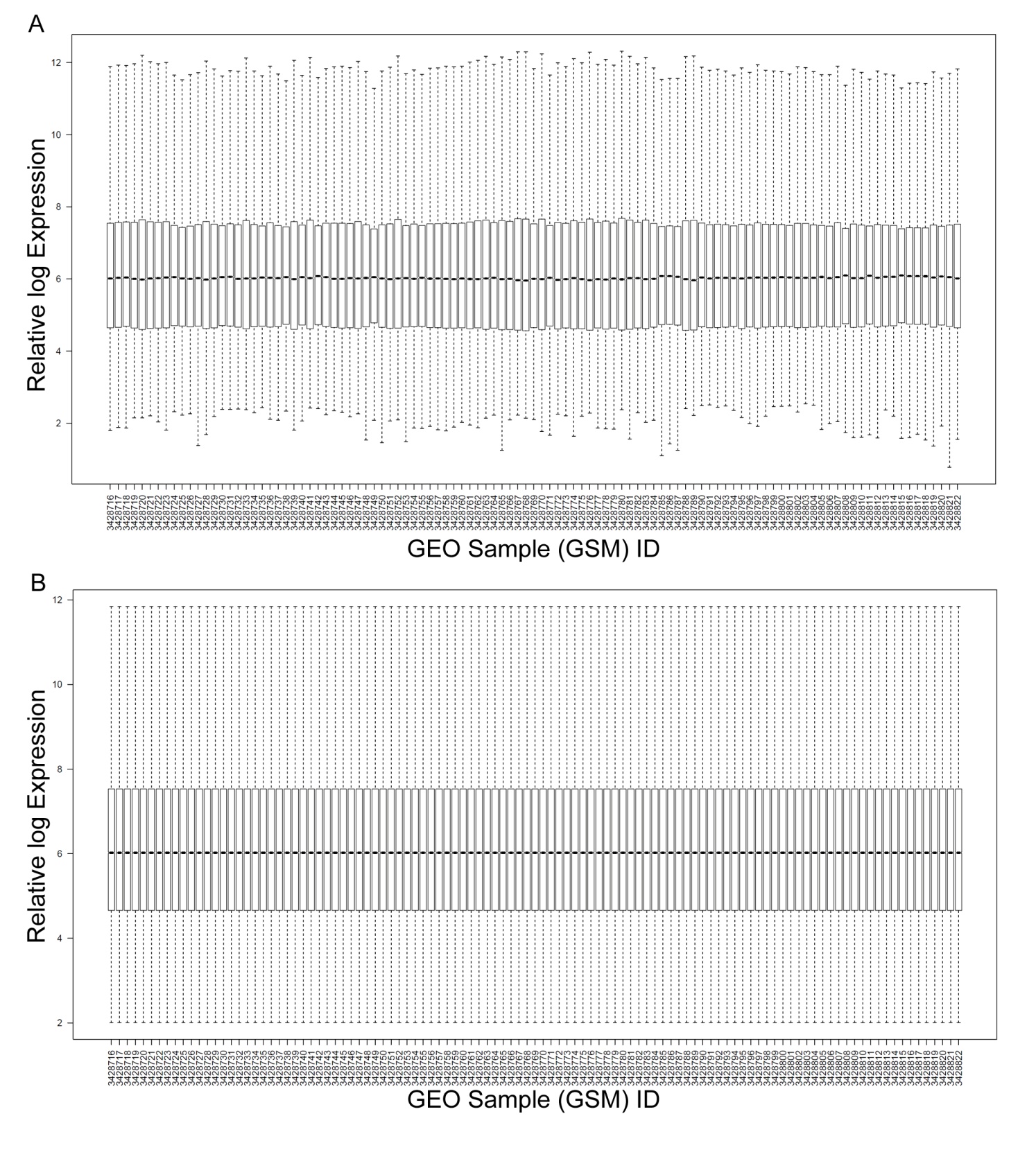


**Supplementary Figure 1.** Relative Log Expression Boxplot of GSE121248GEO Samples. (A) Relative log expression (RLE) box plot before normalization. (B) Normalized unscaled standard errors (NUSE) box plot.

**
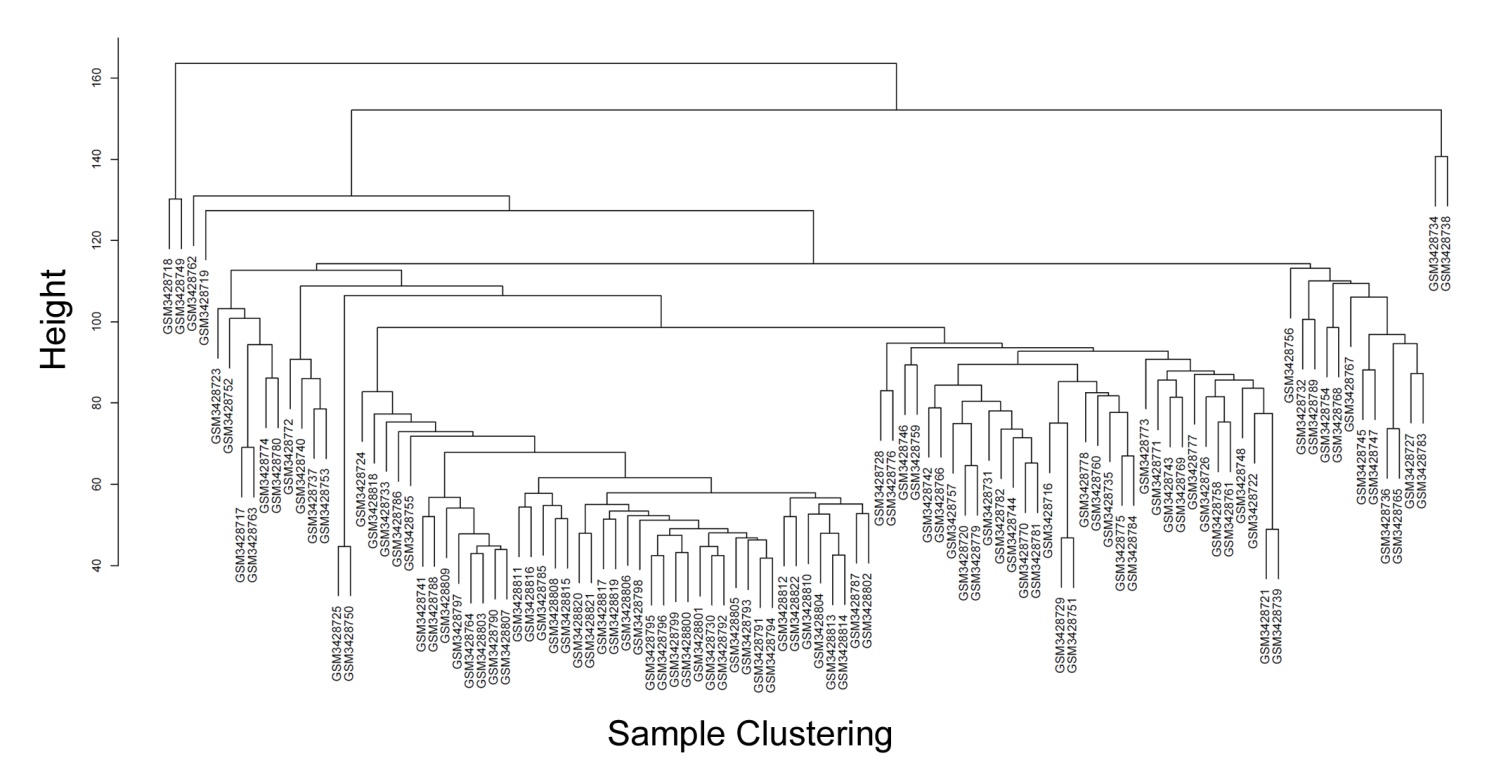
**

**Supplementary Figure 2.** Sample Clustering of GSE121248 GEO Samples.


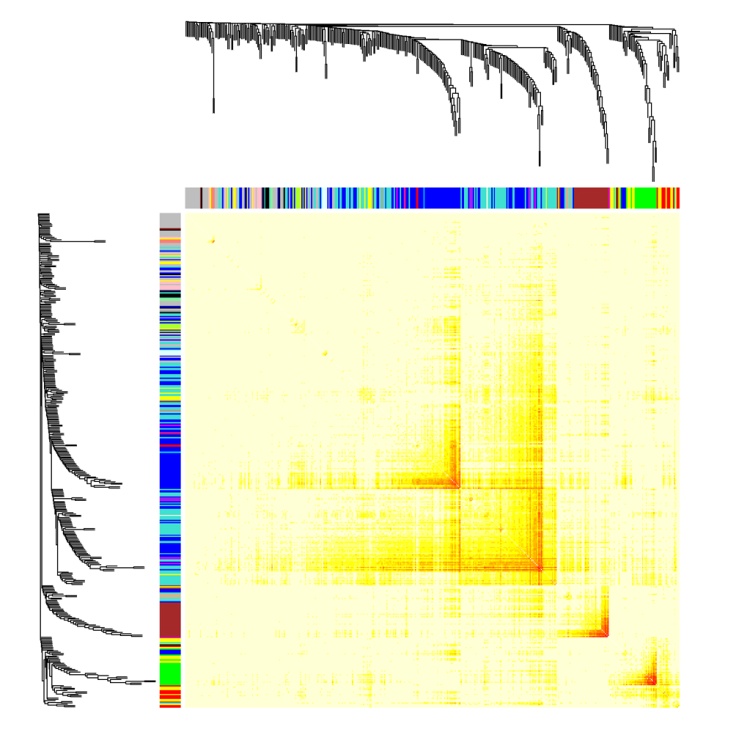


**Supplementary Figure 3.** Network heatmap of genes. The heatmap depicts the topological overlap matrix among randomly selected 400 genes in the analysis. Genes in modules with high overlap are shown in dark red.

**Supplementary Table 1.** Gene Table of GeneMANIA Results

| **Gene** | **Score** | **Description** |
| --- | --- | --- |
|  |  |  |
| NEK2 |  | NIMA related kinase 2 [Source:HGNC Symbol;Acc:HGNC:7745] |
| KIF20A |  | kinesin family member 20A [Source:HGNC Symbol;Acc:HGNC:9787] |
| NUSAP1 |  | nucleolar and spindle associated protein 1 [Source:HGNC Symbol;Acc:HGNC:18538] |
| GINS1 |  | GINS complex subunit 1 [Source:HGNC Symbol;Acc:HGNC:28980] |
| PRC1 |  | protein regulator of cytokinesis 1 [Source:HGNC Symbol;Acc:HGNC:9341] |
| BUB1B |  | BUB1 mitotic checkpoint serine/threonine kinase B [Source:HGNC Symbol;Acc:HGNC:1149] |
| CCNB1 |  | cyclin B1 [Source:HGNC Symbol;Acc:HGNC:1579] |
| CENPF | 1.52 | centromere protein F [Source:HGNC Symbol;Acc:HGNC:1857] |
| KIF23 | 1.44 | kinesin family member 23 [Source:HGNC Symbol;Acc:HGNC:6392] |
| CCNF | 1.38 | cyclin F [Source:HGNC Symbol;Acc:HGNC:1591] |
| CCNA2 | 1.35 | cyclin A2 [Source:HGNC Symbol;Acc:HGNC:1578] |
| CENPE | 1.33 | centromere protein E [Source:HGNC Symbol;Acc:HGNC:1856] |
| NDC80 | 1.32 | NDC80, kinetochore complex component [Source:HGNC Symbol;Acc:HGNC:16909] |
| MKI67 | 1.31 | marker of proliferation Ki-67 [Source:HGNC Symbol;Acc:HGNC:7107] |
| AURKA | 1.31 | aurora kinase A [Source:HGNC Symbol;Acc:HGNC:11393] |
| TOP2A | 1.29 | topoisomerase (DNA) II alpha [Source:HGNC Symbol;Acc:HGNC:11989] |
| AURKB | 1.29 | aurora kinase B [Source:HGNC Symbol;Acc:HGNC:11390] |
| KIF11 | 1.27 | kinesin family member 11 [Source:HGNC Symbol;Acc:HGNC:6388] |
| CDK1 | 1.25 | cyclin dependent kinase 1 [Source:HGNC Symbol;Acc:HGNC:1722] |
| CDCA3 | 1.24 | cell division cycle associated 3 [Source:HGNC Symbol;Acc:HGNC:14624] |
| HMMR | 1.22 | hyaluronan mediated motility receptor [Source:HGNC Symbol;Acc:HGNC:5012] |
| ZWINT | 1.22 | ZW10 interacting kinetochore protein [Source:HGNC Symbol;Acc:HGNC:13195] |
| KIF4A | 1.22 | kinesin family member 4A [Source:HGNC Symbol;Acc:HGNC:13339] |
| DEPDC1 | 1.22 | DEP domain containing 1 [Source:HGNC Symbol;Acc:HGNC:22949] |
| CDC25C | 1.21 | cell division cycle 25C [Source:HGNC Symbol;Acc:HGNC:1727] |
| SMC4 | 1.19 | structural maintenance of chromosomes 4 [Source:HGNC Symbol;Acc:HGNC:14013] |
| ASPM | 1.18 | abnormal spindle microtubule assembly [Source:HGNC Symbol;Acc:HGNC:19048] |

**Supplementary Table 2.** Net Group Table of GeneMANIA Results

| **Network Group** | **Network** | **Weight** | **Title** | **Year** | **Publication** | **PMID** | **Interactions** | **Source** |
| --- | --- | --- | --- | --- | --- | --- | --- | --- |
|  |  |  |  |  |  |  |  |  |
| **Co-expression** | | 88.07 |  |  |  |  |  |  |
|  | Innocenti-Brown-2011 | 22.11 | Identification, replication, and functional fine-mapping of expression quantitative trait loci in primary human liver tissue. | 2011 | PLoS Genet | 21637794 | 603765 | GEO |
|  | Roth-Zlotnik-2006 | 15.87 | Gene expression analyses reveal molecular relationships among 20 regions of the human CNS. | 2006 | Neurogenetics | 16572319 | 669062 | GEO |
|  | Wang-Cheung-2015 | 13.77 | Genetic variation in insulin-induced kinase signaling. | 2015 | Mol Syst Biol | 26202599 | 411047 | GEO |
|  | Smirnov-Cheung-2009 | 8.07 | Genetic analysis of radiation-induced changes in human gene expression. | 2009 | Nature | 19349959 | 461500 | GEO |
|  | Dobbin-Giordano-2005 | 5.46 | Interlaboratory comparability study of cancer gene expression analysis using oligonucleotide microarrays. | 2005 | Clin Cancer Res | 15701842 | 444931 | GEO |
|  | Ramaswamy-Golub-2001 | 5.03 | Multiclass cancer diagnosis using tumor gene expression signatures. | 2001 | Proc Natl Acad Sci U S A | 11742071 | 275113 | SUPPLEMENTARY  _MATERIAL |
|  | Noble-Diehl-2008 | 4.51 | Regional variation in gene expression in the healthy colon is dysregulated in ulcerative colitis. | 2008 | Gut | 18523026 | 661539 | GEO |
|  | Burington-Shaughnessy-2008 | 3.22 | Tumor cell gene expression changes following short-term in vivo exposure to single agent chemotherapeutics are related to survival in multiple myeloma. | 2008 | Clin Cancer Res | 18676754 | 290538 | GEO |
|  | Bild-Nevins-2006 B | 2.8 | Oncogenic pathway signatures in human cancers as a guide to targeted therapies. | 2006 | Nature | 16273092 | 280683 | GEO |
|  | Rieger-Chu-2004 | 2.57 | Toxicity from radiation therapy associated with abnormal transcriptional responses to DNA damage. | 2004 | Proc Natl Acad Sci U S A | 15096622 | 259974 | GEO |
|  | Wang-Maris-2006 | 1.77 | Integrative genomics identifies distinct molecular classes of neuroblastoma and shows that multiple genes are targeted by regional alterations in DNA copy number. | 2006 | Cancer Res | 16778177 | 264023 | GEO |
|  | Mallon-McKay-2013 | 1.57 | StemCellDB: the human pluripotent stem cell database at the National Institutes of Health. | 2013 | Stem Cell Res | 23117585 | 585265 | GEO |
|  | Wu-Garvey-2007 | 0.71 | The effect of insulin on expression of genes and biochemical pathways in human skeletal muscle. | 2007 | Endocrine | 17709892 | 267109 | GEO |
|  | Boldrick-Relman-2002 | 0.48 | Stereotyped and specific gene expression programs in human innate immune responses to bacteria. | 2002 | Proc Natl Acad Sci U S A | 11805339 | 111707 | SUPPLEMENTARY  _MATERIAL |
|  | Alizadeh-Staudt-2000 | 0.14 | Distinct types of diffuse large B-cell lymphoma identified by gene expression profiling. | 2000 | Nature | 10676951 | 90336 | SUPPLEMENTARY  _MATERIAL |
|  | Rosenwald-Staudt-2001 | 0.02 | Relation of gene expression phenotype to immunoglobulin mutation genotype in B cell chronic lymphocytic leukemia. | 2001 | J Exp Med | 11733578 | 114694 | SUPPLEMENTARY  _MATERIAL |
| **Co-localization** | | 5.5 |  |  |  |  |  |  |
|  | Johnson-Shoemaker-2003 | 5.5 | Genome-wide survey of human alternative pre-mRNA splicing with exon junction microarrays. | 2003 | Science | 14684825 | 426332 | GEO |
| **Physical Interactions** | | 2.55 |  |  |  |  |  |  |
|  | BIOGRID-SMALL-SCALE-STUDIES | 2.31 |  |  |  |  | 58871 | BIOGRID |
|  | IREF-BIOGRID | 0.23 |  |  |  |  | 155470 | IREF |
| **Predicted** | | 1.76 |  |  |  |  |  |  |
|  | Wu-Stein-2010 | 1.76 | A human functional protein interaction network and its application to cancer data analysis. | 2010 | Genome Biol | 20482850 | 87829 | SUPPLEMENTARY  _MATERIAL |
| **Shared protein domains** | | 0.94 |  |  |  |  |  |  |
|  | INTERPRO | 0.9 |  |  |  |  | 608863 | INTERPRO |
|  | PFAM | 0.04 |  |  |  |  | 457054 | PFAM |
| **Genetic Interactions** | | 0.77 |  |  |  |  |  |  |
|  | Lin-Smith-2010 | 0.77 | A genome-wide map of human genetic interactions inferred from radiation hybrid genotypes. | 2010 | Genome Res | 20508145 | 4820370 | SUPPLEMENTARY  _MATERIAL |
| **Pathway** | | 0.42 |  |  |  |  |  |  |
|  | REACTOME | 0.42 |  |  |  |  | 24913 | PATHWAYCOMMONS |

**Supplementary Table 3.** Gene-Gene Interaction Table of GeneMANIA Results

| **Gene 1** | **Gene 2** | **Weight** | **Type** | **Source** |
| --- | --- | --- | --- | --- |
| AURKB | CCNA2 | 6.95E-04 | Co-expression | Alizadeh-Staudt-2000 |
| AURKB | CCNB1 | 5.88E-04 | Co-expression | Alizadeh-Staudt-2000 |
| AURKB | CCNF | 0.001173 | Co-expression | Alizadeh-Staudt-2000 |
| AURKB | CENPE | 0.001232 | Co-expression | Alizadeh-Staudt-2000 |
| AURKB | CENPF | 7.17E-04 | Co-expression | Alizadeh-Staudt-2000 |
| AURKB | KIF23 | 7.97E-04 | Co-expression | Alizadeh-Staudt-2000 |
| AURKB | MKI67 | 0.001098 | Co-expression | Alizadeh-Staudt-2000 |
| AURKB | NEK2 | 7.96E-04 | Co-expression | Alizadeh-Staudt-2000 |
| AURKB | TOP2A | 0.001303 | Co-expression | Alizadeh-Staudt-2000 |
| CCNA2 | CCNB1 | 4.44E-04 | Co-expression | Alizadeh-Staudt-2000 |
| CCNA2 | CCNF | 8.08E-04 | Co-expression | Alizadeh-Staudt-2000 |
| CCNA2 | CENPF | 6.36E-04 | Co-expression | Alizadeh-Staudt-2000 |
| CCNA2 | KIF23 | 5.92E-04 | Co-expression | Alizadeh-Staudt-2000 |
| CCNA2 | NEK2 | 6.23E-04 | Co-expression | Alizadeh-Staudt-2000 |
| CCNB1 | NEK2 | 5.16E-04 | Co-expression | Alizadeh-Staudt-2000 |
| CCNF | CCNB1 | 7.22E-04 | Co-expression | Alizadeh-Staudt-2000 |
| CCNF | CENPF | 9.63E-04 | Co-expression | Alizadeh-Staudt-2000 |
| CCNF | KIF23 | 9.78E-04 | Co-expression | Alizadeh-Staudt-2000 |
| CCNF | NEK2 | 0.001045 | Co-expression | Alizadeh-Staudt-2000 |
| CDC25C | AURKB | 0.001454 | Co-expression | Alizadeh-Staudt-2000 |
| CDC25C | CCNA2 | 0.001024 | Co-expression | Alizadeh-Staudt-2000 |
| CDC25C | CCNB1 | 9.01E-04 | Co-expression | Alizadeh-Staudt-2000 |
| CDC25C | DEPDC1 | 0.001419 | Co-expression | Alizadeh-Staudt-2000 |
| CDC25C | NEK2 | 0.001272 | Co-expression | Alizadeh-Staudt-2000 |
| CDK1 | AURKB | 6.89E-04 | Co-expression | Alizadeh-Staudt-2000 |
| CDK1 | CCNA2 | 5.96E-04 | Co-expression | Alizadeh-Staudt-2000 |
| CDK1 | CCNB1 | 5.05E-04 | Co-expression | Alizadeh-Staudt-2000 |
| CDK1 | CCNF | 9.24E-04 | Co-expression | Alizadeh-Staudt-2000 |
| CDK1 | CENPE | 9.28E-04 | Co-expression | Alizadeh-Staudt-2000 |
| CDK1 | CENPF | 7.13E-04 | Co-expression | Alizadeh-Staudt-2000 |
| CDK1 | KIF23 | 6.84E-04 | Co-expression | Alizadeh-Staudt-2000 |
| CDK1 | MKI67 | 8.87E-04 | Co-expression | Alizadeh-Staudt-2000 |
| CDK1 | NEK2 | 6.74E-04 | Co-expression | Alizadeh-Staudt-2000 |
| CDK1 | TOP2A | 9.18E-04 | Co-expression | Alizadeh-Staudt-2000 |
| CENPE | CCNA2 | 8.12E-04 | Co-expression | Alizadeh-Staudt-2000 |
| CENPE | CCNB1 | 7.30E-04 | Co-expression | Alizadeh-Staudt-2000 |
| CENPE | CENPF | 9.26E-04 | Co-expression | Alizadeh-Staudt-2000 |
| CENPE | KIF23 | 0.001054 | Co-expression | Alizadeh-Staudt-2000 |
| CENPE | NEK2 | 0.001034 | Co-expression | Alizadeh-Staudt-2000 |
| CENPF | CCNB1 | 5.24E-04 | Co-expression | Alizadeh-Staudt-2000 |
| CENPF | NEK2 | 7.24E-04 | Co-expression | Alizadeh-Staudt-2000 |
| DEPDC1 | AURKB | 8.82E-04 | Co-expression | Alizadeh-Staudt-2000 |
| DEPDC1 | CCNA2 | 7.15E-04 | Co-expression | Alizadeh-Staudt-2000 |
| DEPDC1 | CCNB1 | 5.47E-04 | Co-expression | Alizadeh-Staudt-2000 |
| DEPDC1 | CCNF | 0.001105 | Co-expression | Alizadeh-Staudt-2000 |
| DEPDC1 | CDK1 | 7.63E-04 | Co-expression | Alizadeh-Staudt-2000 |
| DEPDC1 | CENPE | 0.001263 | Co-expression | Alizadeh-Staudt-2000 |
| DEPDC1 | CENPF | 7.82E-04 | Co-expression | Alizadeh-Staudt-2000 |
| DEPDC1 | KIF23 | 7.80E-04 | Co-expression | Alizadeh-Staudt-2000 |
| DEPDC1 | MKI67 | 0.001001 | Co-expression | Alizadeh-Staudt-2000 |
| DEPDC1 | NEK2 | 8.18E-04 | Co-expression | Alizadeh-Staudt-2000 |
| DEPDC1 | TOP2A | 0.001213 | Co-expression | Alizadeh-Staudt-2000 |
| HMMR | AURKB | 0.001341 | Co-expression | Alizadeh-Staudt-2000 |
| HMMR | CCNA2 | 0.001115 | Co-expression | Alizadeh-Staudt-2000 |
| HMMR | CCNB1 | 0.001004 | Co-expression | Alizadeh-Staudt-2000 |
| HMMR | CDK1 | 0.001228 | Co-expression | Alizadeh-Staudt-2000 |
| HMMR | CENPE | 0.001857 | Co-expression | Alizadeh-Staudt-2000 |
| HMMR | CENPF | 0.001252 | Co-expression | Alizadeh-Staudt-2000 |
| HMMR | KIF23 | 0.00129 | Co-expression | Alizadeh-Staudt-2000 |
| HMMR | NEK2 | 0.001316 | Co-expression | Alizadeh-Staudt-2000 |
| KIF23 | CCNB1 | 5.54E-04 | Co-expression | Alizadeh-Staudt-2000 |
| KIF23 | CENPF | 7.41E-04 | Co-expression | Alizadeh-Staudt-2000 |
| KIF23 | NEK2 | 7.41E-04 | Co-expression | Alizadeh-Staudt-2000 |
| MKI67 | CCNA2 | 7.33E-04 | Co-expression | Alizadeh-Staudt-2000 |
| MKI67 | CCNB1 | 7.39E-04 | Co-expression | Alizadeh-Staudt-2000 |
| MKI67 | CCNF | 0.001285 | Co-expression | Alizadeh-Staudt-2000 |
| MKI67 | CENPE | 0.00182 | Co-expression | Alizadeh-Staudt-2000 |
| MKI67 | CENPF | 9.25E-04 | Co-expression | Alizadeh-Staudt-2000 |
| MKI67 | KIF23 | 0.001105 | Co-expression | Alizadeh-Staudt-2000 |
| MKI67 | NEK2 | 9.58E-04 | Co-expression | Alizadeh-Staudt-2000 |
| TOP2A | CCNA2 | 8.76E-04 | Co-expression | Alizadeh-Staudt-2000 |
| TOP2A | CCNB1 | 7.27E-04 | Co-expression | Alizadeh-Staudt-2000 |
| TOP2A | CENPE | 0.001963 | Co-expression | Alizadeh-Staudt-2000 |
| TOP2A | CENPF | 9.67E-04 | Co-expression | Alizadeh-Staudt-2000 |
| TOP2A | KIF23 | 0.001224 | Co-expression | Alizadeh-Staudt-2000 |
| TOP2A | MKI67 | 0.001586 | Co-expression | Alizadeh-Staudt-2000 |
| TOP2A | NEK2 | 0.001055 | Co-expression | Alizadeh-Staudt-2000 |
| AURKA | BUB1B | 0.032163 | Co-expression | Bild-Nevins-2006 B |
| AURKA | CCNB1 | 0.028575 | Co-expression | Bild-Nevins-2006 B |
| AURKA | GINS1 | 0.026667 | Co-expression | Bild-Nevins-2006 B |
| AURKA | KIF23 | 0.035716 | Co-expression | Bild-Nevins-2006 B |
| AURKA | NDC80 | 0.022436 | Co-expression | Bild-Nevins-2006 B |
| AURKB | AURKA | 0.028011 | Co-expression | Bild-Nevins-2006 B |
| AURKB | BUB1B | 0.015509 | Co-expression | Bild-Nevins-2006 B |
| AURKB | CCNB1 | 0.017561 | Co-expression | Bild-Nevins-2006 B |
| AURKB | CENPE | 0.030494 | Co-expression | Bild-Nevins-2006 B |
| AURKB | CENPF | 0.026285 | Co-expression | Bild-Nevins-2006 B |
| AURKB | GINS1 | 0.017713 | Co-expression | Bild-Nevins-2006 B |
| AURKB | KIF23 | 0.021682 | Co-expression | Bild-Nevins-2006 B |
| AURKB | MKI67 | 0.031401 | Co-expression | Bild-Nevins-2006 B |
| AURKB | NDC80 | 0.015678 | Co-expression | Bild-Nevins-2006 B |
| BUB1B | GINS1 | 0.01609 | Co-expression | Bild-Nevins-2006 B |
| CCNA2 | BUB1B | 0.023526 | Co-expression | Bild-Nevins-2006 B |
| CCNA2 | CCNB1 | 0.024544 | Co-expression | Bild-Nevins-2006 B |
| CCNA2 | KIF23 | 0.027368 | Co-expression | Bild-Nevins-2006 B |
| CCNA2 | NEK2 | 0.042162 | Co-expression | Bild-Nevins-2006 B |
| CCNB1 | BUB1B | 0.018305 | Co-expression | Bild-Nevins-2006 B |
| CCNB1 | GINS1 | 0.014702 | Co-expression | Bild-Nevins-2006 B |
| CCNB1 | NEK2 | 0.020137 | Co-expression | Bild-Nevins-2006 B |
| CDC25C | AURKB | 0.028236 | Co-expression | Bild-Nevins-2006 B |
| CDC25C | CENPF | 0.037193 | Co-expression | Bild-Nevins-2006 B |
| CDC25C | MKI67 | 0.044011 | Co-expression | Bild-Nevins-2006 B |
| CDK1 | AURKB | 0.031834 | Co-expression | Bild-Nevins-2006 B |
| CDK1 | BUB1B | 0.027311 | Co-expression | Bild-Nevins-2006 B |
| CDK1 | CCNA2 | 0.052857 | Co-expression | Bild-Nevins-2006 B |
| CDK1 | CCNB1 | 0.030357 | Co-expression | Bild-Nevins-2006 B |
| CDK1 | CENPE | 0.043629 | Co-expression | Bild-Nevins-2006 B |
| CDK1 | GINS1 | 0.026947 | Co-expression | Bild-Nevins-2006 B |
| CDK1 | KIF11 | 0.030504 | Co-expression | Bild-Nevins-2006 B |
| CDK1 | KIF23 | 0.034105 | Co-expression | Bild-Nevins-2006 B |
| CDK1 | MKI67 | 0.044797 | Co-expression | Bild-Nevins-2006 B |
| CDK1 | NDC80 | 0.0202 | Co-expression | Bild-Nevins-2006 B |
| CDK1 | TOP2A | 0.048912 | Co-expression | Bild-Nevins-2006 B |
| CENPE | BUB1B | 0.025303 | Co-expression | Bild-Nevins-2006 B |
| CENPE | CCNA2 | 0.041628 | Co-expression | Bild-Nevins-2006 B |
| CENPE | CCNB1 | 0.026323 | Co-expression | Bild-Nevins-2006 B |
| CENPE | CENPF | 0.029949 | Co-expression | Bild-Nevins-2006 B |
| CENPE | GINS1 | 0.024953 | Co-expression | Bild-Nevins-2006 B |
| CENPE | KIF23 | 0.032537 | Co-expression | Bild-Nevins-2006 B |
| CENPF | BUB1B | 0.02504 | Co-expression | Bild-Nevins-2006 B |
| CENPF | CCNB1 | 0.01993 | Co-expression | Bild-Nevins-2006 B |
| CENPF | GINS1 | 0.023426 | Co-expression | Bild-Nevins-2006 B |
| CENPF | NEK2 | 0.027518 | Co-expression | Bild-Nevins-2006 B |
| HMMR | AURKB | 0.027432 | Co-expression | Bild-Nevins-2006 B |
| HMMR | BUB1B | 0.022826 | Co-expression | Bild-Nevins-2006 B |
| HMMR | CCNA2 | 0.041758 | Co-expression | Bild-Nevins-2006 B |
| HMMR | CCNB1 | 0.026972 | Co-expression | Bild-Nevins-2006 B |
| HMMR | CDK1 | 0.059723 | Co-expression | Bild-Nevins-2006 B |
| HMMR | CENPE | 0.046764 | Co-expression | Bild-Nevins-2006 B |
| HMMR | GINS1 | 0.021835 | Co-expression | Bild-Nevins-2006 B |
| HMMR | KIF11 | 0.029361 | Co-expression | Bild-Nevins-2006 B |
| HMMR | KIF23 | 0.032448 | Co-expression | Bild-Nevins-2006 B |
| HMMR | MKI67 | 0.043639 | Co-expression | Bild-Nevins-2006 B |
| HMMR | NDC80 | 0.020234 | Co-expression | Bild-Nevins-2006 B |
| KIF11 | AURKA | 0.02731 | Co-expression | Bild-Nevins-2006 B |
| KIF11 | AURKB | 0.016414 | Co-expression | Bild-Nevins-2006 B |
| KIF11 | BUB1B | 0.01717 | Co-expression | Bild-Nevins-2006 B |
| KIF11 | CCNA2 | 0.024688 | Co-expression | Bild-Nevins-2006 B |
| KIF11 | CCNB1 | 0.019969 | Co-expression | Bild-Nevins-2006 B |
| KIF11 | CENPE | 0.025629 | Co-expression | Bild-Nevins-2006 B |
| KIF11 | CENPF | 0.022903 | Co-expression | Bild-Nevins-2006 B |
| KIF11 | GINS1 | 0.01606 | Co-expression | Bild-Nevins-2006 B |
| KIF11 | KIF23 | 0.025034 | Co-expression | Bild-Nevins-2006 B |
| KIF11 | MKI67 | 0.029641 | Co-expression | Bild-Nevins-2006 B |
| KIF11 | NDC80 | 0.011851 | Co-expression | Bild-Nevins-2006 B |
| KIF11 | TOP2A | 0.024768 | Co-expression | Bild-Nevins-2006 B |
| KIF23 | BUB1B | 0.02408 | Co-expression | Bild-Nevins-2006 B |
| KIF23 | CCNB1 | 0.023737 | Co-expression | Bild-Nevins-2006 B |
| KIF23 | CENPF | 0.024902 | Co-expression | Bild-Nevins-2006 B |
| KIF23 | GINS1 | 0.017555 | Co-expression | Bild-Nevins-2006 B |
| MKI67 | BUB1B | 0.025643 | Co-expression | Bild-Nevins-2006 B |
| MKI67 | CCNA2 | 0.043026 | Co-expression | Bild-Nevins-2006 B |
| MKI67 | CCNB1 | 0.025539 | Co-expression | Bild-Nevins-2006 B |
| MKI67 | CENPE | 0.045496 | Co-expression | Bild-Nevins-2006 B |
| MKI67 | CENPF | 0.036162 | Co-expression | Bild-Nevins-2006 B |
| MKI67 | GINS1 | 0.025028 | Co-expression | Bild-Nevins-2006 B |
| MKI67 | KIF23 | 0.033366 | Co-expression | Bild-Nevins-2006 B |
| MKI67 | NDC80 | 0.021024 | Co-expression | Bild-Nevins-2006 B |
| NDC80 | BUB1B | 0.016498 | Co-expression | Bild-Nevins-2006 B |
| NDC80 | CCNB1 | 0.012233 | Co-expression | Bild-Nevins-2006 B |
| NDC80 | CENPE | 0.022708 | Co-expression | Bild-Nevins-2006 B |
| NDC80 | CENPF | 0.021223 | Co-expression | Bild-Nevins-2006 B |
| NDC80 | GINS1 | 0.01573 | Co-expression | Bild-Nevins-2006 B |
| NDC80 | KIF23 | 0.016008 | Co-expression | Bild-Nevins-2006 B |
| SMC4 | CCNA2 | 0.019324 | Co-expression | Bild-Nevins-2006 B |
| SMC4 | CCNB1 | 0.013803 | Co-expression | Bild-Nevins-2006 B |
| SMC4 | CDK1 | 0.028568 | Co-expression | Bild-Nevins-2006 B |
| SMC4 | HMMR | 0.023703 | Co-expression | Bild-Nevins-2006 B |
| SMC4 | KIF11 | 0.015851 | Co-expression | Bild-Nevins-2006 B |
| SMC4 | KIF23 | 0.017242 | Co-expression | Bild-Nevins-2006 B |
| SMC4 | MKI67 | 0.018459 | Co-expression | Bild-Nevins-2006 B |
| SMC4 | ZWINT | 0.013637 | Co-expression | Bild-Nevins-2006 B |
| TOP2A | BUB1B | 0.021821 | Co-expression | Bild-Nevins-2006 B |
| TOP2A | CCNB1 | 0.024002 | Co-expression | Bild-Nevins-2006 B |
| TOP2A | CENPE | 0.034235 | Co-expression | Bild-Nevins-2006 B |
| TOP2A | CENPF | 0.032073 | Co-expression | Bild-Nevins-2006 B |
| TOP2A | GINS1 | 0.020345 | Co-expression | Bild-Nevins-2006 B |
| TOP2A | KIF23 | 0.03012 | Co-expression | Bild-Nevins-2006 B |
| TOP2A | MKI67 | 0.039854 | Co-expression | Bild-Nevins-2006 B |
| ZWINT | BUB1B | 0.020929 | Co-expression | Bild-Nevins-2006 B |
| ZWINT | CCNB1 | 0.018262 | Co-expression | Bild-Nevins-2006 B |
| ZWINT | CDK1 | 0.029767 | Co-expression | Bild-Nevins-2006 B |
| ZWINT | CENPE | 0.024683 | Co-expression | Bild-Nevins-2006 B |
| ZWINT | CENPF | 0.02459 | Co-expression | Bild-Nevins-2006 B |
| ZWINT | GINS1 | 0.019739 | Co-expression | Bild-Nevins-2006 B |
| ZWINT | HMMR | 0.027643 | Co-expression | Bild-Nevins-2006 B |
| ZWINT | KIF11 | 0.019824 | Co-expression | Bild-Nevins-2006 B |
| ZWINT | KIF23 | 0.02118 | Co-expression | Bild-Nevins-2006 B |
| ZWINT | MKI67 | 0.027304 | Co-expression | Bild-Nevins-2006 B |
| ZWINT | NDC80 | 0.013738 | Co-expression | Bild-Nevins-2006 B |
| ZWINT | TOP2A | 0.025252 | Co-expression | Bild-Nevins-2006 B |
| AURKB | CCNA2 | 0.00469 | Co-expression | Boldrick-Relman-2002 |
| AURKB | CENPE | 0.004375 | Co-expression | Boldrick-Relman-2002 |
| AURKB | CENPF | 0.006 | Co-expression | Boldrick-Relman-2002 |
| AURKB | MKI67 | 0.00255 | Co-expression | Boldrick-Relman-2002 |
| AURKB | TOP2A | 0.008982 | Co-expression | Boldrick-Relman-2002 |
| CCNA2 | CCNB1 | 0.002089 | Co-expression | Boldrick-Relman-2002 |
| CCNA2 | KIF23 | 0.003013 | Co-expression | Boldrick-Relman-2002 |
| CCNB1 | NEK2 | 0.0023 | Co-expression | Boldrick-Relman-2002 |
| CCNF | CCNB1 | 0.002257 | Co-expression | Boldrick-Relman-2002 |
| CCNF | KIF23 | 0.003414 | Co-expression | Boldrick-Relman-2002 |
| CCNF | NEK2 | 0.005613 | Co-expression | Boldrick-Relman-2002 |
| CDC25C | CCNB1 | 0.00313 | Co-expression | Boldrick-Relman-2002 |
| CDC25C | CCNF | 0.008687 | Co-expression | Boldrick-Relman-2002 |
| CDC25C | CENPE | 0.006543 | Co-expression | Boldrick-Relman-2002 |
| CDK1 | CCNB1 | 0.001515 | Co-expression | Boldrick-Relman-2002 |
| CDK1 | KIF23 | 0.002335 | Co-expression | Boldrick-Relman-2002 |
| CDK1 | NEK2 | 0.00367 | Co-expression | Boldrick-Relman-2002 |
| CENPE | CCNA2 | 0.004364 | Co-expression | Boldrick-Relman-2002 |
| CENPE | CCNB1 | 0.001845 | Co-expression | Boldrick-Relman-2002 |
| CENPE | KIF23 | 0.002726 | Co-expression | Boldrick-Relman-2002 |
| CENPF | CCNB1 | 0.002233 | Co-expression | Boldrick-Relman-2002 |
| DEPDC1 | CCNB1 | 0.002253 | Co-expression | Boldrick-Relman-2002 |
| DEPDC1 | HMMR | 0.006358 | Co-expression | Boldrick-Relman-2002 |
| DEPDC1 | KIF23 | 0.003638 | Co-expression | Boldrick-Relman-2002 |
| DEPDC1 | MKI67 | 0.002973 | Co-expression | Boldrick-Relman-2002 |
| HMMR | AURKB | 0.005301 | Co-expression | Boldrick-Relman-2002 |
| HMMR | CCNB1 | 0.00237 | Co-expression | Boldrick-Relman-2002 |
| HMMR | KIF23 | 0.003616 | Co-expression | Boldrick-Relman-2002 |
| HMMR | NEK2 | 0.005626 | Co-expression | Boldrick-Relman-2002 |
| KIF23 | CCNB1 | 0.001467 | Co-expression | Boldrick-Relman-2002 |
| KIF23 | NEK2 | 0.003603 | Co-expression | Boldrick-Relman-2002 |
| MKI67 | CCNA2 | 0.002488 | Co-expression | Boldrick-Relman-2002 |
| MKI67 | CCNB1 | 0.001105 | Co-expression | Boldrick-Relman-2002 |
| MKI67 | CENPE | 0.002243 | Co-expression | Boldrick-Relman-2002 |
| MKI67 | CENPF | 0.003099 | Co-expression | Boldrick-Relman-2002 |
| MKI67 | KIF23 | 0.001662 | Co-expression | Boldrick-Relman-2002 |
| TOP2A | CCNA2 | 0.00701 | Co-expression | Boldrick-Relman-2002 |
| TOP2A | CCNB1 | 0.003126 | Co-expression | Boldrick-Relman-2002 |
| TOP2A | CENPE | 0.006836 | Co-expression | Boldrick-Relman-2002 |
| TOP2A | CENPF | 0.008926 | Co-expression | Boldrick-Relman-2002 |
| TOP2A | KIF23 | 0.004809 | Co-expression | Boldrick-Relman-2002 |
| TOP2A | MKI67 | 0.004039 | Co-expression | Boldrick-Relman-2002 |
| AURKA | BUB1B | 0.015558 | Co-expression | Burington-Shaughnessy-2008 |
| AURKA | CCNA2 | 0.01863 | Co-expression | Burington-Shaughnessy-2008 |
| AURKA | CCNB1 | 0.015862 | Co-expression | Burington-Shaughnessy-2008 |
| AURKA | CCNF | 0.038699 | Co-expression | Burington-Shaughnessy-2008 |
| AURKA | CENPE | 0.016051 | Co-expression | Burington-Shaughnessy-2008 |
| AURKA | CENPF | 0.01808 | Co-expression | Burington-Shaughnessy-2008 |
| AURKA | GINS1 | 0.013285 | Co-expression | Burington-Shaughnessy-2008 |
| AURKA | KIF23 | 0.018622 | Co-expression | Burington-Shaughnessy-2008 |
| AURKA | MKI67 | 0.018012 | Co-expression | Burington-Shaughnessy-2008 |
| AURKA | NDC80 | 0.016539 | Co-expression | Burington-Shaughnessy-2008 |
| AURKA | NEK2 | 0.025478 | Co-expression | Burington-Shaughnessy-2008 |
| AURKB | AURKA | 0.019681 | Co-expression | Burington-Shaughnessy-2008 |
| AURKB | CCNA2 | 0.014602 | Co-expression | Burington-Shaughnessy-2008 |
| AURKB | CCNB1 | 0.012562 | Co-expression | Burington-Shaughnessy-2008 |
| AURKB | GINS1 | 0.012369 | Co-expression | Burington-Shaughnessy-2008 |
| AURKB | TOP2A | 0.012629 | Co-expression | Burington-Shaughnessy-2008 |
| BUB1B | GINS1 | 0.011141 | Co-expression | Burington-Shaughnessy-2008 |
| BUB1B | NEK2 | 0.018422 | Co-expression | Burington-Shaughnessy-2008 |
| CCNA2 | BUB1B | 0.014321 | Co-expression | Burington-Shaughnessy-2008 |
| CCNA2 | CCNB1 | 0.014093 | Co-expression | Burington-Shaughnessy-2008 |
| CCNA2 | CCNF | 0.035178 | Co-expression | Burington-Shaughnessy-2008 |
| CCNA2 | CENPF | 0.016471 | Co-expression | Burington-Shaughnessy-2008 |
| CCNA2 | GINS1 | 0.011857 | Co-expression | Burington-Shaughnessy-2008 |
| CCNA2 | KIF23 | 0.016497 | Co-expression | Burington-Shaughnessy-2008 |
| CCNA2 | NEK2 | 0.025725 | Co-expression | Burington-Shaughnessy-2008 |
| CCNB1 | BUB1B | 0.013145 | Co-expression | Burington-Shaughnessy-2008 |
| CCNB1 | GINS1 | 0.01041 | Co-expression | Burington-Shaughnessy-2008 |
| CCNB1 | NEK2 | 0.018976 | Co-expression | Burington-Shaughnessy-2008 |
| CCNF | CCNB1 | 0.024775 | Co-expression | Burington-Shaughnessy-2008 |
| CCNF | CENPF | 0.038539 | Co-expression | Burington-Shaughnessy-2008 |
| CCNF | GINS1 | 0.022022 | Co-expression | Burington-Shaughnessy-2008 |
| CCNF | KIF23 | 0.034092 | Co-expression | Burington-Shaughnessy-2008 |
| CCNF | NEK2 | 0.064645 | Co-expression | Burington-Shaughnessy-2008 |
| CDC25C | AURKA | 0.029739 | Co-expression | Burington-Shaughnessy-2008 |
| CDC25C | BUB1B | 0.023813 | Co-expression | Burington-Shaughnessy-2008 |
| CDC25C | CCNA2 | 0.027528 | Co-expression | Burington-Shaughnessy-2008 |
| CDC25C | CCNB1 | 0.022045 | Co-expression | Burington-Shaughnessy-2008 |
| CDC25C | CCNF | 0.072543 | Co-expression | Burington-Shaughnessy-2008 |
| CDC25C | CDK1 | 0.032951 | Co-expression | Burington-Shaughnessy-2008 |
| CDC25C | CENPF | 0.027444 | Co-expression | Burington-Shaughnessy-2008 |
| CDC25C | GINS1 | 0.018015 | Co-expression | Burington-Shaughnessy-2008 |
| CDC25C | HMMR | 0.033702 | Co-expression | Burington-Shaughnessy-2008 |
| CDC25C | KIF11 | 0.020011 | Co-expression | Burington-Shaughnessy-2008 |
| CDC25C | KIF23 | 0.031462 | Co-expression | Burington-Shaughnessy-2008 |
| CDC25C | MKI67 | 0.028351 | Co-expression | Burington-Shaughnessy-2008 |
| CDC25C | NEK2 | 0.041499 | Co-expression | Burington-Shaughnessy-2008 |
| CDC25C | TOP2A | 0.023809 | Co-expression | Burington-Shaughnessy-2008 |
| CDC25C | ZWINT | 0.020508 | Co-expression | Burington-Shaughnessy-2008 |
| CDK1 | AURKA | 0.021875 | Co-expression | Burington-Shaughnessy-2008 |
| CDK1 | BUB1B | 0.017205 | Co-expression | Burington-Shaughnessy-2008 |
| CDK1 | CCNA2 | 0.020414 | Co-expression | Burington-Shaughnessy-2008 |
| CDK1 | CCNB1 | 0.016107 | Co-expression | Burington-Shaughnessy-2008 |
| CDK1 | CCNF | 0.04183 | Co-expression | Burington-Shaughnessy-2008 |
| CDK1 | CENPE | 0.019121 | Co-expression | Burington-Shaughnessy-2008 |
| CDK1 | CENPF | 0.020143 | Co-expression | Burington-Shaughnessy-2008 |
| CDK1 | GINS1 | 0.013649 | Co-expression | Burington-Shaughnessy-2008 |
| CDK1 | KIF11 | 0.015088 | Co-expression | Burington-Shaughnessy-2008 |
| CDK1 | KIF23 | 0.019475 | Co-expression | Burington-Shaughnessy-2008 |
| CDK1 | MKI67 | 0.022016 | Co-expression | Burington-Shaughnessy-2008 |
| CDK1 | NEK2 | 0.030814 | Co-expression | Burington-Shaughnessy-2008 |
| CDK1 | TOP2A | 0.018022 | Co-expression | Burington-Shaughnessy-2008 |
| CENPE | BUB1B | 0.014008 | Co-expression | Burington-Shaughnessy-2008 |
| CENPE | CCNA2 | 0.015495 | Co-expression | Burington-Shaughnessy-2008 |
| CENPE | CCNB1 | 0.01293 | Co-expression | Burington-Shaughnessy-2008 |
| CENPE | CENPF | 0.016265 | Co-expression | Burington-Shaughnessy-2008 |
| CENPE | GINS1 | 0.011741 | Co-expression | Burington-Shaughnessy-2008 |
| CENPE | KIF23 | 0.015197 | Co-expression | Burington-Shaughnessy-2008 |
| CENPE | NEK2 | 0.023389 | Co-expression | Burington-Shaughnessy-2008 |
| CENPF | BUB1B | 0.014565 | Co-expression | Burington-Shaughnessy-2008 |
| CENPF | CCNB1 | 0.013004 | Co-expression | Burington-Shaughnessy-2008 |
| CENPF | GINS1 | 0.011923 | Co-expression | Burington-Shaughnessy-2008 |
| CENPF | NEK2 | 0.024508 | Co-expression | Burington-Shaughnessy-2008 |
| GINS1 | NEK2 | 0.015379 | Co-expression | Burington-Shaughnessy-2008 |
| HMMR | AURKA | 0.021627 | Co-expression | Burington-Shaughnessy-2008 |
| HMMR | BUB1B | 0.018994 | Co-expression | Burington-Shaughnessy-2008 |
| HMMR | CCNA2 | 0.019983 | Co-expression | Burington-Shaughnessy-2008 |
| HMMR | CCNB1 | 0.016757 | Co-expression | Burington-Shaughnessy-2008 |
| HMMR | CDK1 | 0.023297 | Co-expression | Burington-Shaughnessy-2008 |
| HMMR | CENPF | 0.018268 | Co-expression | Burington-Shaughnessy-2008 |
| HMMR | GINS1 | 0.013034 | Co-expression | Burington-Shaughnessy-2008 |
| HMMR | KIF11 | 0.015344 | Co-expression | Burington-Shaughnessy-2008 |
| HMMR | KIF23 | 0.022437 | Co-expression | Burington-Shaughnessy-2008 |
| HMMR | MKI67 | 0.021086 | Co-expression | Burington-Shaughnessy-2008 |
| HMMR | NDC80 | 0.018771 | Co-expression | Burington-Shaughnessy-2008 |
| HMMR | NEK2 | 0.024851 | Co-expression | Burington-Shaughnessy-2008 |
| HMMR | TOP2A | 0.018119 | Co-expression | Burington-Shaughnessy-2008 |
| KIF11 | AURKA | 0.013108 | Co-expression | Burington-Shaughnessy-2008 |
| KIF11 | BUB1B | 0.012333 | Co-expression | Burington-Shaughnessy-2008 |
| KIF11 | CCNA2 | 0.01233 | Co-expression | Burington-Shaughnessy-2008 |
| KIF11 | CCNB1 | 0.010622 | Co-expression | Burington-Shaughnessy-2008 |
| KIF11 | CENPE | 0.012721 | Co-expression | Burington-Shaughnessy-2008 |
| KIF11 | CENPF | 0.012539 | Co-expression | Burington-Shaughnessy-2008 |
| KIF11 | GINS1 | 0.009402 | Co-expression | Burington-Shaughnessy-2008 |
| KIF11 | KIF23 | 0.013336 | Co-expression | Burington-Shaughnessy-2008 |
| KIF11 | MKI67 | 0.014845 | Co-expression | Burington-Shaughnessy-2008 |
| KIF11 | NDC80 | 0.012825 | Co-expression | Burington-Shaughnessy-2008 |
| KIF11 | NEK2 | 0.016009 | Co-expression | Burington-Shaughnessy-2008 |
| KIF11 | TOP2A | 0.011797 | Co-expression | Burington-Shaughnessy-2008 |
| KIF23 | BUB1B | 0.017644 | Co-expression | Burington-Shaughnessy-2008 |
| KIF23 | CCNB1 | 0.01478 | Co-expression | Burington-Shaughnessy-2008 |
| KIF23 | CENPF | 0.01692 | Co-expression | Burington-Shaughnessy-2008 |
| KIF23 | GINS1 | 0.012261 | Co-expression | Burington-Shaughnessy-2008 |
| KIF23 | NEK2 | 0.022564 | Co-expression | Burington-Shaughnessy-2008 |
| MKI67 | BUB1B | 0.017035 | Co-expression | Burington-Shaughnessy-2008 |
| MKI67 | CCNA2 | 0.017959 | Co-expression | Burington-Shaughnessy-2008 |
| MKI67 | CCNB1 | 0.014736 | Co-expression | Burington-Shaughnessy-2008 |
| MKI67 | CCNF | 0.036084 | Co-expression | Burington-Shaughnessy-2008 |
| MKI67 | CENPE | 0.018346 | Co-expression | Burington-Shaughnessy-2008 |
| MKI67 | CENPF | 0.0189 | Co-expression | Burington-Shaughnessy-2008 |
| MKI67 | GINS1 | 0.012927 | Co-expression | Burington-Shaughnessy-2008 |
| MKI67 | KIF23 | 0.018085 | Co-expression | Burington-Shaughnessy-2008 |
| MKI67 | NDC80 | 0.016821 | Co-expression | Burington-Shaughnessy-2008 |
| MKI67 | NEK2 | 0.024237 | Co-expression | Burington-Shaughnessy-2008 |
| NDC80 | BUB1B | 0.015334 | Co-expression | Burington-Shaughnessy-2008 |
| NDC80 | CCNB1 | 0.013989 | Co-expression | Burington-Shaughnessy-2008 |
| NDC80 | CENPE | 0.014914 | Co-expression | Burington-Shaughnessy-2008 |
| NDC80 | GINS1 | 0.011437 | Co-expression | Burington-Shaughnessy-2008 |
| NDC80 | KIF23 | 0.015645 | Co-expression | Burington-Shaughnessy-2008 |
| SMC4 | AURKA | 0.019427 | Co-expression | Burington-Shaughnessy-2008 |
| SMC4 | BUB1B | 0.017512 | Co-expression | Burington-Shaughnessy-2008 |
| SMC4 | CCNA2 | 0.018071 | Co-expression | Burington-Shaughnessy-2008 |
| SMC4 | CCNB1 | 0.014256 | Co-expression | Burington-Shaughnessy-2008 |
| SMC4 | CDK1 | 0.022522 | Co-expression | Burington-Shaughnessy-2008 |
| SMC4 | CENPE | 0.019321 | Co-expression | Burington-Shaughnessy-2008 |
| SMC4 | CENPF | 0.019246 | Co-expression | Burington-Shaughnessy-2008 |
| SMC4 | HMMR | 0.022303 | Co-expression | Burington-Shaughnessy-2008 |
| SMC4 | KIF11 | 0.014804 | Co-expression | Burington-Shaughnessy-2008 |
| SMC4 | KIF23 | 0.019635 | Co-expression | Burington-Shaughnessy-2008 |
| SMC4 | MKI67 | 0.019211 | Co-expression | Burington-Shaughnessy-2008 |
| SMC4 | NDC80 | 0.018013 | Co-expression | Burington-Shaughnessy-2008 |
| SMC4 | TOP2A | 0.018271 | Co-expression | Burington-Shaughnessy-2008 |
| SMC4 | ZWINT | 0.01629 | Co-expression | Burington-Shaughnessy-2008 |
| TOP2A | AURKA | 0.016667 | Co-expression | Burington-Shaughnessy-2008 |
| TOP2A | BUB1B | 0.013572 | Co-expression | Burington-Shaughnessy-2008 |
| TOP2A | CCNA2 | 0.015149 | Co-expression | Burington-Shaughnessy-2008 |
| TOP2A | CCNB1 | 0.012496 | Co-expression | Burington-Shaughnessy-2008 |
| TOP2A | CCNF | 0.028317 | Co-expression | Burington-Shaughnessy-2008 |
| TOP2A | CENPE | 0.014415 | Co-expression | Burington-Shaughnessy-2008 |
| TOP2A | CENPF | 0.015418 | Co-expression | Burington-Shaughnessy-2008 |
| TOP2A | GINS1 | 0.010759 | Co-expression | Burington-Shaughnessy-2008 |
| TOP2A | KIF23 | 0.015938 | Co-expression | Burington-Shaughnessy-2008 |
| TOP2A | MKI67 | 0.016347 | Co-expression | Burington-Shaughnessy-2008 |
| TOP2A | NDC80 | 0.013976 | Co-expression | Burington-Shaughnessy-2008 |
| TOP2A | NEK2 | 0.021294 | Co-expression | Burington-Shaughnessy-2008 |
| ZWINT | AURKA | 0.014253 | Co-expression | Burington-Shaughnessy-2008 |
| ZWINT | AURKB | 0.011769 | Co-expression | Burington-Shaughnessy-2008 |
| ZWINT | BUB1B | 0.01285 | Co-expression | Burington-Shaughnessy-2008 |
| ZWINT | CCNA2 | 0.013149 | Co-expression | Burington-Shaughnessy-2008 |
| ZWINT | CCNB1 | 0.011042 | Co-expression | Burington-Shaughnessy-2008 |
| ZWINT | CDK1 | 0.015962 | Co-expression | Burington-Shaughnessy-2008 |
| ZWINT | CENPE | 0.013735 | Co-expression | Burington-Shaughnessy-2008 |
| ZWINT | CENPF | 0.013237 | Co-expression | Burington-Shaughnessy-2008 |
| ZWINT | GINS1 | 0.010318 | Co-expression | Burington-Shaughnessy-2008 |
| ZWINT | HMMR | 0.015923 | Co-expression | Burington-Shaughnessy-2008 |
| ZWINT | KIF11 | 0.01132 | Co-expression | Burington-Shaughnessy-2008 |
| ZWINT | KIF23 | 0.014127 | Co-expression | Burington-Shaughnessy-2008 |
| ZWINT | MKI67 | 0.014832 | Co-expression | Burington-Shaughnessy-2008 |
| ZWINT | NDC80 | 0.013372 | Co-expression | Burington-Shaughnessy-2008 |
| ZWINT | NEK2 | 0.016917 | Co-expression | Burington-Shaughnessy-2008 |
| ZWINT | TOP2A | 0.01246 | Co-expression | Burington-Shaughnessy-2008 |
| ASPM | AURKA | 0.068529 | Co-expression | Dobbin-Giordano-2005 |
| ASPM | CCNA2 | 0.035041 | Co-expression | Dobbin-Giordano-2005 |
| ASPM | CCNB1 | 0.021113 | Co-expression | Dobbin-Giordano-2005 |
| ASPM | CENPE | 0.029469 | Co-expression | Dobbin-Giordano-2005 |
| ASPM | CENPF | 0.091747 | Co-expression | Dobbin-Giordano-2005 |
| ASPM | HMMR | 0.046307 | Co-expression | Dobbin-Giordano-2005 |
| ASPM | KIF11 | 0.024096 | Co-expression | Dobbin-Giordano-2005 |
| ASPM | KIF20A | 0.030108 | Co-expression | Dobbin-Giordano-2005 |
| ASPM | NEK2 | 0.039875 | Co-expression | Dobbin-Giordano-2005 |
| ASPM | PRC1 | 0.019382 | Co-expression | Dobbin-Giordano-2005 |
| ASPM | SMC4 | 0.082997 | Co-expression | Dobbin-Giordano-2005 |
| ASPM | TOP2A | 0.024887 | Co-expression | Dobbin-Giordano-2005 |
| ASPM | ZWINT | 0.02226 | Co-expression | Dobbin-Giordano-2005 |
| AURKA | CCNA2 | 0.065184 | Co-expression | Dobbin-Giordano-2005 |
| AURKA | CCNB1 | 0.04028 | Co-expression | Dobbin-Giordano-2005 |
| AURKA | CENPE | 0.055345 | Co-expression | Dobbin-Giordano-2005 |
| AURKA | KIF20A | 0.056996 | Co-expression | Dobbin-Giordano-2005 |
| AURKA | KIF23 | 0.046365 | Co-expression | Dobbin-Giordano-2005 |
| AURKA | MKI67 | 0.111227 | Co-expression | Dobbin-Giordano-2005 |
| AURKA | PRC1 | 0.035369 | Co-expression | Dobbin-Giordano-2005 |
| AURKB | CCNB1 | 0.013052 | Co-expression | Dobbin-Giordano-2005 |
| AURKB | CCNF | 0.034334 | Co-expression | Dobbin-Giordano-2005 |
| AURKB | NEK2 | 0.028371 | Co-expression | Dobbin-Giordano-2005 |
| AURKB | PRC1 | 0.01466 | Co-expression | Dobbin-Giordano-2005 |
| BUB1B | NUSAP1 | 0.041281 | Co-expression | Dobbin-Giordano-2005 |
| BUB1B | PRC1 | 0.019205 | Co-expression | Dobbin-Giordano-2005 |
| CCNA2 | BUB1B | 0.034739 | Co-expression | Dobbin-Giordano-2005 |
| CCNA2 | CCNB1 | 0.022109 | Co-expression | Dobbin-Giordano-2005 |
| CCNA2 | CCNF | 0.046341 | Co-expression | Dobbin-Giordano-2005 |
| CCNA2 | CENPF | 0.067903 | Co-expression | Dobbin-Giordano-2005 |
| CCNA2 | KIF23 | 0.027252 | Co-expression | Dobbin-Giordano-2005 |
| CCNA2 | NEK2 | 0.040072 | Co-expression | Dobbin-Giordano-2005 |
| CCNA2 | PRC1 | 0.021792 | Co-expression | Dobbin-Giordano-2005 |
| CCNB1 | BUB1B | 0.019006 | Co-expression | Dobbin-Giordano-2005 |
| CCNB1 | KIF20A | 0.01494 | Co-expression | Dobbin-Giordano-2005 |
| CCNB1 | NEK2 | 0.023882 | Co-expression | Dobbin-Giordano-2005 |
| CCNB1 | PRC1 | 0.012562 | Co-expression | Dobbin-Giordano-2005 |
| CCNF | BUB1B | 0.053422 | Co-expression | Dobbin-Giordano-2005 |
| CCNF | NUSAP1 | 0.047156 | Co-expression | Dobbin-Giordano-2005 |
| CCNF | PRC1 | 0.027566 | Co-expression | Dobbin-Giordano-2005 |
| CDC25C | CDK1 | 0.058732 | Co-expression | Dobbin-Giordano-2005 |
| CDC25C | CENPE | 0.056644 | Co-expression | Dobbin-Giordano-2005 |
| CDC25C | DEPDC1 | 0.066391 | Co-expression | Dobbin-Giordano-2005 |
| CDC25C | KIF11 | 0.053981 | Co-expression | Dobbin-Giordano-2005 |
| CDC25C | KIF20A | 0.056016 | Co-expression | Dobbin-Giordano-2005 |
| CDC25C | KIF23 | 0.052282 | Co-expression | Dobbin-Giordano-2005 |
| CDC25C | KIF4A | 0.038297 | Co-expression | Dobbin-Giordano-2005 |
| CDC25C | NEK2 | 0.081479 | Co-expression | Dobbin-Giordano-2005 |
| CDC25C | NUSAP1 | 0.070219 | Co-expression | Dobbin-Giordano-2005 |
| CDC25C | PRC1 | 0.038904 | Co-expression | Dobbin-Giordano-2005 |
| CDC25C | TOP2A | 0.048166 | Co-expression | Dobbin-Giordano-2005 |
| CDC25C | ZWINT | 0.047804 | Co-expression | Dobbin-Giordano-2005 |
| CDCA3 | CCNA2 | 0.026111 | Co-expression | Dobbin-Giordano-2005 |
| CDCA3 | CCNB1 | 0.016142 | Co-expression | Dobbin-Giordano-2005 |
| CDCA3 | CENPE | 0.019814 | Co-expression | Dobbin-Giordano-2005 |
| CDCA3 | KIF23 | 0.019744 | Co-expression | Dobbin-Giordano-2005 |
| CDCA3 | NEK2 | 0.029095 | Co-expression | Dobbin-Giordano-2005 |
| CDCA3 | PRC1 | 0.015392 | Co-expression | Dobbin-Giordano-2005 |
| CDCA3 | TOP2A | 0.016181 | Co-expression | Dobbin-Giordano-2005 |
| CDK1 | AURKA | 0.049681 | Co-expression | Dobbin-Giordano-2005 |
| CDK1 | AURKB | 0.019206 | Co-expression | Dobbin-Giordano-2005 |
| CDK1 | BUB1B | 0.027109 | Co-expression | Dobbin-Giordano-2005 |
| CDK1 | CCNA2 | 0.027055 | Co-expression | Dobbin-Giordano-2005 |
| CDK1 | CCNB1 | 0.015022 | Co-expression | Dobbin-Giordano-2005 |
| CDK1 | CCNF | 0.035556 | Co-expression | Dobbin-Giordano-2005 |
| CDK1 | CENPE | 0.020655 | Co-expression | Dobbin-Giordano-2005 |
| CDK1 | KIF11 | 0.020885 | Co-expression | Dobbin-Giordano-2005 |
| CDK1 | KIF23 | 0.019927 | Co-expression | Dobbin-Giordano-2005 |
| CDK1 | MKI67 | 0.043139 | Co-expression | Dobbin-Giordano-2005 |
| CDK1 | NEK2 | 0.03335 | Co-expression | Dobbin-Giordano-2005 |
| CDK1 | NUSAP1 | 0.025729 | Co-expression | Dobbin-Giordano-2005 |
| CDK1 | PRC1 | 0.015553 | Co-expression | Dobbin-Giordano-2005 |
| CDK1 | TOP2A | 0.017244 | Co-expression | Dobbin-Giordano-2005 |
| CENPE | BUB1B | 0.027139 | Co-expression | Dobbin-Giordano-2005 |
| CENPE | CCNA2 | 0.028855 | Co-expression | Dobbin-Giordano-2005 |
| CENPE | CCNB1 | 0.016944 | Co-expression | Dobbin-Giordano-2005 |
| CENPE | CCNF | 0.035503 | Co-expression | Dobbin-Giordano-2005 |
| CENPE | CENPF | 0.057748 | Co-expression | Dobbin-Giordano-2005 |
| CENPE | KIF20A | 0.022985 | Co-expression | Dobbin-Giordano-2005 |
| CENPE | KIF23 | 0.021806 | Co-expression | Dobbin-Giordano-2005 |
| CENPE | NEK2 | 0.02993 | Co-expression | Dobbin-Giordano-2005 |
| CENPE | NUSAP1 | 0.028134 | Co-expression | Dobbin-Giordano-2005 |
| CENPE | PRC1 | 0.016547 | Co-expression | Dobbin-Giordano-2005 |
| CENPF | CCNB1 | 0.038321 | Co-expression | Dobbin-Giordano-2005 |
| CENPF | KIF20A | 0.061427 | Co-expression | Dobbin-Giordano-2005 |
| CENPF | NUSAP1 | 0.064374 | Co-expression | Dobbin-Giordano-2005 |
| CENPF | PRC1 | 0.036541 | Co-expression | Dobbin-Giordano-2005 |
| DEPDC1 | AURKB | 0.022356 | Co-expression | Dobbin-Giordano-2005 |
| DEPDC1 | CCNA2 | 0.033236 | Co-expression | Dobbin-Giordano-2005 |
| DEPDC1 | CCNB1 | 0.020369 | Co-expression | Dobbin-Giordano-2005 |
| DEPDC1 | CDCA3 | 0.023011 | Co-expression | Dobbin-Giordano-2005 |
| DEPDC1 | CDK1 | 0.025838 | Co-expression | Dobbin-Giordano-2005 |
| DEPDC1 | CENPE | 0.025023 | Co-expression | Dobbin-Giordano-2005 |
| DEPDC1 | KIF23 | 0.026598 | Co-expression | Dobbin-Giordano-2005 |
| DEPDC1 | NEK2 | 0.040181 | Co-expression | Dobbin-Giordano-2005 |
| DEPDC1 | PRC1 | 0.019674 | Co-expression | Dobbin-Giordano-2005 |
| DEPDC1 | TOP2A | 0.021141 | Co-expression | Dobbin-Giordano-2005 |
| GINS1 | NUSAP1 | 0.033566 | Co-expression | Dobbin-Giordano-2005 |
| HMMR | AURKA | 0.084331 | Co-expression | Dobbin-Giordano-2005 |
| HMMR | BUB1B | 0.04774 | Co-expression | Dobbin-Giordano-2005 |
| HMMR | CCNB1 | 0.022021 | Co-expression | Dobbin-Giordano-2005 |
| HMMR | CDK1 | 0.03024 | Co-expression | Dobbin-Giordano-2005 |
| HMMR | CENPE | 0.03052 | Co-expression | Dobbin-Giordano-2005 |
| HMMR | KIF11 | 0.028717 | Co-expression | Dobbin-Giordano-2005 |
| HMMR | KIF20A | 0.034831 | Co-expression | Dobbin-Giordano-2005 |
| HMMR | MKI67 | 0.070715 | Co-expression | Dobbin-Giordano-2005 |
| HMMR | NUSAP1 | 0.042184 | Co-expression | Dobbin-Giordano-2005 |
| HMMR | PRC1 | 0.021156 | Co-expression | Dobbin-Giordano-2005 |
| HMMR | TOP2A | 0.025634 | Co-expression | Dobbin-Giordano-2005 |
| KIF11 | BUB1B | 0.025514 | Co-expression | Dobbin-Giordano-2005 |
| KIF11 | CCNF | 0.033254 | Co-expression | Dobbin-Giordano-2005 |
| KIF11 | CENPE | 0.01887 | Co-expression | Dobbin-Giordano-2005 |
| KIF11 | GINS1 | 0.026904 | Co-expression | Dobbin-Giordano-2005 |
| KIF11 | MKI67 | 0.042259 | Co-expression | Dobbin-Giordano-2005 |
| KIF11 | NDC80 | 0.035265 | Co-expression | Dobbin-Giordano-2005 |
| KIF11 | NEK2 | 0.028479 | Co-expression | Dobbin-Giordano-2005 |
| KIF11 | NUSAP1 | 0.024492 | Co-expression | Dobbin-Giordano-2005 |
| KIF11 | PRC1 | 0.013814 | Co-expression | Dobbin-Giordano-2005 |
| KIF11 | TOP2A | 0.016402 | Co-expression | Dobbin-Giordano-2005 |
| KIF23 | CCNB1 | 0.016176 | Co-expression | Dobbin-Giordano-2005 |
| KIF23 | NEK2 | 0.030295 | Co-expression | Dobbin-Giordano-2005 |
| KIF23 | PRC1 | 0.015746 | Co-expression | Dobbin-Giordano-2005 |
| KIF4A | CCNA2 | 0.018308 | Co-expression | Dobbin-Giordano-2005 |
| KIF4A | CCNF | 0.025981 | Co-expression | Dobbin-Giordano-2005 |
| KIF4A | CDK1 | 0.015498 | Co-expression | Dobbin-Giordano-2005 |
| KIF4A | GINS1 | 0.019104 | Co-expression | Dobbin-Giordano-2005 |
| KIF4A | KIF11 | 0.015254 | Co-expression | Dobbin-Giordano-2005 |
| KIF4A | NDC80 | 0.023623 | Co-expression | Dobbin-Giordano-2005 |
| KIF4A | NUSAP1 | 0.018027 | Co-expression | Dobbin-Giordano-2005 |
| KIF4A | PRC1 | 0.010707 | Co-expression | Dobbin-Giordano-2005 |
| KIF4A | ZWINT | 0.01425 | Co-expression | Dobbin-Giordano-2005 |
| MKI67 | BUB1B | 0.055814 | Co-expression | Dobbin-Giordano-2005 |
| MKI67 | CCNB1 | 0.029382 | Co-expression | Dobbin-Giordano-2005 |
| MKI67 | CENPE | 0.042629 | Co-expression | Dobbin-Giordano-2005 |
| MKI67 | KIF20A | 0.042592 | Co-expression | Dobbin-Giordano-2005 |
| MKI67 | KIF23 | 0.038138 | Co-expression | Dobbin-Giordano-2005 |
| MKI67 | NUSAP1 | 0.053403 | Co-expression | Dobbin-Giordano-2005 |
| MKI67 | PRC1 | 0.029971 | Co-expression | Dobbin-Giordano-2005 |
| NDC80 | BUB1B | 0.050032 | Co-expression | Dobbin-Giordano-2005 |
| NDC80 | CENPE | 0.035029 | Co-expression | Dobbin-Giordano-2005 |
| NDC80 | CENPF | 0.090486 | Co-expression | Dobbin-Giordano-2005 |
| NDC80 | GINS1 | 0.04501 | Co-expression | Dobbin-Giordano-2005 |
| NDC80 | KIF20A | 0.03413 | Co-expression | Dobbin-Giordano-2005 |
| NDC80 | NUSAP1 | 0.047052 | Co-expression | Dobbin-Giordano-2005 |
| NDC80 | PRC1 | 0.022826 | Co-expression | Dobbin-Giordano-2005 |
| NUSAP1 | KIF20A | 0.026655 | Co-expression | Dobbin-Giordano-2005 |
| PRC1 | KIF20A | 0.013992 | Co-expression | Dobbin-Giordano-2005 |
| PRC1 | NEK2 | 0.023824 | Co-expression | Dobbin-Giordano-2005 |
| PRC1 | NUSAP1 | 0.01848 | Co-expression | Dobbin-Giordano-2005 |
| SMC4 | CCNA2 | 0.073852 | Co-expression | Dobbin-Giordano-2005 |
| SMC4 | CCNB1 | 0.042681 | Co-expression | Dobbin-Giordano-2005 |
| SMC4 | CDK1 | 0.05391 | Co-expression | Dobbin-Giordano-2005 |
| SMC4 | CENPE | 0.060583 | Co-expression | Dobbin-Giordano-2005 |
| SMC4 | KIF11 | 0.04979 | Co-expression | Dobbin-Giordano-2005 |
| SMC4 | KIF20A | 0.057992 | Co-expression | Dobbin-Giordano-2005 |
| SMC4 | KIF23 | 0.055774 | Co-expression | Dobbin-Giordano-2005 |
| SMC4 | NDC80 | 0.094778 | Co-expression | Dobbin-Giordano-2005 |
| SMC4 | NEK2 | 0.084224 | Co-expression | Dobbin-Giordano-2005 |
| SMC4 | NUSAP1 | 0.072911 | Co-expression | Dobbin-Giordano-2005 |
| SMC4 | PRC1 | 0.04136 | Co-expression | Dobbin-Giordano-2005 |
| SMC4 | TOP2A | 0.050053 | Co-expression | Dobbin-Giordano-2005 |
| SMC4 | ZWINT | 0.047164 | Co-expression | Dobbin-Giordano-2005 |
| TOP2A | AURKA | 0.046731 | Co-expression | Dobbin-Giordano-2005 |
| TOP2A | CCNA2 | 0.022875 | Co-expression | Dobbin-Giordano-2005 |
| TOP2A | CCNB1 | 0.013418 | Co-expression | Dobbin-Giordano-2005 |
| TOP2A | CENPE | 0.020609 | Co-expression | Dobbin-Giordano-2005 |
| TOP2A | CENPF | 0.05045 | Co-expression | Dobbin-Giordano-2005 |
| TOP2A | KIF20A | 0.020108 | Co-expression | Dobbin-Giordano-2005 |
| TOP2A | KIF23 | 0.017915 | Co-expression | Dobbin-Giordano-2005 |
| TOP2A | MKI67 | 0.037649 | Co-expression | Dobbin-Giordano-2005 |
| TOP2A | NDC80 | 0.030099 | Co-expression | Dobbin-Giordano-2005 |
| TOP2A | NEK2 | 0.025534 | Co-expression | Dobbin-Giordano-2005 |
| TOP2A | NUSAP1 | 0.022693 | Co-expression | Dobbin-Giordano-2005 |
| TOP2A | PRC1 | 0.013346 | Co-expression | Dobbin-Giordano-2005 |
| ZWINT | BUB1B | 0.022694 | Co-expression | Dobbin-Giordano-2005 |
| ZWINT | CCNA2 | 0.022387 | Co-expression | Dobbin-Giordano-2005 |
| ZWINT | CCNF | 0.029777 | Co-expression | Dobbin-Giordano-2005 |
| ZWINT | CDK1 | 0.018939 | Co-expression | Dobbin-Giordano-2005 |
| ZWINT | CENPF | 0.043518 | Co-expression | Dobbin-Giordano-2005 |
| ZWINT | GINS1 | 0.026384 | Co-expression | Dobbin-Giordano-2005 |
| ZWINT | HMMR | 0.02544 | Co-expression | Dobbin-Giordano-2005 |
| ZWINT | KIF11 | 0.01834 | Co-expression | Dobbin-Giordano-2005 |
| ZWINT | MKI67 | 0.035857 | Co-expression | Dobbin-Giordano-2005 |
| ZWINT | NDC80 | 0.028242 | Co-expression | Dobbin-Giordano-2005 |
| ZWINT | NEK2 | 0.025491 | Co-expression | Dobbin-Giordano-2005 |
| ZWINT | NUSAP1 | 0.023102 | Co-expression | Dobbin-Giordano-2005 |
| ZWINT | PRC1 | 0.012994 | Co-expression | Dobbin-Giordano-2005 |
| ZWINT | TOP2A | 0.014734 | Co-expression | Dobbin-Giordano-2005 |
| ASPM | AURKA | 0.218232 | Co-expression | Innocenti-Brown-2011 |
| ASPM | AURKB | 0.210159 | Co-expression | Innocenti-Brown-2011 |
| ASPM | BUB1B | 0.143029 | Co-expression | Innocenti-Brown-2011 |
| ASPM | CCNA2 | 0.146583 | Co-expression | Innocenti-Brown-2011 |
| ASPM | CCNB1 | 0.150927 | Co-expression | Innocenti-Brown-2011 |
| ASPM | CCNF | 0.198782 | Co-expression | Innocenti-Brown-2011 |
| ASPM | CDC25C | 0.204665 | Co-expression | Innocenti-Brown-2011 |
| ASPM | CDCA3 | 0.241758 | Co-expression | Innocenti-Brown-2011 |
| ASPM | CDK1 | 0.153379 | Co-expression | Innocenti-Brown-2011 |
| ASPM | CENPE | 0.166853 | Co-expression | Innocenti-Brown-2011 |
| ASPM | CENPF | 0.204766 | Co-expression | Innocenti-Brown-2011 |
| ASPM | DEPDC1 | 0.271721 | Co-expression | Innocenti-Brown-2011 |
| ASPM | GINS1 | 0.17748 | Co-expression | Innocenti-Brown-2011 |
| ASPM | HMMR | 0.164202 | Co-expression | Innocenti-Brown-2011 |
| ASPM | KIF11 | 0.248752 | Co-expression | Innocenti-Brown-2011 |
| ASPM | KIF20A | 0.202885 | Co-expression | Innocenti-Brown-2011 |
| ASPM | KIF23 | 0.160378 | Co-expression | Innocenti-Brown-2011 |
| ASPM | KIF4A | 0.145119 | Co-expression | Innocenti-Brown-2011 |
| ASPM | MKI67 | 0.24647 | Co-expression | Innocenti-Brown-2011 |
| ASPM | NDC80 | 0.192436 | Co-expression | Innocenti-Brown-2011 |
| ASPM | NEK2 | 0.208979 | Co-expression | Innocenti-Brown-2011 |
| ASPM | NUSAP1 | 0.22229 | Co-expression | Innocenti-Brown-2011 |
| ASPM | PRC1 | 0.146004 | Co-expression | Innocenti-Brown-2011 |
| ASPM | SMC4 | 0.17331 | Co-expression | Innocenti-Brown-2011 |
| ASPM | TOP2A | 0.140776 | Co-expression | Innocenti-Brown-2011 |
| ASPM | ZWINT | 0.121441 | Co-expression | Innocenti-Brown-2011 |
| AURKA | BUB1B | 0.199867 | Co-expression | Innocenti-Brown-2011 |
| AURKA | CCNA2 | 0.242672 | Co-expression | Innocenti-Brown-2011 |
| AURKA | CCNB1 | 0.269818 | Co-expression | Innocenti-Brown-2011 |
| AURKA | CENPE | 0.240392 | Co-expression | Innocenti-Brown-2011 |
| AURKA | CENPF | 0.307126 | Co-expression | Innocenti-Brown-2011 |
| AURKA | KIF20A | 0.330604 | Co-expression | Innocenti-Brown-2011 |
| AURKA | NEK2 | 0.343775 | Co-expression | Innocenti-Brown-2011 |
| AURKA | PRC1 | 0.223685 | Co-expression | Innocenti-Brown-2011 |
| AURKB | BUB1B | 0.242771 | Co-expression | Innocenti-Brown-2011 |
| AURKB | CCNA2 | 0.243395 | Co-expression | Innocenti-Brown-2011 |
| AURKB | CCNB1 | 0.209558 | Co-expression | Innocenti-Brown-2011 |
| AURKB | CCNF | 0.36004 | Co-expression | Innocenti-Brown-2011 |
| AURKB | CENPE | 0.253271 | Co-expression | Innocenti-Brown-2011 |
| AURKB | CENPF | 0.299712 | Co-expression | Innocenti-Brown-2011 |
| AURKB | GINS1 | 0.348493 | Co-expression | Innocenti-Brown-2011 |
| AURKB | KIF20A | 0.31572 | Co-expression | Innocenti-Brown-2011 |
| AURKB | KIF23 | 0.22739 | Co-expression | Innocenti-Brown-2011 |
| AURKB | MKI67 | 0.382211 | Co-expression | Innocenti-Brown-2011 |
| AURKB | NDC80 | 0.312463 | Co-expression | Innocenti-Brown-2011 |
| AURKB | NEK2 | 0.345184 | Co-expression | Innocenti-Brown-2011 |
| AURKB | NUSAP1 | 0.397716 | Co-expression | Innocenti-Brown-2011 |
| AURKB | PRC1 | 0.195958 | Co-expression | Innocenti-Brown-2011 |
| AURKB | TOP2A | 0.206055 | Co-expression | Innocenti-Brown-2011 |
| BUB1B | KIF20A | 0.224595 | Co-expression | Innocenti-Brown-2011 |
| BUB1B | NEK2 | 0.202315 | Co-expression | Innocenti-Brown-2011 |
| BUB1B | NUSAP1 | 0.220973 | Co-expression | Innocenti-Brown-2011 |
| BUB1B | PRC1 | 0.144544 | Co-expression | Innocenti-Brown-2011 |
| CCNA2 | BUB1B | 0.152838 | Co-expression | Innocenti-Brown-2011 |
| CCNA2 | CCNB1 | 0.153996 | Co-expression | Innocenti-Brown-2011 |
| CCNA2 | CCNF | 0.230287 | Co-expression | Innocenti-Brown-2011 |
| CCNA2 | CENPF | 0.203957 | Co-expression | Innocenti-Brown-2011 |
| CCNA2 | GINS1 | 0.209929 | Co-expression | Innocenti-Brown-2011 |
| CCNA2 | KIF20A | 0.226113 | Co-expression | Innocenti-Brown-2011 |
| CCNA2 | KIF23 | 0.161859 | Co-expression | Innocenti-Brown-2011 |
| CCNA2 | NEK2 | 0.232077 | Co-expression | Innocenti-Brown-2011 |
| CCNA2 | NUSAP1 | 0.241019 | Co-expression | Innocenti-Brown-2011 |
| CCNA2 | PRC1 | 0.142263 | Co-expression | Innocenti-Brown-2011 |
| CCNB1 | BUB1B | 0.1459 | Co-expression | Innocenti-Brown-2011 |
| CCNB1 | KIF20A | 0.22989 | Co-expression | Innocenti-Brown-2011 |
| CCNB1 | NEK2 | 0.215102 | Co-expression | Innocenti-Brown-2011 |
| CCNB1 | NUSAP1 | 0.234252 | Co-expression | Innocenti-Brown-2011 |
| CCNB1 | PRC1 | 0.152978 | Co-expression | Innocenti-Brown-2011 |
| CCNF | BUB1B | 0.178146 | Co-expression | Innocenti-Brown-2011 |
| CCNF | CCNB1 | 0.17844 | Co-expression | Innocenti-Brown-2011 |
| CCNF | CENPF | 0.293663 | Co-expression | Innocenti-Brown-2011 |
| CCNF | KIF20A | 0.318994 | Co-expression | Innocenti-Brown-2011 |
| CCNF | NEK2 | 0.343628 | Co-expression | Innocenti-Brown-2011 |
| CDC25C | AURKA | 0.30832 | Co-expression | Innocenti-Brown-2011 |
| CDC25C | AURKB | 0.356012 | Co-expression | Innocenti-Brown-2011 |
| CDC25C | BUB1B | 0.204733 | Co-expression | Innocenti-Brown-2011 |
| CDC25C | CCNA2 | 0.217856 | Co-expression | Innocenti-Brown-2011 |
| CDC25C | CCNB1 | 0.198502 | Co-expression | Innocenti-Brown-2011 |
| CDC25C | CCNF | 0.361678 | Co-expression | Innocenti-Brown-2011 |
| CDC25C | CDCA3 | 0.336792 | Co-expression | Innocenti-Brown-2011 |
| CDC25C | CDK1 | 0.20475 | Co-expression | Innocenti-Brown-2011 |
| CDC25C | CENPE | 0.240718 | Co-expression | Innocenti-Brown-2011 |
| CDC25C | CENPF | 0.26702 | Co-expression | Innocenti-Brown-2011 |
| CDC25C | DEPDC1 | 0.432961 | Co-expression | Innocenti-Brown-2011 |
| CDC25C | HMMR | 0.218193 | Co-expression | Innocenti-Brown-2011 |
| CDC25C | KIF20A | 0.304858 | Co-expression | Innocenti-Brown-2011 |
| CDC25C | KIF23 | 0.206591 | Co-expression | Innocenti-Brown-2011 |
| CDC25C | KIF4A | 0.229016 | Co-expression | Innocenti-Brown-2011 |
| CDC25C | MKI67 | 0.379793 | Co-expression | Innocenti-Brown-2011 |
| CDC25C | NDC80 | 0.293618 | Co-expression | Innocenti-Brown-2011 |
| CDC25C | NEK2 | 0.314505 | Co-expression | Innocenti-Brown-2011 |
| CDC25C | NUSAP1 | 0.343908 | Co-expression | Innocenti-Brown-2011 |
| CDC25C | PRC1 | 0.182874 | Co-expression | Innocenti-Brown-2011 |
| CDC25C | TOP2A | 0.187927 | Co-expression | Innocenti-Brown-2011 |
| CDC25C | ZWINT | 0.149313 | Co-expression | Innocenti-Brown-2011 |
| CDCA3 | AURKA | 0.38645 | Co-expression | Innocenti-Brown-2011 |
| CDCA3 | AURKB | 0.384868 | Co-expression | Innocenti-Brown-2011 |
| CDCA3 | BUB1B | 0.249093 | Co-expression | Innocenti-Brown-2011 |
| CDCA3 | CCNA2 | 0.254739 | Co-expression | Innocenti-Brown-2011 |
| CDCA3 | CCNB1 | 0.241465 | Co-expression | Innocenti-Brown-2011 |
| CDCA3 | CCNF | 0.363179 | Co-expression | Innocenti-Brown-2011 |
| CDCA3 | CDK1 | 0.238377 | Co-expression | Innocenti-Brown-2011 |
| CDCA3 | CENPE | 0.269072 | Co-expression | Innocenti-Brown-2011 |
| CDCA3 | CENPF | 0.323572 | Co-expression | Innocenti-Brown-2011 |
| CDCA3 | KIF11 | 0.41088 | Co-expression | Innocenti-Brown-2011 |
| CDCA3 | KIF20A | 0.362928 | Co-expression | Innocenti-Brown-2011 |
| CDCA3 | KIF23 | 0.261921 | Co-expression | Innocenti-Brown-2011 |
| CDCA3 | MKI67 | 0.415452 | Co-expression | Innocenti-Brown-2011 |
| CDCA3 | NDC80 | 0.3004 | Co-expression | Innocenti-Brown-2011 |
| CDCA3 | NEK2 | 0.354011 | Co-expression | Innocenti-Brown-2011 |
| CDCA3 | NUSAP1 | 0.344023 | Co-expression | Innocenti-Brown-2011 |
| CDCA3 | PRC1 | 0.227252 | Co-expression | Innocenti-Brown-2011 |
| CDCA3 | TOP2A | 0.21869 | Co-expression | Innocenti-Brown-2011 |
| CDK1 | AURKB | 0.231256 | Co-expression | Innocenti-Brown-2011 |
| CDK1 | BUB1B | 0.162213 | Co-expression | Innocenti-Brown-2011 |
| CDK1 | CCNA2 | 0.158847 | Co-expression | Innocenti-Brown-2011 |
| CDK1 | CCNB1 | 0.159725 | Co-expression | Innocenti-Brown-2011 |
| CDK1 | CCNF | 0.197692 | Co-expression | Innocenti-Brown-2011 |
| CDK1 | CENPE | 0.183103 | Co-expression | Innocenti-Brown-2011 |
| CDK1 | CENPF | 0.215046 | Co-expression | Innocenti-Brown-2011 |
| CDK1 | KIF11 | 0.284818 | Co-expression | Innocenti-Brown-2011 |
| CDK1 | KIF20A | 0.224955 | Co-expression | Innocenti-Brown-2011 |
| CDK1 | KIF23 | 0.178325 | Co-expression | Innocenti-Brown-2011 |
| CDK1 | MKI67 | 0.261656 | Co-expression | Innocenti-Brown-2011 |
| CDK1 | NDC80 | 0.213215 | Co-expression | Innocenti-Brown-2011 |
| CDK1 | NEK2 | 0.211046 | Co-expression | Innocenti-Brown-2011 |
| CDK1 | NUSAP1 | 0.235528 | Co-expression | Innocenti-Brown-2011 |
| CDK1 | PRC1 | 0.159089 | Co-expression | Innocenti-Brown-2011 |
| CDK1 | TOP2A | 0.162059 | Co-expression | Innocenti-Brown-2011 |
| CENPE | BUB1B | 0.171733 | Co-expression | Innocenti-Brown-2011 |
| CENPE | CCNA2 | 0.171992 | Co-expression | Innocenti-Brown-2011 |
| CENPE | CCNB1 | 0.172133 | Co-expression | Innocenti-Brown-2011 |
| CENPE | CCNF | 0.213989 | Co-expression | Innocenti-Brown-2011 |
| CENPE | CENPF | 0.23235 | Co-expression | Innocenti-Brown-2011 |
| CENPE | KIF20A | 0.241141 | Co-expression | Innocenti-Brown-2011 |
| CENPE | KIF23 | 0.187482 | Co-expression | Innocenti-Brown-2011 |
| CENPE | NEK2 | 0.231406 | Co-expression | Innocenti-Brown-2011 |
| CENPE | NUSAP1 | 0.27389 | Co-expression | Innocenti-Brown-2011 |
| CENPE | PRC1 | 0.163534 | Co-expression | Innocenti-Brown-2011 |
| CENPF | BUB1B | 0.20191 | Co-expression | Innocenti-Brown-2011 |
| CENPF | CCNB1 | 0.209271 | Co-expression | Innocenti-Brown-2011 |
| CENPF | KIF20A | 0.268705 | Co-expression | Innocenti-Brown-2011 |
| CENPF | NEK2 | 0.313959 | Co-expression | Innocenti-Brown-2011 |
| CENPF | PRC1 | 0.202025 | Co-expression | Innocenti-Brown-2011 |
| DEPDC1 | AURKB | 0.399569 | Co-expression | Innocenti-Brown-2011 |
| DEPDC1 | BUB1B | 0.239392 | Co-expression | Innocenti-Brown-2011 |
| DEPDC1 | CCNA2 | 0.279537 | Co-expression | Innocenti-Brown-2011 |
| DEPDC1 | CCNB1 | 0.253958 | Co-expression | Innocenti-Brown-2011 |
| DEPDC1 | CDCA3 | 0.43448 | Co-expression | Innocenti-Brown-2011 |
| DEPDC1 | CDK1 | 0.261399 | Co-expression | Innocenti-Brown-2011 |
| DEPDC1 | CENPE | 0.293379 | Co-expression | Innocenti-Brown-2011 |
| DEPDC1 | CENPF | 0.373352 | Co-expression | Innocenti-Brown-2011 |
| DEPDC1 | HMMR | 0.267956 | Co-expression | Innocenti-Brown-2011 |
| DEPDC1 | KIF20A | 0.377576 | Co-expression | Innocenti-Brown-2011 |
| DEPDC1 | KIF23 | 0.272421 | Co-expression | Innocenti-Brown-2011 |
| DEPDC1 | KIF4A | 0.288373 | Co-expression | Innocenti-Brown-2011 |
| DEPDC1 | NDC80 | 0.353071 | Co-expression | Innocenti-Brown-2011 |
| DEPDC1 | NEK2 | 0.425028 | Co-expression | Innocenti-Brown-2011 |
| DEPDC1 | PRC1 | 0.228584 | Co-expression | Innocenti-Brown-2011 |
| DEPDC1 | TOP2A | 0.254748 | Co-expression | Innocenti-Brown-2011 |
| GINS1 | NEK2 | 0.307611 | Co-expression | Innocenti-Brown-2011 |
| HMMR | AURKA | 0.244799 | Co-expression | Innocenti-Brown-2011 |
| HMMR | AURKB | 0.230108 | Co-expression | Innocenti-Brown-2011 |
| HMMR | BUB1B | 0.158375 | Co-expression | Innocenti-Brown-2011 |
| HMMR | CCNA2 | 0.162512 | Co-expression | Innocenti-Brown-2011 |
| HMMR | CCNB1 | 0.165695 | Co-expression | Innocenti-Brown-2011 |
| HMMR | CCNF | 0.222049 | Co-expression | Innocenti-Brown-2011 |
| HMMR | CDCA3 | 0.264717 | Co-expression | Innocenti-Brown-2011 |
| HMMR | CDK1 | 0.171304 | Co-expression | Innocenti-Brown-2011 |
| HMMR | CENPE | 0.183635 | Co-expression | Innocenti-Brown-2011 |
| HMMR | CENPF | 0.219978 | Co-expression | Innocenti-Brown-2011 |
| HMMR | GINS1 | 0.199869 | Co-expression | Innocenti-Brown-2011 |
| HMMR | KIF11 | 0.272762 | Co-expression | Innocenti-Brown-2011 |
| HMMR | KIF20A | 0.226934 | Co-expression | Innocenti-Brown-2011 |
| HMMR | KIF23 | 0.184173 | Co-expression | Innocenti-Brown-2011 |
| HMMR | MKI67 | 0.267745 | Co-expression | Innocenti-Brown-2011 |
| HMMR | NDC80 | 0.211128 | Co-expression | Innocenti-Brown-2011 |
| HMMR | NEK2 | 0.225699 | Co-expression | Innocenti-Brown-2011 |
| HMMR | NUSAP1 | 0.224488 | Co-expression | Innocenti-Brown-2011 |
| HMMR | PRC1 | 0.166008 | Co-expression | Innocenti-Brown-2011 |
| HMMR | TOP2A | 0.154579 | Co-expression | Innocenti-Brown-2011 |
| KIF11 | BUB1B | 0.269916 | Co-expression | Innocenti-Brown-2011 |
| KIF11 | CCNA2 | 0.280258 | Co-expression | Innocenti-Brown-2011 |
| KIF11 | CCNB1 | 0.264463 | Co-expression | Innocenti-Brown-2011 |
| KIF11 | CENPE | 0.299242 | Co-expression | Innocenti-Brown-2011 |
| KIF11 | CENPF | 0.388663 | Co-expression | Innocenti-Brown-2011 |
| KIF11 | KIF20A | 0.39704 | Co-expression | Innocenti-Brown-2011 |
| KIF11 | KIF23 | 0.290523 | Co-expression | Innocenti-Brown-2011 |
| KIF11 | NDC80 | 0.340049 | Co-expression | Innocenti-Brown-2011 |
| KIF11 | NEK2 | 0.411009 | Co-expression | Innocenti-Brown-2011 |
| KIF11 | PRC1 | 0.249172 | Co-expression | Innocenti-Brown-2011 |
| KIF11 | TOP2A | 0.263598 | Co-expression | Innocenti-Brown-2011 |
| KIF20A | NEK2 | 0.305455 | Co-expression | Innocenti-Brown-2011 |
| KIF23 | BUB1B | 0.174148 | Co-expression | Innocenti-Brown-2011 |
| KIF23 | CCNB1 | 0.164728 | Co-expression | Innocenti-Brown-2011 |
| KIF23 | CENPF | 0.219235 | Co-expression | Innocenti-Brown-2011 |
| KIF23 | KIF20A | 0.254631 | Co-expression | Innocenti-Brown-2011 |
| KIF23 | NEK2 | 0.218741 | Co-expression | Innocenti-Brown-2011 |
| KIF23 | NUSAP1 | 0.217668 | Co-expression | Innocenti-Brown-2011 |
| KIF23 | PRC1 | 0.167391 | Co-expression | Innocenti-Brown-2011 |
| KIF4A | AURKA | 0.205406 | Co-expression | Innocenti-Brown-2011 |
| KIF4A | AURKB | 0.244257 | Co-expression | Innocenti-Brown-2011 |
| KIF4A | BUB1B | 0.151891 | Co-expression | Innocenti-Brown-2011 |
| KIF4A | CCNA2 | 0.162786 | Co-expression | Innocenti-Brown-2011 |
| KIF4A | CCNB1 | 0.146672 | Co-expression | Innocenti-Brown-2011 |
| KIF4A | CCNF | 0.239574 | Co-expression | Innocenti-Brown-2011 |
| KIF4A | CDCA3 | 0.23783 | Co-expression | Innocenti-Brown-2011 |
| KIF4A | CDK1 | 0.162394 | Co-expression | Innocenti-Brown-2011 |
| KIF4A | CENPE | 0.171539 | Co-expression | Innocenti-Brown-2011 |
| KIF4A | CENPF | 0.20308 | Co-expression | Innocenti-Brown-2011 |
| KIF4A | GINS1 | 0.188182 | Co-expression | Innocenti-Brown-2011 |
| KIF4A | HMMR | 0.156895 | Co-expression | Innocenti-Brown-2011 |
| KIF4A | KIF11 | 0.275015 | Co-expression | Innocenti-Brown-2011 |
| KIF4A | KIF20A | 0.221394 | Co-expression | Innocenti-Brown-2011 |
| KIF4A | KIF23 | 0.160349 | Co-expression | Innocenti-Brown-2011 |
| KIF4A | MKI67 | 0.262465 | Co-expression | Innocenti-Brown-2011 |
| KIF4A | NDC80 | 0.225192 | Co-expression | Innocenti-Brown-2011 |
| KIF4A | NEK2 | 0.221889 | Co-expression | Innocenti-Brown-2011 |
| KIF4A | NUSAP1 | 0.226441 | Co-expression | Innocenti-Brown-2011 |
| KIF4A | PRC1 | 0.14155 | Co-expression | Innocenti-Brown-2011 |
| KIF4A | TOP2A | 0.146119 | Co-expression | Innocenti-Brown-2011 |
| KIF4A | ZWINT | 0.118792 | Co-expression | Innocenti-Brown-2011 |
| MKI67 | BUB1B | 0.259987 | Co-expression | Innocenti-Brown-2011 |
| MKI67 | CCNA2 | 0.268862 | Co-expression | Innocenti-Brown-2011 |
| MKI67 | CCNB1 | 0.246544 | Co-expression | Innocenti-Brown-2011 |
| MKI67 | CCNF | 0.47927 | Co-expression | Innocenti-Brown-2011 |
| MKI67 | CENPE | 0.288894 | Co-expression | Innocenti-Brown-2011 |
| MKI67 | CENPF | 0.339647 | Co-expression | Innocenti-Brown-2011 |
| MKI67 | KIF20A | 0.379314 | Co-expression | Innocenti-Brown-2011 |
| MKI67 | KIF23 | 0.273825 | Co-expression | Innocenti-Brown-2011 |
| MKI67 | NDC80 | 0.321602 | Co-expression | Innocenti-Brown-2011 |
| MKI67 | NEK2 | 0.370913 | Co-expression | Innocenti-Brown-2011 |
| MKI67 | PRC1 | 0.236483 | Co-expression | Innocenti-Brown-2011 |
| NDC80 | BUB1B | 0.193712 | Co-expression | Innocenti-Brown-2011 |
| NDC80 | CCNA2 | 0.200004 | Co-expression | Innocenti-Brown-2011 |
| NDC80 | CCNB1 | 0.192183 | Co-expression | Innocenti-Brown-2011 |
| NDC80 | CCNF | 0.266115 | Co-expression | Innocenti-Brown-2011 |
| NDC80 | CENPE | 0.223832 | Co-expression | Innocenti-Brown-2011 |
| NDC80 | CENPF | 0.278972 | Co-expression | Innocenti-Brown-2011 |
| NDC80 | KIF20A | 0.27102 | Co-expression | Innocenti-Brown-2011 |
| NDC80 | KIF23 | 0.204608 | Co-expression | Innocenti-Brown-2011 |
| NDC80 | NEK2 | 0.281581 | Co-expression | Innocenti-Brown-2011 |
| NDC80 | NUSAP1 | 0.289891 | Co-expression | Innocenti-Brown-2011 |
| NDC80 | PRC1 | 0.190081 | Co-expression | Innocenti-Brown-2011 |
| NUSAP1 | KIF20A | 0.332943 | Co-expression | Innocenti-Brown-2011 |
| PRC1 | GINS1 | 0.180053 | Co-expression | Innocenti-Brown-2011 |
| PRC1 | KIF20A | 0.210061 | Co-expression | Innocenti-Brown-2011 |
| PRC1 | NEK2 | 0.192928 | Co-expression | Innocenti-Brown-2011 |
| PRC1 | NUSAP1 | 0.197873 | Co-expression | Innocenti-Brown-2011 |
| SMC4 | CCNA2 | 0.201592 | Co-expression | Innocenti-Brown-2011 |
| SMC4 | CCNB1 | 0.224004 | Co-expression | Innocenti-Brown-2011 |
| SMC4 | CENPE | 0.211257 | Co-expression | Innocenti-Brown-2011 |
| SMC4 | HMMR | 0.194516 | Co-expression | Innocenti-Brown-2011 |
| SMC4 | KIF20A | 0.286807 | Co-expression | Innocenti-Brown-2011 |
| SMC4 | NUSAP1 | 0.581737 | Co-expression | Innocenti-Brown-2011 |
| SMC4 | PRC1 | 0.168386 | Co-expression | Innocenti-Brown-2011 |
| SMC4 | TOP2A | 0.178638 | Co-expression | Innocenti-Brown-2011 |
| TOP2A | AURKA | 0.197476 | Co-expression | Innocenti-Brown-2011 |
| TOP2A | BUB1B | 0.144643 | Co-expression | Innocenti-Brown-2011 |
| TOP2A | CCNA2 | 0.144485 | Co-expression | Innocenti-Brown-2011 |
| TOP2A | CCNB1 | 0.150785 | Co-expression | Innocenti-Brown-2011 |
| TOP2A | CCNF | 0.188044 | Co-expression | Innocenti-Brown-2011 |
| TOP2A | CENPE | 0.16326 | Co-expression | Innocenti-Brown-2011 |
| TOP2A | CENPF | 0.192905 | Co-expression | Innocenti-Brown-2011 |
| TOP2A | GINS1 | 0.164792 | Co-expression | Innocenti-Brown-2011 |
| TOP2A | KIF20A | 0.208901 | Co-expression | Innocenti-Brown-2011 |
| TOP2A | KIF23 | 0.157037 | Co-expression | Innocenti-Brown-2011 |
| TOP2A | MKI67 | 0.232445 | Co-expression | Innocenti-Brown-2011 |
| TOP2A | NDC80 | 0.191907 | Co-expression | Innocenti-Brown-2011 |
| TOP2A | NEK2 | 0.194498 | Co-expression | Innocenti-Brown-2011 |
| TOP2A | NUSAP1 | 0.219535 | Co-expression | Innocenti-Brown-2011 |
| TOP2A | PRC1 | 0.143311 | Co-expression | Innocenti-Brown-2011 |
| ZWINT | AURKA | 0.171634 | Co-expression | Innocenti-Brown-2011 |
| ZWINT | AURKB | 0.162081 | Co-expression | Innocenti-Brown-2011 |
| ZWINT | BUB1B | 0.124141 | Co-expression | Innocenti-Brown-2011 |
| ZWINT | CCNA2 | 0.11934 | Co-expression | Innocenti-Brown-2011 |
| ZWINT | CCNB1 | 0.127981 | Co-expression | Innocenti-Brown-2011 |
| ZWINT | CDCA3 | 0.18071 | Co-expression | Innocenti-Brown-2011 |
| ZWINT | CDK1 | 0.138591 | Co-expression | Innocenti-Brown-2011 |
| ZWINT | CENPE | 0.136923 | Co-expression | Innocenti-Brown-2011 |
| ZWINT | CENPF | 0.166071 | Co-expression | Innocenti-Brown-2011 |
| ZWINT | GINS1 | 0.182338 | Co-expression | Innocenti-Brown-2011 |
| ZWINT | HMMR | 0.132222 | Co-expression | Innocenti-Brown-2011 |
| ZWINT | KIF11 | 0.210201 | Co-expression | Innocenti-Brown-2011 |
| ZWINT | KIF20A | 0.168127 | Co-expression | Innocenti-Brown-2011 |
| ZWINT | KIF23 | 0.134692 | Co-expression | Innocenti-Brown-2011 |
| ZWINT | NDC80 | 0.164615 | Co-expression | Innocenti-Brown-2011 |
| ZWINT | NEK2 | 0.16292 | Co-expression | Innocenti-Brown-2011 |
| ZWINT | NUSAP1 | 0.16967 | Co-expression | Innocenti-Brown-2011 |
| ZWINT | PRC1 | 0.128947 | Co-expression | Innocenti-Brown-2011 |
| ZWINT | TOP2A | 0.121424 | Co-expression | Innocenti-Brown-2011 |
| ASPM | CENPF | 0.028872 | Co-expression | Mallon-McKay-2013 |
| ASPM | KIF4A | 0.020256 | Co-expression | Mallon-McKay-2013 |
| ASPM | SMC4 | 0.019341 | Co-expression | Mallon-McKay-2013 |
| AURKA | CCNA2 | 0.008926 | Co-expression | Mallon-McKay-2013 |
| AURKA | CCNB1 | 0.006931 | Co-expression | Mallon-McKay-2013 |
| AURKA | KIF23 | 0.006353 | Co-expression | Mallon-McKay-2013 |
| AURKB | CCNA2 | 0.012629 | Co-expression | Mallon-McKay-2013 |
| AURKB | CCNB1 | 0.009789 | Co-expression | Mallon-McKay-2013 |
| AURKB | KIF23 | 0.008687 | Co-expression | Mallon-McKay-2013 |
| CCNA2 | CCNB1 | 0.006022 | Co-expression | Mallon-McKay-2013 |
| CCNA2 | KIF23 | 0.005373 | Co-expression | Mallon-McKay-2013 |
| CDC25C | CENPE | 0.020224 | Co-expression | Mallon-McKay-2013 |
| CDCA3 | AURKA | 0.013504 | Co-expression | Mallon-McKay-2013 |
| CDK1 | CENPE | 0.01978 | Co-expression | Mallon-McKay-2013 |
| CENPE | CENPF | 0.016928 | Co-expression | Mallon-McKay-2013 |
| CENPE | KIF20A | 0.012516 | Co-expression | Mallon-McKay-2013 |
| CENPE | KIF23 | 0.007469 | Co-expression | Mallon-McKay-2013 |
| CENPF | KIF20A | 0.013502 | Co-expression | Mallon-McKay-2013 |
| DEPDC1 | CDK1 | 0.033606 | Co-expression | Mallon-McKay-2013 |
| DEPDC1 | CENPE | 0.027964 | Co-expression | Mallon-McKay-2013 |
| DEPDC1 | CENPF | 0.030101 | Co-expression | Mallon-McKay-2013 |
| DEPDC1 | KIF20A | 0.02136 | Co-expression | Mallon-McKay-2013 |
| DEPDC1 | KIF23 | 0.012606 | Co-expression | Mallon-McKay-2013 |
| DEPDC1 | KIF4A | 0.022001 | Co-expression | Mallon-McKay-2013 |
| DEPDC1 | TOP2A | 0.019537 | Co-expression | Mallon-McKay-2013 |
| GINS1 | NUSAP1 | 0.014225 | Co-expression | Mallon-McKay-2013 |
| HMMR | CCNA2 | 0.010314 | Co-expression | Mallon-McKay-2013 |
| HMMR | CCNB1 | 0.008199 | Co-expression | Mallon-McKay-2013 |
| HMMR | KIF23 | 0.007226 | Co-expression | Mallon-McKay-2013 |
| HMMR | NDC80 | 0.01156 | Co-expression | Mallon-McKay-2013 |
| KIF11 | AURKA | 0.024796 | Co-expression | Mallon-McKay-2013 |
| KIF11 | KIF23 | 0.013992 | Co-expression | Mallon-McKay-2013 |
| KIF23 | BUB1B | 0.005602 | Co-expression | Mallon-McKay-2013 |
| KIF23 | CCNB1 | 0.004137 | Co-expression | Mallon-McKay-2013 |
| KIF23 | KIF20A | 0.005778 | Co-expression | Mallon-McKay-2013 |
| KIF4A | CENPE | 0.013065 | Co-expression | Mallon-McKay-2013 |
| KIF4A | CENPF | 0.014907 | Co-expression | Mallon-McKay-2013 |
| KIF4A | KIF20A | 0.0102 | Co-expression | Mallon-McKay-2013 |
| KIF4A | KIF23 | 0.005906 | Co-expression | Mallon-McKay-2013 |
| KIF4A | NUSAP1 | 0.010552 | Co-expression | Mallon-McKay-2013 |
| KIF4A | TOP2A | 0.009332 | Co-expression | Mallon-McKay-2013 |
| NDC80 | CCNB1 | 0.006583 | Co-expression | Mallon-McKay-2013 |
| NDC80 | KIF23 | 0.005811 | Co-expression | Mallon-McKay-2013 |
| NUSAP1 | KIF20A | 0.010304 | Co-expression | Mallon-McKay-2013 |
| SMC4 | CDK1 | 0.014572 | Co-expression | Mallon-McKay-2013 |
| SMC4 | DEPDC1 | 0.021116 | Co-expression | Mallon-McKay-2013 |
| SMC4 | KIF4A | 0.009535 | Co-expression | Mallon-McKay-2013 |
| SMC4 | MKI67 | 0.014902 | Co-expression | Mallon-McKay-2013 |
| SMC4 | NUSAP1 | 0.009987 | Co-expression | Mallon-McKay-2013 |
| SMC4 | ZWINT | 0.011639 | Co-expression | Mallon-McKay-2013 |
| TOP2A | CENPF | 0.012481 | Co-expression | Mallon-McKay-2013 |
| TOP2A | KIF20A | 0.009164 | Co-expression | Mallon-McKay-2013 |
| TOP2A | NUSAP1 | 0.009935 | Co-expression | Mallon-McKay-2013 |
| ZWINT | CDK1 | 0.018805 | Co-expression | Mallon-McKay-2013 |
| ZWINT | KIF23 | 0.007343 | Co-expression | Mallon-McKay-2013 |
| ZWINT | NDC80 | 0.01169 | Co-expression | Mallon-McKay-2013 |
| ASPM | CENPE | 0.077477 | Co-expression | Noble-Diehl-2008 |
| ASPM | CENPF | 0.09853 | Co-expression | Noble-Diehl-2008 |
| ASPM | PRC1 | 0.081942 | Co-expression | Noble-Diehl-2008 |
| AURKA | CCNB1 | 0.070389 | Co-expression | Noble-Diehl-2008 |
| AURKA | CENPE | 0.073189 | Co-expression | Noble-Diehl-2008 |
| AURKA | KIF20A | 0.048214 | Co-expression | Noble-Diehl-2008 |
| AURKB | CCNA2 | 0.039937 | Co-expression | Noble-Diehl-2008 |
| AURKB | GINS1 | 0.056042 | Co-expression | Noble-Diehl-2008 |
| AURKB | KIF20A | 0.047369 | Co-expression | Noble-Diehl-2008 |
| AURKB | KIF23 | 0.052582 | Co-expression | Noble-Diehl-2008 |
| AURKB | MKI67 | 0.044805 | Co-expression | Noble-Diehl-2008 |
| AURKB | NEK2 | 0.095179 | Co-expression | Noble-Diehl-2008 |
| AURKB | TOP2A | 0.062368 | Co-expression | Noble-Diehl-2008 |
| CCNA2 | CCNB1 | 0.025689 | Co-expression | Noble-Diehl-2008 |
| CCNA2 | KIF20A | 0.023545 | Co-expression | Noble-Diehl-2008 |
| CCNA2 | KIF23 | 0.023542 | Co-expression | Noble-Diehl-2008 |
| CCNA2 | NEK2 | 0.042778 | Co-expression | Noble-Diehl-2008 |
| CCNA2 | NUSAP1 | 0.024589 | Co-expression | Noble-Diehl-2008 |
| CCNB1 | KIF20A | 0.038394 | Co-expression | Noble-Diehl-2008 |
| CCNB1 | PRC1 | 0.040862 | Co-expression | Noble-Diehl-2008 |
| CDC25C | BUB1B | 0.026578 | Co-expression | Noble-Diehl-2008 |
| CDK1 | CCNA2 | 0.040252 | Co-expression | Noble-Diehl-2008 |
| CDK1 | CCNB1 | 0.064029 | Co-expression | Noble-Diehl-2008 |
| CENPE | CCNA2 | 0.032913 | Co-expression | Noble-Diehl-2008 |
| CENPE | CCNB1 | 0.051004 | Co-expression | Noble-Diehl-2008 |
| CENPE | KIF20A | 0.039403 | Co-expression | Noble-Diehl-2008 |
| CENPE | NUSAP1 | 0.045644 | Co-expression | Noble-Diehl-2008 |
| CENPE | PRC1 | 0.064173 | Co-expression | Noble-Diehl-2008 |
| CENPF | CCNB1 | 0.053139 | Co-expression | Noble-Diehl-2008 |
| CENPF | KIF20A | 0.045494 | Co-expression | Noble-Diehl-2008 |
| CENPF | NEK2 | 0.077341 | Co-expression | Noble-Diehl-2008 |
| DEPDC1 | CCNA2 | 0.048538 | Co-expression | Noble-Diehl-2008 |
| DEPDC1 | CENPF | 0.090547 | Co-expression | Noble-Diehl-2008 |
| DEPDC1 | HMMR | 0.066955 | Co-expression | Noble-Diehl-2008 |
| DEPDC1 | KIF11 | 0.108685 | Co-expression | Noble-Diehl-2008 |
| DEPDC1 | KIF20A | 0.057305 | Co-expression | Noble-Diehl-2008 |
| DEPDC1 | KIF23 | 0.065932 | Co-expression | Noble-Diehl-2008 |
| DEPDC1 | NEK2 | 0.122688 | Co-expression | Noble-Diehl-2008 |
| DEPDC1 | NUSAP1 | 0.074019 | Co-expression | Noble-Diehl-2008 |
| DEPDC1 | TOP2A | 0.070596 | Co-expression | Noble-Diehl-2008 |
| HMMR | AURKB | 0.045456 | Co-expression | Noble-Diehl-2008 |
| HMMR | CCNA2 | 0.02521 | Co-expression | Noble-Diehl-2008 |
| HMMR | CCNB1 | 0.034726 | Co-expression | Noble-Diehl-2008 |
| HMMR | CENPE | 0.048337 | Co-expression | Noble-Diehl-2008 |
| HMMR | CENPF | 0.048774 | Co-expression | Noble-Diehl-2008 |
| HMMR | KIF11 | 0.059829 | Co-expression | Noble-Diehl-2008 |
| HMMR | KIF20A | 0.030351 | Co-expression | Noble-Diehl-2008 |
| HMMR | KIF23 | 0.03053 | Co-expression | Noble-Diehl-2008 |
| HMMR | NEK2 | 0.054383 | Co-expression | Noble-Diehl-2008 |
| HMMR | NUSAP1 | 0.035303 | Co-expression | Noble-Diehl-2008 |
| KIF11 | AURKB | 0.091494 | Co-expression | Noble-Diehl-2008 |
| KIF11 | CCNA2 | 0.04429 | Co-expression | Noble-Diehl-2008 |
| KIF11 | CCNB1 | 0.062131 | Co-expression | Noble-Diehl-2008 |
| KIF11 | CENPE | 0.087566 | Co-expression | Noble-Diehl-2008 |
| KIF11 | KIF20A | 0.053432 | Co-expression | Noble-Diehl-2008 |
| KIF11 | KIF23 | 0.058318 | Co-expression | Noble-Diehl-2008 |
| KIF11 | NEK2 | 0.095456 | Co-expression | Noble-Diehl-2008 |
| KIF11 | NUSAP1 | 0.060575 | Co-expression | Noble-Diehl-2008 |
| KIF20A | NEK2 | 0.051963 | Co-expression | Noble-Diehl-2008 |
| KIF23 | BUB1B | 0.029683 | Co-expression | Noble-Diehl-2008 |
| KIF23 | NEK2 | 0.055617 | Co-expression | Noble-Diehl-2008 |
| KIF23 | NUSAP1 | 0.041773 | Co-expression | Noble-Diehl-2008 |
| KIF23 | PRC1 | 0.041198 | Co-expression | Noble-Diehl-2008 |
| KIF4A | AURKB | 0.070058 | Co-expression | Noble-Diehl-2008 |
| KIF4A | CCNA2 | 0.026882 | Co-expression | Noble-Diehl-2008 |
| KIF4A | CENPE | 0.057419 | Co-expression | Noble-Diehl-2008 |
| KIF4A | GINS1 | 0.053792 | Co-expression | Noble-Diehl-2008 |
| KIF4A | KIF11 | 0.07731 | Co-expression | Noble-Diehl-2008 |
| KIF4A | KIF20A | 0.036001 | Co-expression | Noble-Diehl-2008 |
| KIF4A | KIF23 | 0.045201 | Co-expression | Noble-Diehl-2008 |
| KIF4A | NUSAP1 | 0.07013 | Co-expression | Noble-Diehl-2008 |
| MKI67 | CENPF | 0.04654 | Co-expression | Noble-Diehl-2008 |
| MKI67 | GINS1 | 0.037363 | Co-expression | Noble-Diehl-2008 |
| MKI67 | KIF20A | 0.029781 | Co-expression | Noble-Diehl-2008 |
| MKI67 | NEK2 | 0.046579 | Co-expression | Noble-Diehl-2008 |
| NDC80 | CCNB1 | 0.072943 | Co-expression | Noble-Diehl-2008 |
| NDC80 | KIF20A | 0.057479 | Co-expression | Noble-Diehl-2008 |
| NUSAP1 | NEK2 | 0.060261 | Co-expression | Noble-Diehl-2008 |
| SMC4 | CCNA2 | 0.014089 | Co-expression | Noble-Diehl-2008 |
| SMC4 | CCNB1 | 0.024678 | Co-expression | Noble-Diehl-2008 |
| SMC4 | CENPF | 0.030632 | Co-expression | Noble-Diehl-2008 |
| SMC4 | DEPDC1 | 0.035557 | Co-expression | Noble-Diehl-2008 |
| SMC4 | HMMR | 0.022094 | Co-expression | Noble-Diehl-2008 |
| SMC4 | KIF11 | 0.033878 | Co-expression | Noble-Diehl-2008 |
| SMC4 | KIF20A | 0.022229 | Co-expression | Noble-Diehl-2008 |
| SMC4 | MKI67 | 0.02119 | Co-expression | Noble-Diehl-2008 |
| SMC4 | NDC80 | 0.038982 | Co-expression | Noble-Diehl-2008 |
| SMC4 | NEK2 | 0.032159 | Co-expression | Noble-Diehl-2008 |
| SMC4 | TOP2A | 0.031307 | Co-expression | Noble-Diehl-2008 |
| TOP2A | CCNB1 | 0.048389 | Co-expression | Noble-Diehl-2008 |
| TOP2A | CENPF | 0.062282 | Co-expression | Noble-Diehl-2008 |
| TOP2A | KIF20A | 0.043999 | Co-expression | Noble-Diehl-2008 |
| TOP2A | MKI67 | 0.041995 | Co-expression | Noble-Diehl-2008 |
| TOP2A | NDC80 | 0.080144 | Co-expression | Noble-Diehl-2008 |
| TOP2A | NEK2 | 0.064625 | Co-expression | Noble-Diehl-2008 |
| AURKB | PRC1 | 0.105967 | Co-expression | Ramaswamy-Golub-2001 |
| BUB1B | NUSAP1 | 0.091376 | Co-expression | Ramaswamy-Golub-2001 |
| CCNA2 | BUB1B | 0.14404 | Co-expression | Ramaswamy-Golub-2001 |
| CCNA2 | CCNB1 | 0.102876 | Co-expression | Ramaswamy-Golub-2001 |
| CCNA2 | NUSAP1 | 0.090736 | Co-expression | Ramaswamy-Golub-2001 |
| CDC25C | CCNB1 | 0.098987 | Co-expression | Ramaswamy-Golub-2001 |
| CDCA3 | AURKA | 0.111258 | Co-expression | Ramaswamy-Golub-2001 |
| CDK1 | BUB1B | 0.142609 | Co-expression | Ramaswamy-Golub-2001 |
| CDK1 | CCNA2 | 0.142361 | Co-expression | Ramaswamy-Golub-2001 |
| CDK1 | NUSAP1 | 0.080096 | Co-expression | Ramaswamy-Golub-2001 |
| CENPE | GINS1 | 0.088293 | Co-expression | Ramaswamy-Golub-2001 |
| CENPF | GINS1 | 0.092613 | Co-expression | Ramaswamy-Golub-2001 |
| KIF11 | BUB1B | 0.097642 | Co-expression | Ramaswamy-Golub-2001 |
| KIF11 | GINS1 | 0.086727 | Co-expression | Ramaswamy-Golub-2001 |
| KIF11 | MKI67 | 0.103188 | Co-expression | Ramaswamy-Golub-2001 |
| KIF11 | TOP2A | 0.085798 | Co-expression | Ramaswamy-Golub-2001 |
| KIF23 | GINS1 | 0.069227 | Co-expression | Ramaswamy-Golub-2001 |
| KIF4A | AURKB | 0.104381 | Co-expression | Ramaswamy-Golub-2001 |
| KIF4A | BUB1B | 0.108271 | Co-expression | Ramaswamy-Golub-2001 |
| KIF4A | CCNA2 | 0.112467 | Co-expression | Ramaswamy-Golub-2001 |
| KIF4A | CDK1 | 0.110516 | Co-expression | Ramaswamy-Golub-2001 |
| MKI67 | GINS1 | 0.076312 | Co-expression | Ramaswamy-Golub-2001 |
| PRC1 | NUSAP1 | 0.097343 | Co-expression | Ramaswamy-Golub-2001 |
| SMC4 | NUSAP1 | 0.082892 | Co-expression | Ramaswamy-Golub-2001 |
| TOP2A | BUB1B | 0.084124 | Co-expression | Ramaswamy-Golub-2001 |
| TOP2A | CCNA2 | 0.083316 | Co-expression | Ramaswamy-Golub-2001 |
| TOP2A | CCNB1 | 0.083096 | Co-expression | Ramaswamy-Golub-2001 |
| TOP2A | CENPF | 0.079485 | Co-expression | Ramaswamy-Golub-2001 |
| TOP2A | GINS1 | 0.074452 | Co-expression | Ramaswamy-Golub-2001 |
| TOP2A | MKI67 | 0.082541 | Co-expression | Ramaswamy-Golub-2001 |
| AURKA | BUB1B | 0.017602 | Co-expression | Rieger-Chu-2004 |
| AURKA | CCNA2 | 0.02244 | Co-expression | Rieger-Chu-2004 |
| AURKA | CCNB1 | 0.02212 | Co-expression | Rieger-Chu-2004 |
| AURKA | CCNF | 0.034712 | Co-expression | Rieger-Chu-2004 |
| AURKA | CENPE | 0.022427 | Co-expression | Rieger-Chu-2004 |
| AURKA | CENPF | 0.017939 | Co-expression | Rieger-Chu-2004 |
| AURKA | KIF23 | 0.021561 | Co-expression | Rieger-Chu-2004 |
| AURKA | MKI67 | 0.029102 | Co-expression | Rieger-Chu-2004 |
| AURKA | NDC80 | 0.03037 | Co-expression | Rieger-Chu-2004 |
| AURKA | NEK2 | 0.025687 | Co-expression | Rieger-Chu-2004 |
| AURKB | AURKA | 0.020709 | Co-expression | Rieger-Chu-2004 |
| AURKB | BUB1B | 0.012284 | Co-expression | Rieger-Chu-2004 |
| AURKB | CCNA2 | 0.023333 | Co-expression | Rieger-Chu-2004 |
| AURKB | CCNB1 | 0.017587 | Co-expression | Rieger-Chu-2004 |
| AURKB | CCNF | 0.034002 | Co-expression | Rieger-Chu-2004 |
| AURKB | CENPE | 0.017299 | Co-expression | Rieger-Chu-2004 |
| AURKB | MKI67 | 0.030485 | Co-expression | Rieger-Chu-2004 |
| AURKB | NDC80 | 0.022259 | Co-expression | Rieger-Chu-2004 |
| AURKB | NEK2 | 0.018791 | Co-expression | Rieger-Chu-2004 |
| AURKB | TOP2A | 0.019576 | Co-expression | Rieger-Chu-2004 |
| BUB1B | NEK2 | 0.018418 | Co-expression | Rieger-Chu-2004 |
| CCNA2 | BUB1B | 0.017494 | Co-expression | Rieger-Chu-2004 |
| CCNA2 | CCNB1 | 0.021301 | Co-expression | Rieger-Chu-2004 |
| CCNA2 | CCNF | 0.036522 | Co-expression | Rieger-Chu-2004 |
| CCNA2 | CENPF | 0.018029 | Co-expression | Rieger-Chu-2004 |
| CCNA2 | KIF23 | 0.021885 | Co-expression | Rieger-Chu-2004 |
| CCNA2 | NEK2 | 0.02281 | Co-expression | Rieger-Chu-2004 |
| CCNB1 | BUB1B | 0.016533 | Co-expression | Rieger-Chu-2004 |
| CCNB1 | NEK2 | 0.023815 | Co-expression | Rieger-Chu-2004 |
| CCNF | BUB1B | 0.027024 | Co-expression | Rieger-Chu-2004 |
| CCNF | CCNB1 | 0.031389 | Co-expression | Rieger-Chu-2004 |
| CCNF | CENPF | 0.027881 | Co-expression | Rieger-Chu-2004 |
| CCNF | KIF23 | 0.034911 | Co-expression | Rieger-Chu-2004 |
| CCNF | NEK2 | 0.032642 | Co-expression | Rieger-Chu-2004 |
| CDC25C | AURKA | 0.019082 | Co-expression | Rieger-Chu-2004 |
| CDC25C | AURKB | 0.027853 | Co-expression | Rieger-Chu-2004 |
| CDC25C | CCNA2 | 0.01887 | Co-expression | Rieger-Chu-2004 |
| CDC25C | CCNB1 | 0.014473 | Co-expression | Rieger-Chu-2004 |
| CDC25C | CENPE | 0.015249 | Co-expression | Rieger-Chu-2004 |
| CDC25C | CENPF | 0.019964 | Co-expression | Rieger-Chu-2004 |
| CDC25C | HMMR | 0.013364 | Co-expression | Rieger-Chu-2004 |
| CDC25C | NDC80 | 0.021999 | Co-expression | Rieger-Chu-2004 |
| CDC25C | NEK2 | 0.021827 | Co-expression | Rieger-Chu-2004 |
| CDK1 | AURKA | 0.033564 | Co-expression | Rieger-Chu-2004 |
| CDK1 | AURKB | 0.034182 | Co-expression | Rieger-Chu-2004 |
| CDK1 | BUB1B | 0.026197 | Co-expression | Rieger-Chu-2004 |
| CDK1 | CCNA2 | 0.038093 | Co-expression | Rieger-Chu-2004 |
| CDK1 | CCNB1 | 0.031252 | Co-expression | Rieger-Chu-2004 |
| CDK1 | CENPE | 0.028156 | Co-expression | Rieger-Chu-2004 |
| CDK1 | CENPF | 0.026285 | Co-expression | Rieger-Chu-2004 |
| CDK1 | KIF11 | 0.027552 | Co-expression | Rieger-Chu-2004 |
| CDK1 | KIF23 | 0.0325 | Co-expression | Rieger-Chu-2004 |
| CDK1 | MKI67 | 0.047378 | Co-expression | Rieger-Chu-2004 |
| CDK1 | NDC80 | 0.047559 | Co-expression | Rieger-Chu-2004 |
| CDK1 | NEK2 | 0.037706 | Co-expression | Rieger-Chu-2004 |
| CDK1 | TOP2A | 0.039359 | Co-expression | Rieger-Chu-2004 |
| CENPE | BUB1B | 0.017171 | Co-expression | Rieger-Chu-2004 |
| CENPE | CCNA2 | 0.021091 | Co-expression | Rieger-Chu-2004 |
| CENPE | CCNB1 | 0.020233 | Co-expression | Rieger-Chu-2004 |
| CENPE | CCNF | 0.033253 | Co-expression | Rieger-Chu-2004 |
| CENPE | CENPF | 0.017883 | Co-expression | Rieger-Chu-2004 |
| CENPE | KIF23 | 0.02025 | Co-expression | Rieger-Chu-2004 |
| CENPE | NEK2 | 0.023977 | Co-expression | Rieger-Chu-2004 |
| CENPF | BUB1B | 0.012894 | Co-expression | Rieger-Chu-2004 |
| CENPF | CCNB1 | 0.016602 | Co-expression | Rieger-Chu-2004 |
| CENPF | NEK2 | 0.021558 | Co-expression | Rieger-Chu-2004 |
| HMMR | AURKA | 0.021203 | Co-expression | Rieger-Chu-2004 |
| HMMR | AURKB | 0.015061 | Co-expression | Rieger-Chu-2004 |
| HMMR | BUB1B | 0.016764 | Co-expression | Rieger-Chu-2004 |
| HMMR | CCNA2 | 0.020037 | Co-expression | Rieger-Chu-2004 |
| HMMR | CCNB1 | 0.019824 | Co-expression | Rieger-Chu-2004 |
| HMMR | CCNF | 0.03074 | Co-expression | Rieger-Chu-2004 |
| HMMR | CDK1 | 0.029829 | Co-expression | Rieger-Chu-2004 |
| HMMR | CENPE | 0.020295 | Co-expression | Rieger-Chu-2004 |
| HMMR | CENPF | 0.017518 | Co-expression | Rieger-Chu-2004 |
| HMMR | KIF11 | 0.016799 | Co-expression | Rieger-Chu-2004 |
| HMMR | KIF23 | 0.021133 | Co-expression | Rieger-Chu-2004 |
| HMMR | MKI67 | 0.027645 | Co-expression | Rieger-Chu-2004 |
| HMMR | NDC80 | 0.025883 | Co-expression | Rieger-Chu-2004 |
| HMMR | NEK2 | 0.023069 | Co-expression | Rieger-Chu-2004 |
| HMMR | TOP2A | 0.022434 | Co-expression | Rieger-Chu-2004 |
| KIF11 | AURKA | 0.017454 | Co-expression | Rieger-Chu-2004 |
| KIF11 | AURKB | 0.013786 | Co-expression | Rieger-Chu-2004 |
| KIF11 | BUB1B | 0.014568 | Co-expression | Rieger-Chu-2004 |
| KIF11 | CCNA2 | 0.018224 | Co-expression | Rieger-Chu-2004 |
| KIF11 | CCNB1 | 0.015772 | Co-expression | Rieger-Chu-2004 |
| KIF11 | CCNF | 0.025373 | Co-expression | Rieger-Chu-2004 |
| KIF11 | CENPE | 0.01655 | Co-expression | Rieger-Chu-2004 |
| KIF11 | CENPF | 0.014268 | Co-expression | Rieger-Chu-2004 |
| KIF11 | KIF23 | 0.017863 | Co-expression | Rieger-Chu-2004 |
| KIF11 | MKI67 | 0.025847 | Co-expression | Rieger-Chu-2004 |
| KIF11 | NDC80 | 0.020439 | Co-expression | Rieger-Chu-2004 |
| KIF11 | NEK2 | 0.017275 | Co-expression | Rieger-Chu-2004 |
| KIF11 | TOP2A | 0.01885 | Co-expression | Rieger-Chu-2004 |
| KIF23 | BUB1B | 0.018827 | Co-expression | Rieger-Chu-2004 |
| KIF23 | CCNB1 | 0.021855 | Co-expression | Rieger-Chu-2004 |
| KIF23 | CENPF | 0.01684 | Co-expression | Rieger-Chu-2004 |
| KIF23 | NEK2 | 0.022267 | Co-expression | Rieger-Chu-2004 |
| MKI67 | BUB1B | 0.024936 | Co-expression | Rieger-Chu-2004 |
| MKI67 | CCNA2 | 0.036335 | Co-expression | Rieger-Chu-2004 |
| MKI67 | CCNB1 | 0.027436 | Co-expression | Rieger-Chu-2004 |
| MKI67 | CCNF | 0.054233 | Co-expression | Rieger-Chu-2004 |
| MKI67 | CENPE | 0.029839 | Co-expression | Rieger-Chu-2004 |
| MKI67 | CENPF | 0.027085 | Co-expression | Rieger-Chu-2004 |
| MKI67 | KIF23 | 0.03005 | Co-expression | Rieger-Chu-2004 |
| MKI67 | NEK2 | 0.028546 | Co-expression | Rieger-Chu-2004 |
| NDC80 | BUB1B | 0.020608 | Co-expression | Rieger-Chu-2004 |
| NDC80 | CCNA2 | 0.026557 | Co-expression | Rieger-Chu-2004 |
| NDC80 | CCNB1 | 0.025828 | Co-expression | Rieger-Chu-2004 |
| NDC80 | CENPE | 0.026253 | Co-expression | Rieger-Chu-2004 |
| NDC80 | CENPF | 0.021192 | Co-expression | Rieger-Chu-2004 |
| NDC80 | KIF23 | 0.026581 | Co-expression | Rieger-Chu-2004 |
| NDC80 | NEK2 | 0.033924 | Co-expression | Rieger-Chu-2004 |
| SMC4 | BUB1B | 0.013528 | Co-expression | Rieger-Chu-2004 |
| SMC4 | HMMR | 0.013981 | Co-expression | Rieger-Chu-2004 |
| SMC4 | KIF11 | 0.015513 | Co-expression | Rieger-Chu-2004 |
| SMC4 | TOP2A | 0.019187 | Co-expression | Rieger-Chu-2004 |
| TOP2A | AURKA | 0.023518 | Co-expression | Rieger-Chu-2004 |
| TOP2A | BUB1B | 0.018975 | Co-expression | Rieger-Chu-2004 |
| TOP2A | CCNA2 | 0.024655 | Co-expression | Rieger-Chu-2004 |
| TOP2A | CCNB1 | 0.020085 | Co-expression | Rieger-Chu-2004 |
| TOP2A | CCNF | 0.03467 | Co-expression | Rieger-Chu-2004 |
| TOP2A | CENPE | 0.02306 | Co-expression | Rieger-Chu-2004 |
| TOP2A | CENPF | 0.020417 | Co-expression | Rieger-Chu-2004 |
| TOP2A | KIF23 | 0.018866 | Co-expression | Rieger-Chu-2004 |
| TOP2A | MKI67 | 0.031628 | Co-expression | Rieger-Chu-2004 |
| TOP2A | NDC80 | 0.029846 | Co-expression | Rieger-Chu-2004 |
| TOP2A | NEK2 | 0.026191 | Co-expression | Rieger-Chu-2004 |
| ZWINT | GINS1 | 0.019953 | Co-expression | Rieger-Chu-2004 |
| AURKB | CCNA2 | 6.44E-05 | Co-expression | Rosenwald-Staudt-2001 |
| AURKB | CCNB1 | 5.17E-05 | Co-expression | Rosenwald-Staudt-2001 |
| AURKB | CCNF | 9.23E-05 | Co-expression | Rosenwald-Staudt-2001 |
| AURKB | CENPE | 6.82E-05 | Co-expression | Rosenwald-Staudt-2001 |
| AURKB | CENPF | 8.40E-05 | Co-expression | Rosenwald-Staudt-2001 |
| AURKB | KIF23 | 5.23E-05 | Co-expression | Rosenwald-Staudt-2001 |
| AURKB | MKI67 | 4.44E-05 | Co-expression | Rosenwald-Staudt-2001 |
| AURKB | NEK2 | 4.96E-05 | Co-expression | Rosenwald-Staudt-2001 |
| AURKB | TOP2A | 9.79E-05 | Co-expression | Rosenwald-Staudt-2001 |
| CCNA2 | CCNB1 | 5.27E-05 | Co-expression | Rosenwald-Staudt-2001 |
| CCNA2 | CCNF | 9.56E-05 | Co-expression | Rosenwald-Staudt-2001 |
| CCNA2 | CENPF | 8.87E-05 | Co-expression | Rosenwald-Staudt-2001 |
| CCNA2 | KIF23 | 5.59E-05 | Co-expression | Rosenwald-Staudt-2001 |
| CCNA2 | NEK2 | 5.41E-05 | Co-expression | Rosenwald-Staudt-2001 |
| CCNB1 | NEK2 | 4.22E-05 | Co-expression | Rosenwald-Staudt-2001 |
| CCNF | CCNB1 | 7.75E-05 | Co-expression | Rosenwald-Staudt-2001 |
| CCNF | KIF23 | 8.23E-05 | Co-expression | Rosenwald-Staudt-2001 |
| CCNF | NEK2 | 8.25E-05 | Co-expression | Rosenwald-Staudt-2001 |
| CDC25C | AURKB | 7.06E-05 | Co-expression | Rosenwald-Staudt-2001 |
| CDC25C | CCNA2 | 7.31E-05 | Co-expression | Rosenwald-Staudt-2001 |
| CDC25C | CCNB1 | 6.40E-05 | Co-expression | Rosenwald-Staudt-2001 |
| CDC25C | CCNF | 1.11E-04 | Co-expression | Rosenwald-Staudt-2001 |
| CDC25C | CDK1 | 8.17E-05 | Co-expression | Rosenwald-Staudt-2001 |
| CDC25C | CENPE | 8.14E-05 | Co-expression | Rosenwald-Staudt-2001 |
| CDC25C | CENPF | 9.96E-05 | Co-expression | Rosenwald-Staudt-2001 |
| CDC25C | DEPDC1 | 5.81E-05 | Co-expression | Rosenwald-Staudt-2001 |
| CDC25C | HMMR | 8.28E-05 | Co-expression | Rosenwald-Staudt-2001 |
| CDC25C | KIF23 | 6.18E-05 | Co-expression | Rosenwald-Staudt-2001 |
| CDC25C | MKI67 | 5.39E-05 | Co-expression | Rosenwald-Staudt-2001 |
| CDC25C | NEK2 | 6.13E-05 | Co-expression | Rosenwald-Staudt-2001 |
| CDC25C | TOP2A | 1.15E-04 | Co-expression | Rosenwald-Staudt-2001 |
| CDK1 | AURKB | 6.32E-05 | Co-expression | Rosenwald-Staudt-2001 |
| CDK1 | CCNA2 | 6.85E-05 | Co-expression | Rosenwald-Staudt-2001 |
| CDK1 | CCNB1 | 5.56E-05 | Co-expression | Rosenwald-Staudt-2001 |
| CDK1 | CCNF | 9.88E-05 | Co-expression | Rosenwald-Staudt-2001 |
| CDK1 | CENPE | 7.94E-05 | Co-expression | Rosenwald-Staudt-2001 |
| CDK1 | CENPF | 9.71E-05 | Co-expression | Rosenwald-Staudt-2001 |
| CDK1 | KIF23 | 6.18E-05 | Co-expression | Rosenwald-Staudt-2001 |
| CDK1 | MKI67 | 5.29E-05 | Co-expression | Rosenwald-Staudt-2001 |
| CDK1 | NEK2 | 5.79E-05 | Co-expression | Rosenwald-Staudt-2001 |
| CDK1 | TOP2A | 1.17E-04 | Co-expression | Rosenwald-Staudt-2001 |
| CENPE | CCNA2 | 7.51E-05 | Co-expression | Rosenwald-Staudt-2001 |
| CENPE | CCNB1 | 5.70E-05 | Co-expression | Rosenwald-Staudt-2001 |
| CENPE | CENPF | 1.02E-04 | Co-expression | Rosenwald-Staudt-2001 |
| CENPE | KIF23 | 6.18E-05 | Co-expression | Rosenwald-Staudt-2001 |
| CENPE | NEK2 | 5.95E-05 | Co-expression | Rosenwald-Staudt-2001 |
| CENPF | CCNB1 | 7.23E-05 | Co-expression | Rosenwald-Staudt-2001 |
| CENPF | NEK2 | 7.41E-05 | Co-expression | Rosenwald-Staudt-2001 |
| DEPDC1 | AURKB | 4.71E-05 | Co-expression | Rosenwald-Staudt-2001 |
| DEPDC1 | CCNA2 | 5.01E-05 | Co-expression | Rosenwald-Staudt-2001 |
| DEPDC1 | CCNB1 | 3.91E-05 | Co-expression | Rosenwald-Staudt-2001 |
| DEPDC1 | CCNF | 7.82E-05 | Co-expression | Rosenwald-Staudt-2001 |
| DEPDC1 | CDK1 | 5.62E-05 | Co-expression | Rosenwald-Staudt-2001 |
| DEPDC1 | CENPE | 6.12E-05 | Co-expression | Rosenwald-Staudt-2001 |
| DEPDC1 | CENPF | 6.80E-05 | Co-expression | Rosenwald-Staudt-2001 |
| DEPDC1 | HMMR | 5.37E-05 | Co-expression | Rosenwald-Staudt-2001 |
| DEPDC1 | KIF23 | 4.36E-05 | Co-expression | Rosenwald-Staudt-2001 |
| DEPDC1 | MKI67 | 3.76E-05 | Co-expression | Rosenwald-Staudt-2001 |
| DEPDC1 | NEK2 | 4.17E-05 | Co-expression | Rosenwald-Staudt-2001 |
| DEPDC1 | TOP2A | 8.28E-05 | Co-expression | Rosenwald-Staudt-2001 |
| HMMR | CCNA2 | 6.84E-05 | Co-expression | Rosenwald-Staudt-2001 |
| HMMR | CCNB1 | 5.69E-05 | Co-expression | Rosenwald-Staudt-2001 |
| HMMR | CDK1 | 7.42E-05 | Co-expression | Rosenwald-Staudt-2001 |
| HMMR | CENPF | 9.93E-05 | Co-expression | Rosenwald-Staudt-2001 |
| HMMR | KIF23 | 6.02E-05 | Co-expression | Rosenwald-Staudt-2001 |
| HMMR | MKI67 | 5.07E-05 | Co-expression | Rosenwald-Staudt-2001 |
| HMMR | NEK2 | 5.47E-05 | Co-expression | Rosenwald-Staudt-2001 |
| KIF23 | CCNB1 | 4.58E-05 | Co-expression | Rosenwald-Staudt-2001 |
| KIF23 | CENPF | 7.72E-05 | Co-expression | Rosenwald-Staudt-2001 |
| KIF23 | NEK2 | 4.58E-05 | Co-expression | Rosenwald-Staudt-2001 |
| MKI67 | CCNA2 | 4.59E-05 | Co-expression | Rosenwald-Staudt-2001 |
| MKI67 | CCNB1 | 3.81E-05 | Co-expression | Rosenwald-Staudt-2001 |
| MKI67 | CCNF | 6.87E-05 | Co-expression | Rosenwald-Staudt-2001 |
| MKI67 | CENPE | 5.60E-05 | Co-expression | Rosenwald-Staudt-2001 |
| MKI67 | CENPF | 6.74E-05 | Co-expression | Rosenwald-Staudt-2001 |
| MKI67 | KIF23 | 4.16E-05 | Co-expression | Rosenwald-Staudt-2001 |
| MKI67 | NEK2 | 3.78E-05 | Co-expression | Rosenwald-Staudt-2001 |
| TOP2A | CCNA2 | 1.06E-04 | Co-expression | Rosenwald-Staudt-2001 |
| TOP2A | CCNB1 | 8.18E-05 | Co-expression | Rosenwald-Staudt-2001 |
| TOP2A | KIF23 | 8.97E-05 | Co-expression | Rosenwald-Staudt-2001 |
| TOP2A | MKI67 | 7.31E-05 | Co-expression | Rosenwald-Staudt-2001 |
| TOP2A | NEK2 | 8.55E-05 | Co-expression | Rosenwald-Staudt-2001 |
| ASPM | AURKB | 0.323551 | Co-expression | Roth-Zlotnik-2006 |
| ASPM | BUB1B | 0.125454 | Co-expression | Roth-Zlotnik-2006 |
| ASPM | CCNA2 | 0.257104 | Co-expression | Roth-Zlotnik-2006 |
| ASPM | CCNB1 | 0.240406 | Co-expression | Roth-Zlotnik-2006 |
| ASPM | CDK1 | 0.288729 | Co-expression | Roth-Zlotnik-2006 |
| ASPM | CENPE | 0.185184 | Co-expression | Roth-Zlotnik-2006 |
| ASPM | CENPF | 0.33424 | Co-expression | Roth-Zlotnik-2006 |
| ASPM | HMMR | 0.14058 | Co-expression | Roth-Zlotnik-2006 |
| ASPM | KIF11 | 0.170882 | Co-expression | Roth-Zlotnik-2006 |
| ASPM | KIF20A | 0.170815 | Co-expression | Roth-Zlotnik-2006 |
| ASPM | KIF23 | 0.314832 | Co-expression | Roth-Zlotnik-2006 |
| ASPM | KIF4A | 0.209344 | Co-expression | Roth-Zlotnik-2006 |
| ASPM | MKI67 | 0.156152 | Co-expression | Roth-Zlotnik-2006 |
| ASPM | NUSAP1 | 0.198776 | Co-expression | Roth-Zlotnik-2006 |
| ASPM | PRC1 | 0.157561 | Co-expression | Roth-Zlotnik-2006 |
| ASPM | TOP2A | 0.224636 | Co-expression | Roth-Zlotnik-2006 |
| ASPM | ZWINT | 0.16626 | Co-expression | Roth-Zlotnik-2006 |
| AURKA | BUB1B | 0.183263 | Co-expression | Roth-Zlotnik-2006 |
| AURKA | CENPE | 0.239261 | Co-expression | Roth-Zlotnik-2006 |
| AURKA | GINS1 | 0.227072 | Co-expression | Roth-Zlotnik-2006 |
| AURKA | KIF20A | 0.213035 | Co-expression | Roth-Zlotnik-2006 |
| AURKA | NUSAP1 | 0.20919 | Co-expression | Roth-Zlotnik-2006 |
| AURKA | PRC1 | 0.206821 | Co-expression | Roth-Zlotnik-2006 |
| AURKB | MKI67 | 0.23507 | Co-expression | Roth-Zlotnik-2006 |
| AURKB | NUSAP1 | 0.230533 | Co-expression | Roth-Zlotnik-2006 |
| BUB1B | GINS1 | 0.113082 | Co-expression | Roth-Zlotnik-2006 |
| BUB1B | KIF20A | 0.101527 | Co-expression | Roth-Zlotnik-2006 |
| BUB1B | NUSAP1 | 0.092099 | Co-expression | Roth-Zlotnik-2006 |
| BUB1B | PRC1 | 0.097317 | Co-expression | Roth-Zlotnik-2006 |
| CCNA2 | BUB1B | 0.124242 | Co-expression | Roth-Zlotnik-2006 |
| CCNA2 | CCNB1 | 0.256801 | Co-expression | Roth-Zlotnik-2006 |
| CCNA2 | CENPF | 0.337238 | Co-expression | Roth-Zlotnik-2006 |
| CCNA2 | GINS1 | 0.171048 | Co-expression | Roth-Zlotnik-2006 |
| CCNA2 | KIF20A | 0.176208 | Co-expression | Roth-Zlotnik-2006 |
| CCNA2 | NUSAP1 | 0.196879 | Co-expression | Roth-Zlotnik-2006 |
| CCNA2 | PRC1 | 0.158862 | Co-expression | Roth-Zlotnik-2006 |
| CCNB1 | BUB1B | 0.124662 | Co-expression | Roth-Zlotnik-2006 |
| CCNB1 | GINS1 | 0.177003 | Co-expression | Roth-Zlotnik-2006 |
| CCNB1 | KIF20A | 0.181949 | Co-expression | Roth-Zlotnik-2006 |
| CCNB1 | NUSAP1 | 0.174763 | Co-expression | Roth-Zlotnik-2006 |
| CCNB1 | PRC1 | 0.154106 | Co-expression | Roth-Zlotnik-2006 |
| CDCA3 | AURKA | 0.262197 | Co-expression | Roth-Zlotnik-2006 |
| CDCA3 | BUB1B | 0.115349 | Co-expression | Roth-Zlotnik-2006 |
| CDCA3 | CCNB1 | 0.16175 | Co-expression | Roth-Zlotnik-2006 |
| CDCA3 | CDK1 | 0.211503 | Co-expression | Roth-Zlotnik-2006 |
| CDCA3 | CENPE | 0.128873 | Co-expression | Roth-Zlotnik-2006 |
| CDCA3 | GINS1 | 0.14378 | Co-expression | Roth-Zlotnik-2006 |
| CDCA3 | KIF20A | 0.134429 | Co-expression | Roth-Zlotnik-2006 |
| CDCA3 | KIF23 | 0.223713 | Co-expression | Roth-Zlotnik-2006 |
| CDCA3 | PRC1 | 0.118004 | Co-expression | Roth-Zlotnik-2006 |
| CDCA3 | TOP2A | 0.175312 | Co-expression | Roth-Zlotnik-2006 |
| CDK1 | BUB1B | 0.157366 | Co-expression | Roth-Zlotnik-2006 |
| CDK1 | CCNA2 | 0.299513 | Co-expression | Roth-Zlotnik-2006 |
| CDK1 | CCNB1 | 0.303382 | Co-expression | Roth-Zlotnik-2006 |
| CDK1 | CENPE | 0.22325 | Co-expression | Roth-Zlotnik-2006 |
| CDK1 | GINS1 | 0.208318 | Co-expression | Roth-Zlotnik-2006 |
| CDK1 | KIF11 | 0.184822 | Co-expression | Roth-Zlotnik-2006 |
| CDK1 | KIF20A | 0.231598 | Co-expression | Roth-Zlotnik-2006 |
| CDK1 | MKI67 | 0.193725 | Co-expression | Roth-Zlotnik-2006 |
| CDK1 | NUSAP1 | 0.203188 | Co-expression | Roth-Zlotnik-2006 |
| CDK1 | PRC1 | 0.179463 | Co-expression | Roth-Zlotnik-2006 |
| CDK1 | TOP2A | 0.27459 | Co-expression | Roth-Zlotnik-2006 |
| CENPE | BUB1B | 0.099173 | Co-expression | Roth-Zlotnik-2006 |
| CENPE | CCNA2 | 0.187858 | Co-expression | Roth-Zlotnik-2006 |
| CENPE | CCNB1 | 0.164609 | Co-expression | Roth-Zlotnik-2006 |
| CENPE | CENPF | 0.238056 | Co-expression | Roth-Zlotnik-2006 |
| CENPE | KIF20A | 0.125956 | Co-expression | Roth-Zlotnik-2006 |
| CENPE | KIF23 | 0.235316 | Co-expression | Roth-Zlotnik-2006 |
| CENPE | NUSAP1 | 0.137177 | Co-expression | Roth-Zlotnik-2006 |
| CENPE | PRC1 | 0.123921 | Co-expression | Roth-Zlotnik-2006 |
| CENPF | BUB1B | 0.161867 | Co-expression | Roth-Zlotnik-2006 |
| CENPF | CCNB1 | 0.311267 | Co-expression | Roth-Zlotnik-2006 |
| CENPF | KIF20A | 0.239211 | Co-expression | Roth-Zlotnik-2006 |
| CENPF | NUSAP1 | 0.237871 | Co-expression | Roth-Zlotnik-2006 |
| CENPF | PRC1 | 0.202068 | Co-expression | Roth-Zlotnik-2006 |
| DEPDC1 | BUB1B | 0.117203 | Co-expression | Roth-Zlotnik-2006 |
| DEPDC1 | HMMR | 0.129935 | Co-expression | Roth-Zlotnik-2006 |
| DEPDC1 | NEK2 | 0.349111 | Co-expression | Roth-Zlotnik-2006 |
| GINS1 | KIF20A | 0.133038 | Co-expression | Roth-Zlotnik-2006 |
| HMMR | AURKA | 0.218683 | Co-expression | Roth-Zlotnik-2006 |
| HMMR | BUB1B | 0.098062 | Co-expression | Roth-Zlotnik-2006 |
| HMMR | CCNA2 | 0.144002 | Co-expression | Roth-Zlotnik-2006 |
| HMMR | CCNB1 | 0.132937 | Co-expression | Roth-Zlotnik-2006 |
| HMMR | CDCA3 | 0.128493 | Co-expression | Roth-Zlotnik-2006 |
| HMMR | CDK1 | 0.183142 | Co-expression | Roth-Zlotnik-2006 |
| HMMR | CENPE | 0.119325 | Co-expression | Roth-Zlotnik-2006 |
| HMMR | CENPF | 0.189512 | Co-expression | Roth-Zlotnik-2006 |
| HMMR | GINS1 | 0.119477 | Co-expression | Roth-Zlotnik-2006 |
| HMMR | KIF11 | 0.089076 | Co-expression | Roth-Zlotnik-2006 |
| HMMR | KIF20A | 0.112194 | Co-expression | Roth-Zlotnik-2006 |
| HMMR | KIF23 | 0.199659 | Co-expression | Roth-Zlotnik-2006 |
| HMMR | NDC80 | 0.156345 | Co-expression | Roth-Zlotnik-2006 |
| HMMR | NUSAP1 | 0.105202 | Co-expression | Roth-Zlotnik-2006 |
| HMMR | PRC1 | 0.106783 | Co-expression | Roth-Zlotnik-2006 |
| HMMR | TOP2A | 0.147035 | Co-expression | Roth-Zlotnik-2006 |
| KIF11 | AURKA | 0.176432 | Co-expression | Roth-Zlotnik-2006 |
| KIF11 | AURKB | 0.202473 | Co-expression | Roth-Zlotnik-2006 |
| KIF11 | BUB1B | 0.077498 | Co-expression | Roth-Zlotnik-2006 |
| KIF11 | CCNA2 | 0.167467 | Co-expression | Roth-Zlotnik-2006 |
| KIF11 | CCNB1 | 0.157924 | Co-expression | Roth-Zlotnik-2006 |
| KIF11 | CENPE | 0.11939 | Co-expression | Roth-Zlotnik-2006 |
| KIF11 | CENPF | 0.207958 | Co-expression | Roth-Zlotnik-2006 |
| KIF11 | KIF20A | 0.106832 | Co-expression | Roth-Zlotnik-2006 |
| KIF11 | KIF23 | 0.202724 | Co-expression | Roth-Zlotnik-2006 |
| KIF11 | MKI67 | 0.10903 | Co-expression | Roth-Zlotnik-2006 |
| KIF11 | NUSAP1 | 0.124539 | Co-expression | Roth-Zlotnik-2006 |
| KIF11 | PRC1 | 0.102913 | Co-expression | Roth-Zlotnik-2006 |
| KIF11 | TOP2A | 0.135001 | Co-expression | Roth-Zlotnik-2006 |
| KIF23 | BUB1B | 0.170608 | Co-expression | Roth-Zlotnik-2006 |
| KIF23 | CCNB1 | 0.296598 | Co-expression | Roth-Zlotnik-2006 |
| KIF23 | GINS1 | 0.222971 | Co-expression | Roth-Zlotnik-2006 |
| KIF23 | KIF20A | 0.238736 | Co-expression | Roth-Zlotnik-2006 |
| KIF23 | NUSAP1 | 0.232732 | Co-expression | Roth-Zlotnik-2006 |
| KIF23 | PRC1 | 0.20891 | Co-expression | Roth-Zlotnik-2006 |
| KIF4A | AURKA | 0.230452 | Co-expression | Roth-Zlotnik-2006 |
| KIF4A | AURKB | 0.25791 | Co-expression | Roth-Zlotnik-2006 |
| KIF4A | CCNA2 | 0.209084 | Co-expression | Roth-Zlotnik-2006 |
| KIF4A | CCNB1 | 0.19248 | Co-expression | Roth-Zlotnik-2006 |
| KIF4A | CDK1 | 0.238132 | Co-expression | Roth-Zlotnik-2006 |
| KIF4A | CENPE | 0.157092 | Co-expression | Roth-Zlotnik-2006 |
| KIF4A | CENPF | 0.27217 | Co-expression | Roth-Zlotnik-2006 |
| KIF4A | KIF11 | 0.133953 | Co-expression | Roth-Zlotnik-2006 |
| KIF4A | KIF20A | 0.134917 | Co-expression | Roth-Zlotnik-2006 |
| KIF4A | KIF23 | 0.249774 | Co-expression | Roth-Zlotnik-2006 |
| KIF4A | MKI67 | 0.144051 | Co-expression | Roth-Zlotnik-2006 |
| KIF4A | NUSAP1 | 0.15125 | Co-expression | Roth-Zlotnik-2006 |
| KIF4A | PRC1 | 0.125749 | Co-expression | Roth-Zlotnik-2006 |
| KIF4A | TOP2A | 0.166121 | Co-expression | Roth-Zlotnik-2006 |
| KIF4A | ZWINT | 0.130893 | Co-expression | Roth-Zlotnik-2006 |
| MKI67 | CCNA2 | 0.164767 | Co-expression | Roth-Zlotnik-2006 |
| MKI67 | CCNF | 0.179608 | Co-expression | Roth-Zlotnik-2006 |
| MKI67 | CENPE | 0.121363 | Co-expression | Roth-Zlotnik-2006 |
| MKI67 | CENPF | 0.220103 | Co-expression | Roth-Zlotnik-2006 |
| MKI67 | KIF23 | 0.199664 | Co-expression | Roth-Zlotnik-2006 |
| MKI67 | NUSAP1 | 0.11644 | Co-expression | Roth-Zlotnik-2006 |
| NDC80 | BUB1B | 0.15312 | Co-expression | Roth-Zlotnik-2006 |
| NDC80 | GINS1 | 0.184025 | Co-expression | Roth-Zlotnik-2006 |
| NDC80 | KIF20A | 0.177185 | Co-expression | Roth-Zlotnik-2006 |
| NDC80 | PRC1 | 0.162562 | Co-expression | Roth-Zlotnik-2006 |
| NUSAP1 | KIF20A | 0.123925 | Co-expression | Roth-Zlotnik-2006 |
| PRC1 | GINS1 | 0.126059 | Co-expression | Roth-Zlotnik-2006 |
| PRC1 | KIF20A | 0.115553 | Co-expression | Roth-Zlotnik-2006 |
| PRC1 | NUSAP1 | 0.120333 | Co-expression | Roth-Zlotnik-2006 |
| SMC4 | BUB1B | 0.171636 | Co-expression | Roth-Zlotnik-2006 |
| SMC4 | CCNA2 | 0.343698 | Co-expression | Roth-Zlotnik-2006 |
| SMC4 | CCNB1 | 0.305318 | Co-expression | Roth-Zlotnik-2006 |
| SMC4 | CENPE | 0.218862 | Co-expression | Roth-Zlotnik-2006 |
| SMC4 | GINS1 | 0.234144 | Co-expression | Roth-Zlotnik-2006 |
| SMC4 | HMMR | 0.182477 | Co-expression | Roth-Zlotnik-2006 |
| SMC4 | KIF11 | 0.201576 | Co-expression | Roth-Zlotnik-2006 |
| SMC4 | KIF20A | 0.223863 | Co-expression | Roth-Zlotnik-2006 |
| SMC4 | NDC80 | 0.345548 | Co-expression | Roth-Zlotnik-2006 |
| SMC4 | NUSAP1 | 0.238785 | Co-expression | Roth-Zlotnik-2006 |
| SMC4 | PRC1 | 0.207769 | Co-expression | Roth-Zlotnik-2006 |
| SMC4 | TOP2A | 0.288454 | Co-expression | Roth-Zlotnik-2006 |
| SMC4 | ZWINT | 0.224598 | Co-expression | Roth-Zlotnik-2006 |
| TOP2A | AURKA | 0.295078 | Co-expression | Roth-Zlotnik-2006 |
| TOP2A | BUB1B | 0.134564 | Co-expression | Roth-Zlotnik-2006 |
| TOP2A | CCNA2 | 0.222684 | Co-expression | Roth-Zlotnik-2006 |
| TOP2A | CCNB1 | 0.219149 | Co-expression | Roth-Zlotnik-2006 |
| TOP2A | CENPE | 0.16328 | Co-expression | Roth-Zlotnik-2006 |
| TOP2A | CENPF | 0.294401 | Co-expression | Roth-Zlotnik-2006 |
| TOP2A | GINS1 | 0.172557 | Co-expression | Roth-Zlotnik-2006 |
| TOP2A | KIF20A | 0.17218 | Co-expression | Roth-Zlotnik-2006 |
| TOP2A | KIF23 | 0.286335 | Co-expression | Roth-Zlotnik-2006 |
| TOP2A | NUSAP1 | 0.161226 | Co-expression | Roth-Zlotnik-2006 |
| TOP2A | PRC1 | 0.150574 | Co-expression | Roth-Zlotnik-2006 |
| ZWINT | AURKA | 0.193724 | Co-expression | Roth-Zlotnik-2006 |
| ZWINT | BUB1B | 0.091443 | Co-expression | Roth-Zlotnik-2006 |
| ZWINT | CCNA2 | 0.174364 | Co-expression | Roth-Zlotnik-2006 |
| ZWINT | CCNB1 | 0.173031 | Co-expression | Roth-Zlotnik-2006 |
| ZWINT | CDCA3 | 0.115903 | Co-expression | Roth-Zlotnik-2006 |
| ZWINT | CDK1 | 0.19466 | Co-expression | Roth-Zlotnik-2006 |
| ZWINT | CENPE | 0.119927 | Co-expression | Roth-Zlotnik-2006 |
| ZWINT | CENPF | 0.211897 | Co-expression | Roth-Zlotnik-2006 |
| ZWINT | GINS1 | 0.125974 | Co-expression | Roth-Zlotnik-2006 |
| ZWINT | HMMR | 0.099013 | Co-expression | Roth-Zlotnik-2006 |
| ZWINT | KIF11 | 0.10737 | Co-expression | Roth-Zlotnik-2006 |
| ZWINT | KIF20A | 0.119257 | Co-expression | Roth-Zlotnik-2006 |
| ZWINT | KIF23 | 0.209859 | Co-expression | Roth-Zlotnik-2006 |
| ZWINT | NDC80 | 0.158011 | Co-expression | Roth-Zlotnik-2006 |
| ZWINT | NUSAP1 | 0.127468 | Co-expression | Roth-Zlotnik-2006 |
| ZWINT | PRC1 | 0.112876 | Co-expression | Roth-Zlotnik-2006 |
| ZWINT | TOP2A | 0.155119 | Co-expression | Roth-Zlotnik-2006 |
| ASPM | AURKA | 0.065079 | Co-expression | Smirnov-Cheung-2009 |
| ASPM | AURKB | 0.044751 | Co-expression | Smirnov-Cheung-2009 |
| ASPM | BUB1B | 0.042222 | Co-expression | Smirnov-Cheung-2009 |
| ASPM | CCNA2 | 0.064444 | Co-expression | Smirnov-Cheung-2009 |
| ASPM | CCNB1 | 0.041277 | Co-expression | Smirnov-Cheung-2009 |
| ASPM | CCNF | 0.075399 | Co-expression | Smirnov-Cheung-2009 |
| ASPM | CDC25C | 0.061923 | Co-expression | Smirnov-Cheung-2009 |
| ASPM | CDCA3 | 0.051716 | Co-expression | Smirnov-Cheung-2009 |
| ASPM | CENPE | 0.052097 | Co-expression | Smirnov-Cheung-2009 |
| ASPM | CENPF | 0.095868 | Co-expression | Smirnov-Cheung-2009 |
| ASPM | DEPDC1 | 0.040975 | Co-expression | Smirnov-Cheung-2009 |
| ASPM | HMMR | 0.061094 | Co-expression | Smirnov-Cheung-2009 |
| ASPM | KIF11 | 0.043881 | Co-expression | Smirnov-Cheung-2009 |
| ASPM | KIF20A | 0.054288 | Co-expression | Smirnov-Cheung-2009 |
| ASPM | KIF23 | 0.061854 | Co-expression | Smirnov-Cheung-2009 |
| ASPM | KIF4A | 0.076184 | Co-expression | Smirnov-Cheung-2009 |
| ASPM | MKI67 | 0.073153 | Co-expression | Smirnov-Cheung-2009 |
| ASPM | NDC80 | 0.050688 | Co-expression | Smirnov-Cheung-2009 |
| ASPM | NEK2 | 0.05765 | Co-expression | Smirnov-Cheung-2009 |
| ASPM | PRC1 | 0.04583 | Co-expression | Smirnov-Cheung-2009 |
| ASPM | SMC4 | 0.091727 | Co-expression | Smirnov-Cheung-2009 |
| ASPM | TOP2A | 0.05662 | Co-expression | Smirnov-Cheung-2009 |
| AURKA | BUB1B | 0.060053 | Co-expression | Smirnov-Cheung-2009 |
| AURKA | CCNB1 | 0.060381 | Co-expression | Smirnov-Cheung-2009 |
| AURKA | CCNF | 0.154771 | Co-expression | Smirnov-Cheung-2009 |
| AURKA | CENPE | 0.078328 | Co-expression | Smirnov-Cheung-2009 |
| AURKA | CENPF | 0.129619 | Co-expression | Smirnov-Cheung-2009 |
| AURKA | KIF20A | 0.106506 | Co-expression | Smirnov-Cheung-2009 |
| AURKA | KIF23 | 0.097523 | Co-expression | Smirnov-Cheung-2009 |
| AURKA | NDC80 | 0.078852 | Co-expression | Smirnov-Cheung-2009 |
| AURKA | NEK2 | 0.087645 | Co-expression | Smirnov-Cheung-2009 |
| AURKA | PRC1 | 0.06987 | Co-expression | Smirnov-Cheung-2009 |
| AURKB | AURKA | 0.068141 | Co-expression | Smirnov-Cheung-2009 |
| AURKB | BUB1B | 0.040308 | Co-expression | Smirnov-Cheung-2009 |
| AURKB | CCNA2 | 0.069522 | Co-expression | Smirnov-Cheung-2009 |
| AURKB | CCNB1 | 0.038522 | Co-expression | Smirnov-Cheung-2009 |
| AURKB | CCNF | 0.09812 | Co-expression | Smirnov-Cheung-2009 |
| AURKB | CENPE | 0.048119 | Co-expression | Smirnov-Cheung-2009 |
| AURKB | CENPF | 0.089996 | Co-expression | Smirnov-Cheung-2009 |
| AURKB | KIF20A | 0.055839 | Co-expression | Smirnov-Cheung-2009 |
| AURKB | KIF23 | 0.062348 | Co-expression | Smirnov-Cheung-2009 |
| AURKB | MKI67 | 0.087931 | Co-expression | Smirnov-Cheung-2009 |
| AURKB | NDC80 | 0.052396 | Co-expression | Smirnov-Cheung-2009 |
| AURKB | NEK2 | 0.064413 | Co-expression | Smirnov-Cheung-2009 |
| AURKB | NUSAP1 | 0.074983 | Co-expression | Smirnov-Cheung-2009 |
| AURKB | PRC1 | 0.051267 | Co-expression | Smirnov-Cheung-2009 |
| AURKB | TOP2A | 0.056995 | Co-expression | Smirnov-Cheung-2009 |
| BUB1B | KIF20A | 0.054126 | Co-expression | Smirnov-Cheung-2009 |
| BUB1B | PRC1 | 0.046739 | Co-expression | Smirnov-Cheung-2009 |
| CCNA2 | BUB1B | 0.051741 | Co-expression | Smirnov-Cheung-2009 |
| CCNA2 | CCNB1 | 0.05344 | Co-expression | Smirnov-Cheung-2009 |
| CCNA2 | KIF23 | 0.085534 | Co-expression | Smirnov-Cheung-2009 |
| CCNA2 | NEK2 | 0.096736 | Co-expression | Smirnov-Cheung-2009 |
| CCNA2 | PRC1 | 0.060575 | Co-expression | Smirnov-Cheung-2009 |
| CCNB1 | BUB1B | 0.032197 | Co-expression | Smirnov-Cheung-2009 |
| CCNB1 | KIF20A | 0.043749 | Co-expression | Smirnov-Cheung-2009 |
| CCNB1 | NEK2 | 0.057262 | Co-expression | Smirnov-Cheung-2009 |
| CCNB1 | PRC1 | 0.035349 | Co-expression | Smirnov-Cheung-2009 |
| CCNF | BUB1B | 0.080546 | Co-expression | Smirnov-Cheung-2009 |
| CCNF | KIF20A | 0.134077 | Co-expression | Smirnov-Cheung-2009 |
| CCNF | KIF23 | 0.104639 | Co-expression | Smirnov-Cheung-2009 |
| CCNF | PRC1 | 0.09615 | Co-expression | Smirnov-Cheung-2009 |
| CDC25C | AURKA | 0.128439 | Co-expression | Smirnov-Cheung-2009 |
| CDC25C | AURKB | 0.096151 | Co-expression | Smirnov-Cheung-2009 |
| CDC25C | BUB1B | 0.063513 | Co-expression | Smirnov-Cheung-2009 |
| CDC25C | CDCA3 | 0.094628 | Co-expression | Smirnov-Cheung-2009 |
| CDC25C | DEPDC1 | 0.085918 | Co-expression | Smirnov-Cheung-2009 |
| CDC25C | KIF11 | 0.064384 | Co-expression | Smirnov-Cheung-2009 |
| CDC25C | KIF20A | 0.103247 | Co-expression | Smirnov-Cheung-2009 |
| CDC25C | KIF4A | 0.129455 | Co-expression | Smirnov-Cheung-2009 |
| CDC25C | NDC80 | 0.114988 | Co-expression | Smirnov-Cheung-2009 |
| CDC25C | NEK2 | 0.101412 | Co-expression | Smirnov-Cheung-2009 |
| CDC25C | NUSAP1 | 0.140653 | Co-expression | Smirnov-Cheung-2009 |
| CDC25C | PRC1 | 0.081735 | Co-expression | Smirnov-Cheung-2009 |
| CDC25C | TOP2A | 0.106559 | Co-expression | Smirnov-Cheung-2009 |
| CDCA3 | AURKA | 0.088574 | Co-expression | Smirnov-Cheung-2009 |
| CDCA3 | AURKB | 0.072277 | Co-expression | Smirnov-Cheung-2009 |
| CDCA3 | CCNA2 | 0.084088 | Co-expression | Smirnov-Cheung-2009 |
| CDCA3 | CCNB1 | 0.050942 | Co-expression | Smirnov-Cheung-2009 |
| CDCA3 | CCNF | 0.111055 | Co-expression | Smirnov-Cheung-2009 |
| CDCA3 | CDK1 | 0.091096 | Co-expression | Smirnov-Cheung-2009 |
| CDCA3 | CENPE | 0.058384 | Co-expression | Smirnov-Cheung-2009 |
| CDCA3 | KIF11 | 0.054226 | Co-expression | Smirnov-Cheung-2009 |
| CDCA3 | KIF20A | 0.065557 | Co-expression | Smirnov-Cheung-2009 |
| CDCA3 | KIF23 | 0.076997 | Co-expression | Smirnov-Cheung-2009 |
| CDCA3 | MKI67 | 0.094028 | Co-expression | Smirnov-Cheung-2009 |
| CDCA3 | NEK2 | 0.081111 | Co-expression | Smirnov-Cheung-2009 |
| CDCA3 | NUSAP1 | 0.074606 | Co-expression | Smirnov-Cheung-2009 |
| CDCA3 | PRC1 | 0.058096 | Co-expression | Smirnov-Cheung-2009 |
| CDCA3 | TOP2A | 0.062411 | Co-expression | Smirnov-Cheung-2009 |
| CDK1 | CCNA2 | 0.112853 | Co-expression | Smirnov-Cheung-2009 |
| CDK1 | KIF11 | 0.064223 | Co-expression | Smirnov-Cheung-2009 |
| CENPE | BUB1B | 0.041769 | Co-expression | Smirnov-Cheung-2009 |
| CENPE | CCNA2 | 0.06346 | Co-expression | Smirnov-Cheung-2009 |
| CENPE | CCNB1 | 0.049714 | Co-expression | Smirnov-Cheung-2009 |
| CENPE | CCNF | 0.085425 | Co-expression | Smirnov-Cheung-2009 |
| CENPE | CENPF | 0.108239 | Co-expression | Smirnov-Cheung-2009 |
| CENPE | KIF20A | 0.064473 | Co-expression | Smirnov-Cheung-2009 |
| CENPE | KIF23 | 0.069965 | Co-expression | Smirnov-Cheung-2009 |
| CENPE | NEK2 | 0.066307 | Co-expression | Smirnov-Cheung-2009 |
| CENPE | PRC1 | 0.046898 | Co-expression | Smirnov-Cheung-2009 |
| CENPF | BUB1B | 0.081308 | Co-expression | Smirnov-Cheung-2009 |
| CENPF | CCNB1 | 0.077675 | Co-expression | Smirnov-Cheung-2009 |
| CENPF | KIF20A | 0.131673 | Co-expression | Smirnov-Cheung-2009 |
| CENPF | PRC1 | 0.093488 | Co-expression | Smirnov-Cheung-2009 |
| DEPDC1 | AURKA | 0.067641 | Co-expression | Smirnov-Cheung-2009 |
| DEPDC1 | AURKB | 0.044933 | Co-expression | Smirnov-Cheung-2009 |
| DEPDC1 | BUB1B | 0.03326 | Co-expression | Smirnov-Cheung-2009 |
| DEPDC1 | CCNA2 | 0.066145 | Co-expression | Smirnov-Cheung-2009 |
| DEPDC1 | CCNB1 | 0.03362 | Co-expression | Smirnov-Cheung-2009 |
| DEPDC1 | CDCA3 | 0.047888 | Co-expression | Smirnov-Cheung-2009 |
| DEPDC1 | CENPE | 0.044381 | Co-expression | Smirnov-Cheung-2009 |
| DEPDC1 | CENPF | 0.07788 | Co-expression | Smirnov-Cheung-2009 |
| DEPDC1 | HMMR | 0.051091 | Co-expression | Smirnov-Cheung-2009 |
| DEPDC1 | KIF11 | 0.038098 | Co-expression | Smirnov-Cheung-2009 |
| DEPDC1 | KIF20A | 0.050346 | Co-expression | Smirnov-Cheung-2009 |
| DEPDC1 | KIF23 | 0.061012 | Co-expression | Smirnov-Cheung-2009 |
| DEPDC1 | KIF4A | 0.065775 | Co-expression | Smirnov-Cheung-2009 |
| DEPDC1 | NDC80 | 0.049676 | Co-expression | Smirnov-Cheung-2009 |
| DEPDC1 | NEK2 | 0.067713 | Co-expression | Smirnov-Cheung-2009 |
| DEPDC1 | NUSAP1 | 0.050315 | Co-expression | Smirnov-Cheung-2009 |
| DEPDC1 | PRC1 | 0.041669 | Co-expression | Smirnov-Cheung-2009 |
| DEPDC1 | TOP2A | 0.047461 | Co-expression | Smirnov-Cheung-2009 |
| HMMR | AURKA | 0.085625 | Co-expression | Smirnov-Cheung-2009 |
| HMMR | AURKB | 0.055627 | Co-expression | Smirnov-Cheung-2009 |
| HMMR | BUB1B | 0.051093 | Co-expression | Smirnov-Cheung-2009 |
| HMMR | CCNA2 | 0.075809 | Co-expression | Smirnov-Cheung-2009 |
| HMMR | CCNB1 | 0.056329 | Co-expression | Smirnov-Cheung-2009 |
| HMMR | CCNF | 0.089196 | Co-expression | Smirnov-Cheung-2009 |
| HMMR | CDCA3 | 0.072308 | Co-expression | Smirnov-Cheung-2009 |
| HMMR | CENPE | 0.065757 | Co-expression | Smirnov-Cheung-2009 |
| HMMR | CENPF | 0.103892 | Co-expression | Smirnov-Cheung-2009 |
| HMMR | KIF11 | 0.054731 | Co-expression | Smirnov-Cheung-2009 |
| HMMR | KIF20A | 0.071771 | Co-expression | Smirnov-Cheung-2009 |
| HMMR | KIF23 | 0.073321 | Co-expression | Smirnov-Cheung-2009 |
| HMMR | NDC80 | 0.063824 | Co-expression | Smirnov-Cheung-2009 |
| HMMR | NEK2 | 0.079166 | Co-expression | Smirnov-Cheung-2009 |
| HMMR | PRC1 | 0.054841 | Co-expression | Smirnov-Cheung-2009 |
| HMMR | TOP2A | 0.071382 | Co-expression | Smirnov-Cheung-2009 |
| KIF11 | AURKA | 0.054241 | Co-expression | Smirnov-Cheung-2009 |
| KIF11 | AURKB | 0.047985 | Co-expression | Smirnov-Cheung-2009 |
| KIF11 | BUB1B | 0.040654 | Co-expression | Smirnov-Cheung-2009 |
| KIF11 | CCNA2 | 0.071749 | Co-expression | Smirnov-Cheung-2009 |
| KIF11 | CCNB1 | 0.037723 | Co-expression | Smirnov-Cheung-2009 |
| KIF11 | CENPE | 0.0443 | Co-expression | Smirnov-Cheung-2009 |
| KIF11 | CENPF | 0.074019 | Co-expression | Smirnov-Cheung-2009 |
| KIF11 | KIF20A | 0.043517 | Co-expression | Smirnov-Cheung-2009 |
| KIF11 | KIF23 | 0.058274 | Co-expression | Smirnov-Cheung-2009 |
| KIF11 | MKI67 | 0.07295 | Co-expression | Smirnov-Cheung-2009 |
| KIF11 | NDC80 | 0.050488 | Co-expression | Smirnov-Cheung-2009 |
| KIF11 | NEK2 | 0.056984 | Co-expression | Smirnov-Cheung-2009 |
| KIF11 | PRC1 | 0.04294 | Co-expression | Smirnov-Cheung-2009 |
| KIF11 | TOP2A | 0.053193 | Co-expression | Smirnov-Cheung-2009 |
| KIF23 | BUB1B | 0.057212 | Co-expression | Smirnov-Cheung-2009 |
| KIF23 | CCNB1 | 0.054392 | Co-expression | Smirnov-Cheung-2009 |
| KIF23 | CENPF | 0.12779 | Co-expression | Smirnov-Cheung-2009 |
| KIF23 | KIF20A | 0.077017 | Co-expression | Smirnov-Cheung-2009 |
| KIF23 | NEK2 | 0.078453 | Co-expression | Smirnov-Cheung-2009 |
| KIF23 | PRC1 | 0.065662 | Co-expression | Smirnov-Cheung-2009 |
| KIF4A | AURKA | 0.106851 | Co-expression | Smirnov-Cheung-2009 |
| KIF4A | AURKB | 0.077256 | Co-expression | Smirnov-Cheung-2009 |
| KIF4A | BUB1B | 0.068393 | Co-expression | Smirnov-Cheung-2009 |
| KIF4A | CCNA2 | 0.104868 | Co-expression | Smirnov-Cheung-2009 |
| KIF4A | CCNB1 | 0.061342 | Co-expression | Smirnov-Cheung-2009 |
| KIF4A | CCNF | 0.139569 | Co-expression | Smirnov-Cheung-2009 |
| KIF4A | CDCA3 | 0.087275 | Co-expression | Smirnov-Cheung-2009 |
| KIF4A | CENPE | 0.086733 | Co-expression | Smirnov-Cheung-2009 |
| KIF4A | CENPF | 0.165974 | Co-expression | Smirnov-Cheung-2009 |
| KIF4A | HMMR | 0.089354 | Co-expression | Smirnov-Cheung-2009 |
| KIF4A | KIF11 | 0.074411 | Co-expression | Smirnov-Cheung-2009 |
| KIF4A | KIF20A | 0.101243 | Co-expression | Smirnov-Cheung-2009 |
| KIF4A | KIF23 | 0.106611 | Co-expression | Smirnov-Cheung-2009 |
| KIF4A | MKI67 | 0.162383 | Co-expression | Smirnov-Cheung-2009 |
| KIF4A | NDC80 | 0.096419 | Co-expression | Smirnov-Cheung-2009 |
| KIF4A | PRC1 | 0.079358 | Co-expression | Smirnov-Cheung-2009 |
| KIF4A | TOP2A | 0.104014 | Co-expression | Smirnov-Cheung-2009 |
| MKI67 | BUB1B | 0.066808 | Co-expression | Smirnov-Cheung-2009 |
| MKI67 | CCNB1 | 0.057241 | Co-expression | Smirnov-Cheung-2009 |
| MKI67 | CCNF | 0.168777 | Co-expression | Smirnov-Cheung-2009 |
| MKI67 | CENPE | 0.084969 | Co-expression | Smirnov-Cheung-2009 |
| MKI67 | CENPF | 0.191434 | Co-expression | Smirnov-Cheung-2009 |
| MKI67 | KIF20A | 0.101737 | Co-expression | Smirnov-Cheung-2009 |
| MKI67 | KIF23 | 0.098569 | Co-expression | Smirnov-Cheung-2009 |
| MKI67 | PRC1 | 0.08109 | Co-expression | Smirnov-Cheung-2009 |
| NDC80 | BUB1B | 0.057171 | Co-expression | Smirnov-Cheung-2009 |
| NDC80 | CCNF | 0.09686 | Co-expression | Smirnov-Cheung-2009 |
| NDC80 | CENPE | 0.046442 | Co-expression | Smirnov-Cheung-2009 |
| NDC80 | KIF20A | 0.076722 | Co-expression | Smirnov-Cheung-2009 |
| NDC80 | KIF23 | 0.069774 | Co-expression | Smirnov-Cheung-2009 |
| NDC80 | NUSAP1 | 0.062008 | Co-expression | Smirnov-Cheung-2009 |
| NDC80 | PRC1 | 0.057245 | Co-expression | Smirnov-Cheung-2009 |
| NUSAP1 | NEK2 | 0.078473 | Co-expression | Smirnov-Cheung-2009 |
| PRC1 | KIF20A | 0.060447 | Co-expression | Smirnov-Cheung-2009 |
| PRC1 | NEK2 | 0.052363 | Co-expression | Smirnov-Cheung-2009 |
| PRC1 | NUSAP1 | 0.05156 | Co-expression | Smirnov-Cheung-2009 |
| SMC4 | AURKB | 0.093517 | Co-expression | Smirnov-Cheung-2009 |
| SMC4 | BUB1B | 0.091836 | Co-expression | Smirnov-Cheung-2009 |
| SMC4 | CCNA2 | 0.14075 | Co-expression | Smirnov-Cheung-2009 |
| SMC4 | CENPE | 0.087399 | Co-expression | Smirnov-Cheung-2009 |
| SMC4 | HMMR | 0.101549 | Co-expression | Smirnov-Cheung-2009 |
| SMC4 | KIF11 | 0.096318 | Co-expression | Smirnov-Cheung-2009 |
| SMC4 | KIF23 | 0.105996 | Co-expression | Smirnov-Cheung-2009 |
| SMC4 | KIF4A | 0.161104 | Co-expression | Smirnov-Cheung-2009 |
| SMC4 | NDC80 | 0.124671 | Co-expression | Smirnov-Cheung-2009 |
| SMC4 | PRC1 | 0.090294 | Co-expression | Smirnov-Cheung-2009 |
| SMC4 | TOP2A | 0.1218 | Co-expression | Smirnov-Cheung-2009 |
| TOP2A | AURKA | 0.075738 | Co-expression | Smirnov-Cheung-2009 |
| TOP2A | BUB1B | 0.052254 | Co-expression | Smirnov-Cheung-2009 |
| TOP2A | CCNA2 | 0.075708 | Co-expression | Smirnov-Cheung-2009 |
| TOP2A | CCNB1 | 0.037718 | Co-expression | Smirnov-Cheung-2009 |
| TOP2A | CCNF | 0.108493 | Co-expression | Smirnov-Cheung-2009 |
| TOP2A | CENPE | 0.054728 | Co-expression | Smirnov-Cheung-2009 |
| TOP2A | CENPF | 0.113282 | Co-expression | Smirnov-Cheung-2009 |
| TOP2A | KIF20A | 0.077549 | Co-expression | Smirnov-Cheung-2009 |
| TOP2A | KIF23 | 0.069908 | Co-expression | Smirnov-Cheung-2009 |
| TOP2A | MKI67 | 0.096917 | Co-expression | Smirnov-Cheung-2009 |
| TOP2A | NDC80 | 0.082853 | Co-expression | Smirnov-Cheung-2009 |
| TOP2A | NEK2 | 0.061563 | Co-expression | Smirnov-Cheung-2009 |
| TOP2A | NUSAP1 | 0.069121 | Co-expression | Smirnov-Cheung-2009 |
| TOP2A | PRC1 | 0.057552 | Co-expression | Smirnov-Cheung-2009 |
| ZWINT | GINS1 | 0.122405 | Co-expression | Smirnov-Cheung-2009 |
| ASPM | AURKA | 0.124656 | Co-expression | Wang-Cheung-2015 |
| ASPM | AURKB | 0.103108 | Co-expression | Wang-Cheung-2015 |
| ASPM | BUB1B | 0.115803 | Co-expression | Wang-Cheung-2015 |
| ASPM | CCNA2 | 0.086222 | Co-expression | Wang-Cheung-2015 |
| ASPM | CCNB1 | 0.074216 | Co-expression | Wang-Cheung-2015 |
| ASPM | CDC25C | 0.170006 | Co-expression | Wang-Cheung-2015 |
| ASPM | CDCA3 | 0.070088 | Co-expression | Wang-Cheung-2015 |
| ASPM | CDK1 | 0.09346 | Co-expression | Wang-Cheung-2015 |
| ASPM | CENPE | 0.103799 | Co-expression | Wang-Cheung-2015 |
| ASPM | CENPF | 0.1757 | Co-expression | Wang-Cheung-2015 |
| ASPM | DEPDC1 | 0.07037 | Co-expression | Wang-Cheung-2015 |
| ASPM | HMMR | 0.102278 | Co-expression | Wang-Cheung-2015 |
| ASPM | KIF11 | 0.101923 | Co-expression | Wang-Cheung-2015 |
| ASPM | KIF20A | 0.068664 | Co-expression | Wang-Cheung-2015 |
| ASPM | KIF23 | 0.116221 | Co-expression | Wang-Cheung-2015 |
| ASPM | KIF4A | 0.057408 | Co-expression | Wang-Cheung-2015 |
| ASPM | MKI67 | 0.160589 | Co-expression | Wang-Cheung-2015 |
| ASPM | NDC80 | 0.12563 | Co-expression | Wang-Cheung-2015 |
| ASPM | NEK2 | 0.183024 | Co-expression | Wang-Cheung-2015 |
| ASPM | NUSAP1 | 0.075007 | Co-expression | Wang-Cheung-2015 |
| ASPM | PRC1 | 0.055871 | Co-expression | Wang-Cheung-2015 |
| ASPM | TOP2A | 0.087425 | Co-expression | Wang-Cheung-2015 |
| AURKA | CCNA2 | 0.136628 | Co-expression | Wang-Cheung-2015 |
| AURKA | CCNB1 | 0.108142 | Co-expression | Wang-Cheung-2015 |
| AURKA | CCNF | 0.303092 | Co-expression | Wang-Cheung-2015 |
| AURKA | NUSAP1 | 0.10045 | Co-expression | Wang-Cheung-2015 |
| AURKA | PRC1 | 0.079605 | Co-expression | Wang-Cheung-2015 |
| AURKB | CCNA2 | 0.108498 | Co-expression | Wang-Cheung-2015 |
| AURKB | CCNB1 | 0.087663 | Co-expression | Wang-Cheung-2015 |
| AURKB | CENPE | 0.117977 | Co-expression | Wang-Cheung-2015 |
| AURKB | KIF20A | 0.073242 | Co-expression | Wang-Cheung-2015 |
| AURKB | KIF23 | 0.138288 | Co-expression | Wang-Cheung-2015 |
| AURKB | MKI67 | 0.190146 | Co-expression | Wang-Cheung-2015 |
| AURKB | NDC80 | 0.125126 | Co-expression | Wang-Cheung-2015 |
| AURKB | NEK2 | 0.202667 | Co-expression | Wang-Cheung-2015 |
| AURKB | NUSAP1 | 0.086226 | Co-expression | Wang-Cheung-2015 |
| AURKB | PRC1 | 0.065664 | Co-expression | Wang-Cheung-2015 |
| AURKB | TOP2A | 0.103414 | Co-expression | Wang-Cheung-2015 |
| BUB1B | KIF20A | 0.07886 | Co-expression | Wang-Cheung-2015 |
| BUB1B | NEK2 | 0.222804 | Co-expression | Wang-Cheung-2015 |
| BUB1B | NUSAP1 | 0.092002 | Co-expression | Wang-Cheung-2015 |
| BUB1B | PRC1 | 0.067837 | Co-expression | Wang-Cheung-2015 |
| CCNA2 | BUB1B | 0.114661 | Co-expression | Wang-Cheung-2015 |
| CCNA2 | CCNB1 | 0.073469 | Co-expression | Wang-Cheung-2015 |
| CCNA2 | CCNF | 0.196222 | Co-expression | Wang-Cheung-2015 |
| CCNA2 | CENPF | 0.167096 | Co-expression | Wang-Cheung-2015 |
| CCNA2 | KIF23 | 0.117329 | Co-expression | Wang-Cheung-2015 |
| CCNA2 | NEK2 | 0.166305 | Co-expression | Wang-Cheung-2015 |
| CCNA2 | NUSAP1 | 0.070708 | Co-expression | Wang-Cheung-2015 |
| CCNA2 | PRC1 | 0.054563 | Co-expression | Wang-Cheung-2015 |
| CCNB1 | BUB1B | 0.090027 | Co-expression | Wang-Cheung-2015 |
| CCNB1 | KIF20A | 0.052714 | Co-expression | Wang-Cheung-2015 |
| CCNB1 | NEK2 | 0.137453 | Co-expression | Wang-Cheung-2015 |
| CCNB1 | NUSAP1 | 0.060115 | Co-expression | Wang-Cheung-2015 |
| CCNB1 | PRC1 | 0.046779 | Co-expression | Wang-Cheung-2015 |
| CCNF | CCNB1 | 0.137133 | Co-expression | Wang-Cheung-2015 |
| CDC25C | AURKB | 0.20377 | Co-expression | Wang-Cheung-2015 |
| CDC25C | CCNB1 | 0.142214 | Co-expression | Wang-Cheung-2015 |
| CDC25C | CDCA3 | 0.148347 | Co-expression | Wang-Cheung-2015 |
| CDC25C | CENPE | 0.193708 | Co-expression | Wang-Cheung-2015 |
| CDC25C | DEPDC1 | 0.137767 | Co-expression | Wang-Cheung-2015 |
| CDC25C | HMMR | 0.205054 | Co-expression | Wang-Cheung-2015 |
| CDC25C | KIF11 | 0.192661 | Co-expression | Wang-Cheung-2015 |
| CDC25C | KIF20A | 0.121106 | Co-expression | Wang-Cheung-2015 |
| CDC25C | KIF4A | 0.115293 | Co-expression | Wang-Cheung-2015 |
| CDC25C | NUSAP1 | 0.141496 | Co-expression | Wang-Cheung-2015 |
| CDC25C | PRC1 | 0.109525 | Co-expression | Wang-Cheung-2015 |
| CDC25C | TOP2A | 0.171916 | Co-expression | Wang-Cheung-2015 |
| CDCA3 | AURKA | 0.096352 | Co-expression | Wang-Cheung-2015 |
| CDCA3 | AURKB | 0.081841 | Co-expression | Wang-Cheung-2015 |
| CDCA3 | BUB1B | 0.085383 | Co-expression | Wang-Cheung-2015 |
| CDCA3 | CCNA2 | 0.068624 | Co-expression | Wang-Cheung-2015 |
| CDCA3 | CCNB1 | 0.058392 | Co-expression | Wang-Cheung-2015 |
| CDCA3 | CDK1 | 0.074189 | Co-expression | Wang-Cheung-2015 |
| CDCA3 | CENPE | 0.077399 | Co-expression | Wang-Cheung-2015 |
| CDCA3 | CENPF | 0.128644 | Co-expression | Wang-Cheung-2015 |
| CDCA3 | KIF11 | 0.07581 | Co-expression | Wang-Cheung-2015 |
| CDCA3 | KIF20A | 0.052864 | Co-expression | Wang-Cheung-2015 |
| CDCA3 | KIF23 | 0.089861 | Co-expression | Wang-Cheung-2015 |
| CDCA3 | MKI67 | 0.127782 | Co-expression | Wang-Cheung-2015 |
| CDCA3 | NDC80 | 0.077267 | Co-expression | Wang-Cheung-2015 |
| CDCA3 | NEK2 | 0.129634 | Co-expression | Wang-Cheung-2015 |
| CDCA3 | NUSAP1 | 0.059126 | Co-expression | Wang-Cheung-2015 |
| CDCA3 | PRC1 | 0.045402 | Co-expression | Wang-Cheung-2015 |
| CDCA3 | TOP2A | 0.068721 | Co-expression | Wang-Cheung-2015 |
| CDK1 | AURKB | 0.121619 | Co-expression | Wang-Cheung-2015 |
| CDK1 | BUB1B | 0.132226 | Co-expression | Wang-Cheung-2015 |
| CDK1 | CCNA2 | 0.103898 | Co-expression | Wang-Cheung-2015 |
| CDK1 | CCNB1 | 0.078515 | Co-expression | Wang-Cheung-2015 |
| CDK1 | CCNF | 0.198303 | Co-expression | Wang-Cheung-2015 |
| CDK1 | CENPE | 0.105314 | Co-expression | Wang-Cheung-2015 |
| CDK1 | CENPF | 0.191386 | Co-expression | Wang-Cheung-2015 |
| CDK1 | GINS1 | 0.086768 | Co-expression | Wang-Cheung-2015 |
| CDK1 | KIF11 | 0.117025 | Co-expression | Wang-Cheung-2015 |
| CDK1 | KIF23 | 0.129211 | Co-expression | Wang-Cheung-2015 |
| CDK1 | NDC80 | 0.137741 | Co-expression | Wang-Cheung-2015 |
| CDK1 | NEK2 | 0.190951 | Co-expression | Wang-Cheung-2015 |
| CDK1 | NUSAP1 | 0.076274 | Co-expression | Wang-Cheung-2015 |
| CDK1 | PRC1 | 0.057121 | Co-expression | Wang-Cheung-2015 |
| CDK1 | TOP2A | 0.097186 | Co-expression | Wang-Cheung-2015 |
| CENPE | BUB1B | 0.124477 | Co-expression | Wang-Cheung-2015 |
| CENPE | CCNA2 | 0.091733 | Co-expression | Wang-Cheung-2015 |
| CENPE | CCNB1 | 0.079443 | Co-expression | Wang-Cheung-2015 |
| CENPE | CENPF | 0.182564 | Co-expression | Wang-Cheung-2015 |
| CENPE | KIF20A | 0.072586 | Co-expression | Wang-Cheung-2015 |
| CENPE | KIF23 | 0.124471 | Co-expression | Wang-Cheung-2015 |
| CENPE | NEK2 | 0.187815 | Co-expression | Wang-Cheung-2015 |
| CENPE | NUSAP1 | 0.08071 | Co-expression | Wang-Cheung-2015 |
| CENPE | PRC1 | 0.060112 | Co-expression | Wang-Cheung-2015 |
| CENPF | BUB1B | 0.232819 | Co-expression | Wang-Cheung-2015 |
| CENPF | CCNB1 | 0.135231 | Co-expression | Wang-Cheung-2015 |
| CENPF | KIF20A | 0.117696 | Co-expression | Wang-Cheung-2015 |
| CENPF | NUSAP1 | 0.136166 | Co-expression | Wang-Cheung-2015 |
| CENPF | PRC1 | 0.09819 | Co-expression | Wang-Cheung-2015 |
| DEPDC1 | AURKA | 0.107085 | Co-expression | Wang-Cheung-2015 |
| DEPDC1 | AURKB | 0.086621 | Co-expression | Wang-Cheung-2015 |
| DEPDC1 | BUB1B | 0.084684 | Co-expression | Wang-Cheung-2015 |
| DEPDC1 | CCNA2 | 0.073611 | Co-expression | Wang-Cheung-2015 |
| DEPDC1 | CCNB1 | 0.060106 | Co-expression | Wang-Cheung-2015 |
| DEPDC1 | CCNF | 0.142715 | Co-expression | Wang-Cheung-2015 |
| DEPDC1 | CDCA3 | 0.056118 | Co-expression | Wang-Cheung-2015 |
| DEPDC1 | CDK1 | 0.076449 | Co-expression | Wang-Cheung-2015 |
| DEPDC1 | CENPE | 0.076901 | Co-expression | Wang-Cheung-2015 |
| DEPDC1 | CENPF | 0.136827 | Co-expression | Wang-Cheung-2015 |
| DEPDC1 | HMMR | 0.083192 | Co-expression | Wang-Cheung-2015 |
| DEPDC1 | KIF11 | 0.074257 | Co-expression | Wang-Cheung-2015 |
| DEPDC1 | KIF20A | 0.050264 | Co-expression | Wang-Cheung-2015 |
| DEPDC1 | KIF23 | 0.093088 | Co-expression | Wang-Cheung-2015 |
| DEPDC1 | KIF4A | 0.047804 | Co-expression | Wang-Cheung-2015 |
| DEPDC1 | MKI67 | 0.126959 | Co-expression | Wang-Cheung-2015 |
| DEPDC1 | NEK2 | 0.138359 | Co-expression | Wang-Cheung-2015 |
| DEPDC1 | NUSAP1 | 0.058614 | Co-expression | Wang-Cheung-2015 |
| DEPDC1 | PRC1 | 0.045455 | Co-expression | Wang-Cheung-2015 |
| DEPDC1 | TOP2A | 0.069512 | Co-expression | Wang-Cheung-2015 |
| DEPDC1 | ZWINT | 0.059809 | Co-expression | Wang-Cheung-2015 |
| HMMR | AURKB | 0.130623 | Co-expression | Wang-Cheung-2015 |
| HMMR | BUB1B | 0.126324 | Co-expression | Wang-Cheung-2015 |
| HMMR | CCNA2 | 0.099579 | Co-expression | Wang-Cheung-2015 |
| HMMR | CCNB1 | 0.083924 | Co-expression | Wang-Cheung-2015 |
| HMMR | CDCA3 | 0.079959 | Co-expression | Wang-Cheung-2015 |
| HMMR | CDK1 | 0.114873 | Co-expression | Wang-Cheung-2015 |
| HMMR | CENPE | 0.115852 | Co-expression | Wang-Cheung-2015 |
| HMMR | CENPF | 0.204366 | Co-expression | Wang-Cheung-2015 |
| HMMR | KIF11 | 0.114834 | Co-expression | Wang-Cheung-2015 |
| HMMR | KIF20A | 0.074484 | Co-expression | Wang-Cheung-2015 |
| HMMR | KIF23 | 0.136373 | Co-expression | Wang-Cheung-2015 |
| HMMR | MKI67 | 0.181556 | Co-expression | Wang-Cheung-2015 |
| HMMR | NDC80 | 0.127876 | Co-expression | Wang-Cheung-2015 |
| HMMR | NEK2 | 0.211718 | Co-expression | Wang-Cheung-2015 |
| HMMR | NUSAP1 | 0.084132 | Co-expression | Wang-Cheung-2015 |
| HMMR | PRC1 | 0.063469 | Co-expression | Wang-Cheung-2015 |
| HMMR | TOP2A | 0.10273 | Co-expression | Wang-Cheung-2015 |
| KIF11 | AURKB | 0.119288 | Co-expression | Wang-Cheung-2015 |
| KIF11 | BUB1B | 0.133638 | Co-expression | Wang-Cheung-2015 |
| KIF11 | CCNA2 | 0.098767 | Co-expression | Wang-Cheung-2015 |
| KIF11 | CCNB1 | 0.078343 | Co-expression | Wang-Cheung-2015 |
| KIF11 | CENPE | 0.112745 | Co-expression | Wang-Cheung-2015 |
| KIF11 | CENPF | 0.191569 | Co-expression | Wang-Cheung-2015 |
| KIF11 | KIF20A | 0.067277 | Co-expression | Wang-Cheung-2015 |
| KIF11 | KIF23 | 0.129684 | Co-expression | Wang-Cheung-2015 |
| KIF11 | NDC80 | 0.158685 | Co-expression | Wang-Cheung-2015 |
| KIF11 | NEK2 | 0.198263 | Co-expression | Wang-Cheung-2015 |
| KIF11 | NUSAP1 | 0.078009 | Co-expression | Wang-Cheung-2015 |
| KIF11 | PRC1 | 0.058176 | Co-expression | Wang-Cheung-2015 |
| KIF11 | TOP2A | 0.098471 | Co-expression | Wang-Cheung-2015 |
| KIF20A | NEK2 | 0.12311 | Co-expression | Wang-Cheung-2015 |
| KIF23 | BUB1B | 0.150405 | Co-expression | Wang-Cheung-2015 |
| KIF23 | CCNB1 | 0.093322 | Co-expression | Wang-Cheung-2015 |
| KIF23 | CENPF | 0.230476 | Co-expression | Wang-Cheung-2015 |
| KIF23 | KIF20A | 0.078212 | Co-expression | Wang-Cheung-2015 |
| KIF23 | NEK2 | 0.241799 | Co-expression | Wang-Cheung-2015 |
| KIF23 | NUSAP1 | 0.093421 | Co-expression | Wang-Cheung-2015 |
| KIF23 | PRC1 | 0.069772 | Co-expression | Wang-Cheung-2015 |
| KIF4A | AURKA | 0.082557 | Co-expression | Wang-Cheung-2015 |
| KIF4A | AURKB | 0.069026 | Co-expression | Wang-Cheung-2015 |
| KIF4A | BUB1B | 0.070452 | Co-expression | Wang-Cheung-2015 |
| KIF4A | CCNA2 | 0.057653 | Co-expression | Wang-Cheung-2015 |
| KIF4A | CCNB1 | 0.048204 | Co-expression | Wang-Cheung-2015 |
| KIF4A | CCNF | 0.104735 | Co-expression | Wang-Cheung-2015 |
| KIF4A | CDCA3 | 0.046972 | Co-expression | Wang-Cheung-2015 |
| KIF4A | CDK1 | 0.061781 | Co-expression | Wang-Cheung-2015 |
| KIF4A | CENPE | 0.062678 | Co-expression | Wang-Cheung-2015 |
| KIF4A | CENPF | 0.108224 | Co-expression | Wang-Cheung-2015 |
| KIF4A | HMMR | 0.066693 | Co-expression | Wang-Cheung-2015 |
| KIF4A | KIF11 | 0.061342 | Co-expression | Wang-Cheung-2015 |
| KIF4A | KIF20A | 0.042462 | Co-expression | Wang-Cheung-2015 |
| KIF4A | KIF23 | 0.073806 | Co-expression | Wang-Cheung-2015 |
| KIF4A | MKI67 | 0.10545 | Co-expression | Wang-Cheung-2015 |
| KIF4A | NEK2 | 0.11179 | Co-expression | Wang-Cheung-2015 |
| KIF4A | NUSAP1 | 0.048227 | Co-expression | Wang-Cheung-2015 |
| KIF4A | PRC1 | 0.037675 | Co-expression | Wang-Cheung-2015 |
| KIF4A | TOP2A | 0.056122 | Co-expression | Wang-Cheung-2015 |
| KIF4A | ZWINT | 0.049119 | Co-expression | Wang-Cheung-2015 |
| MKI67 | BUB1B | 0.200681 | Co-expression | Wang-Cheung-2015 |
| MKI67 | CCNA2 | 0.157682 | Co-expression | Wang-Cheung-2015 |
| MKI67 | CCNB1 | 0.132372 | Co-expression | Wang-Cheung-2015 |
| MKI67 | CENPE | 0.176019 | Co-expression | Wang-Cheung-2015 |
| MKI67 | KIF20A | 0.118855 | Co-expression | Wang-Cheung-2015 |
| MKI67 | NEK2 | 0.307782 | Co-expression | Wang-Cheung-2015 |
| MKI67 | NUSAP1 | 0.132609 | Co-expression | Wang-Cheung-2015 |
| MKI67 | PRC1 | 0.103412 | Co-expression | Wang-Cheung-2015 |
| NDC80 | BUB1B | 0.168407 | Co-expression | Wang-Cheung-2015 |
| NDC80 | CCNA2 | 0.096917 | Co-expression | Wang-Cheung-2015 |
| NDC80 | CCNB1 | 0.07785 | Co-expression | Wang-Cheung-2015 |
| NDC80 | CENPE | 0.1337 | Co-expression | Wang-Cheung-2015 |
| NDC80 | KIF20A | 0.072503 | Co-expression | Wang-Cheung-2015 |
| NDC80 | KIF23 | 0.163528 | Co-expression | Wang-Cheung-2015 |
| NDC80 | NUSAP1 | 0.081089 | Co-expression | Wang-Cheung-2015 |
| NUSAP1 | KIF20A | 0.05534 | Co-expression | Wang-Cheung-2015 |
| NUSAP1 | NEK2 | 0.142261 | Co-expression | Wang-Cheung-2015 |
| PRC1 | KIF20A | 0.042984 | Co-expression | Wang-Cheung-2015 |
| PRC1 | NEK2 | 0.108508 | Co-expression | Wang-Cheung-2015 |
| PRC1 | NUSAP1 | 0.047239 | Co-expression | Wang-Cheung-2015 |
| TOP2A | BUB1B | 0.11078 | Co-expression | Wang-Cheung-2015 |
| TOP2A | CCNA2 | 0.085446 | Co-expression | Wang-Cheung-2015 |
| TOP2A | CCNB1 | 0.070596 | Co-expression | Wang-Cheung-2015 |
| TOP2A | CENPE | 0.097157 | Co-expression | Wang-Cheung-2015 |
| TOP2A | CENPF | 0.167356 | Co-expression | Wang-Cheung-2015 |
| TOP2A | KIF20A | 0.06228 | Co-expression | Wang-Cheung-2015 |
| TOP2A | KIF23 | 0.111581 | Co-expression | Wang-Cheung-2015 |
| TOP2A | MKI67 | 0.153413 | Co-expression | Wang-Cheung-2015 |
| TOP2A | NDC80 | 0.11324 | Co-expression | Wang-Cheung-2015 |
| TOP2A | NEK2 | 0.169453 | Co-expression | Wang-Cheung-2015 |
| TOP2A | NUSAP1 | 0.070899 | Co-expression | Wang-Cheung-2015 |
| TOP2A | PRC1 | 0.053708 | Co-expression | Wang-Cheung-2015 |
| ZWINT | AURKA | 0.110402 | Co-expression | Wang-Cheung-2015 |
| ZWINT | AURKB | 0.089124 | Co-expression | Wang-Cheung-2015 |
| ZWINT | BUB1B | 0.098452 | Co-expression | Wang-Cheung-2015 |
| ZWINT | CCNA2 | 0.080102 | Co-expression | Wang-Cheung-2015 |
| ZWINT | CCNB1 | 0.061075 | Co-expression | Wang-Cheung-2015 |
| ZWINT | CCNF | 0.161879 | Co-expression | Wang-Cheung-2015 |
| ZWINT | CDCA3 | 0.058337 | Co-expression | Wang-Cheung-2015 |
| ZWINT | CDK1 | 0.088341 | Co-expression | Wang-Cheung-2015 |
| ZWINT | GINS1 | 0.072473 | Co-expression | Wang-Cheung-2015 |
| ZWINT | HMMR | 0.082728 | Co-expression | Wang-Cheung-2015 |
| ZWINT | KIF11 | 0.081346 | Co-expression | Wang-Cheung-2015 |
| ZWINT | KIF23 | 0.096259 | Co-expression | Wang-Cheung-2015 |
| ZWINT | MKI67 | 0.138382 | Co-expression | Wang-Cheung-2015 |
| ZWINT | NEK2 | 0.141546 | Co-expression | Wang-Cheung-2015 |
| ZWINT | NUSAP1 | 0.060247 | Co-expression | Wang-Cheung-2015 |
| ZWINT | PRC1 | 0.048635 | Co-expression | Wang-Cheung-2015 |
| ZWINT | TOP2A | 0.072139 | Co-expression | Wang-Cheung-2015 |
| AURKA | BUB1B | 0.01206 | Co-expression | Wang-Maris-2006 |
| AURKA | CCNA2 | 0.023187 | Co-expression | Wang-Maris-2006 |
| AURKA | CCNB1 | 0.017445 | Co-expression | Wang-Maris-2006 |
| AURKA | GINS1 | 0.015135 | Co-expression | Wang-Maris-2006 |
| AURKA | KIF23 | 0.049862 | Co-expression | Wang-Maris-2006 |
| AURKB | BUB1B | 0.007535 | Co-expression | Wang-Maris-2006 |
| AURKB | CCNA2 | 0.011488 | Co-expression | Wang-Maris-2006 |
| AURKB | CCNB1 | 0.009016 | Co-expression | Wang-Maris-2006 |
| AURKB | CENPE | 0.00965 | Co-expression | Wang-Maris-2006 |
| AURKB | CENPF | 0.008125 | Co-expression | Wang-Maris-2006 |
| AURKB | KIF23 | 0.014106 | Co-expression | Wang-Maris-2006 |
| AURKB | MKI67 | 0.018724 | Co-expression | Wang-Maris-2006 |
| AURKB | NDC80 | 0.011142 | Co-expression | Wang-Maris-2006 |
| AURKB | TOP2A | 0.011772 | Co-expression | Wang-Maris-2006 |
| BUB1B | GINS1 | 0.005783 | Co-expression | Wang-Maris-2006 |
| CCNA2 | BUB1B | 0.009023 | Co-expression | Wang-Maris-2006 |
| CCNA2 | CCNB1 | 0.01095 | Co-expression | Wang-Maris-2006 |
| CCNA2 | NEK2 | 0.021105 | Co-expression | Wang-Maris-2006 |
| CCNB1 | BUB1B | 0.006764 | Co-expression | Wang-Maris-2006 |
| CCNB1 | GINS1 | 0.00728 | Co-expression | Wang-Maris-2006 |
| CCNB1 | NEK2 | 0.012083 | Co-expression | Wang-Maris-2006 |
| CCNF | KIF23 | 0.042072 | Co-expression | Wang-Maris-2006 |
| CDC25C | AURKA | 0.031628 | Co-expression | Wang-Maris-2006 |
| CDC25C | AURKB | 0.015242 | Co-expression | Wang-Maris-2006 |
| CDC25C | BUB1B | 0.011438 | Co-expression | Wang-Maris-2006 |
| CDC25C | CCNA2 | 0.020408 | Co-expression | Wang-Maris-2006 |
| CDC25C | CCNB1 | 0.015019 | Co-expression | Wang-Maris-2006 |
| CDC25C | CCNF | 0.031632 | Co-expression | Wang-Maris-2006 |
| CDC25C | CDK1 | 0.017102 | Co-expression | Wang-Maris-2006 |
| CDC25C | GINS1 | 0.011862 | Co-expression | Wang-Maris-2006 |
| CDC25C | HMMR | 0.02026 | Co-expression | Wang-Maris-2006 |
| CDC25C | KIF11 | 0.013097 | Co-expression | Wang-Maris-2006 |
| CDC25C | KIF23 | 0.028129 | Co-expression | Wang-Maris-2006 |
| CDC25C | MKI67 | 0.031487 | Co-expression | Wang-Maris-2006 |
| CDC25C | NDC80 | 0.018284 | Co-expression | Wang-Maris-2006 |
| CDC25C | NEK2 | 0.023981 | Co-expression | Wang-Maris-2006 |
| CDC25C | ZWINT | 0.016923 | Co-expression | Wang-Maris-2006 |
| CDK1 | BUB1B | 0.007981 | Co-expression | Wang-Maris-2006 |
| CDK1 | CCNA2 | 0.014461 | Co-expression | Wang-Maris-2006 |
| CDK1 | CCNB1 | 0.009108 | Co-expression | Wang-Maris-2006 |
| CDK1 | CENPE | 0.011482 | Co-expression | Wang-Maris-2006 |
| CDK1 | GINS1 | 0.008129 | Co-expression | Wang-Maris-2006 |
| CDK1 | KIF11 | 0.010112 | Co-expression | Wang-Maris-2006 |
| CDK1 | MKI67 | 0.020711 | Co-expression | Wang-Maris-2006 |
| CDK1 | NDC80 | 0.013839 | Co-expression | Wang-Maris-2006 |
| CDK1 | NEK2 | 0.016042 | Co-expression | Wang-Maris-2006 |
| CDK1 | TOP2A | 0.014291 | Co-expression | Wang-Maris-2006 |
| CENPE | BUB1B | 0.008163 | Co-expression | Wang-Maris-2006 |
| CENPE | CCNA2 | 0.012192 | Co-expression | Wang-Maris-2006 |
| CENPE | CCNB1 | 0.008905 | Co-expression | Wang-Maris-2006 |
| CENPE | CENPF | 0.008157 | Co-expression | Wang-Maris-2006 |
| CENPE | GINS1 | 0.007795 | Co-expression | Wang-Maris-2006 |
| CENPF | BUB1B | 0.006127 | Co-expression | Wang-Maris-2006 |
| CENPF | CCNB1 | 0.007191 | Co-expression | Wang-Maris-2006 |
| CENPF | GINS1 | 0.006428 | Co-expression | Wang-Maris-2006 |
| CENPF | NEK2 | 0.010082 | Co-expression | Wang-Maris-2006 |
| HMMR | AURKA | 0.022034 | Co-expression | Wang-Maris-2006 |
| HMMR | AURKB | 0.011365 | Co-expression | Wang-Maris-2006 |
| HMMR | BUB1B | 0.008986 | Co-expression | Wang-Maris-2006 |
| HMMR | CCNA2 | 0.015904 | Co-expression | Wang-Maris-2006 |
| HMMR | CCNB1 | 0.011502 | Co-expression | Wang-Maris-2006 |
| HMMR | CDK1 | 0.014147 | Co-expression | Wang-Maris-2006 |
| HMMR | CENPE | 0.012131 | Co-expression | Wang-Maris-2006 |
| HMMR | CENPF | 0.009177 | Co-expression | Wang-Maris-2006 |
| HMMR | GINS1 | 0.0098 | Co-expression | Wang-Maris-2006 |
| HMMR | KIF11 | 0.010834 | Co-expression | Wang-Maris-2006 |
| HMMR | MKI67 | 0.023864 | Co-expression | Wang-Maris-2006 |
| HMMR | NDC80 | 0.016397 | Co-expression | Wang-Maris-2006 |
| HMMR | NEK2 | 0.017708 | Co-expression | Wang-Maris-2006 |
| HMMR | TOP2A | 0.016347 | Co-expression | Wang-Maris-2006 |
| KIF11 | AURKB | 0.008226 | Co-expression | Wang-Maris-2006 |
| KIF11 | BUB1B | 0.006667 | Co-expression | Wang-Maris-2006 |
| KIF11 | CCNB1 | 0.00747 | Co-expression | Wang-Maris-2006 |
| KIF11 | CENPE | 0.009354 | Co-expression | Wang-Maris-2006 |
| KIF11 | CENPF | 0.006831 | Co-expression | Wang-Maris-2006 |
| KIF11 | GINS1 | 0.007453 | Co-expression | Wang-Maris-2006 |
| KIF11 | MKI67 | 0.018015 | Co-expression | Wang-Maris-2006 |
| KIF11 | NDC80 | 0.01148 | Co-expression | Wang-Maris-2006 |
| KIF11 | TOP2A | 0.01069 | Co-expression | Wang-Maris-2006 |
| KIF23 | BUB1B | 0.010877 | Co-expression | Wang-Maris-2006 |
| KIF23 | CCNB1 | 0.015194 | Co-expression | Wang-Maris-2006 |
| KIF23 | CENPF | 0.013094 | Co-expression | Wang-Maris-2006 |
| KIF23 | GINS1 | 0.01207 | Co-expression | Wang-Maris-2006 |
| MKI67 | BUB1B | 0.014542 | Co-expression | Wang-Maris-2006 |
| MKI67 | CCNA2 | 0.023381 | Co-expression | Wang-Maris-2006 |
| MKI67 | CCNB1 | 0.017173 | Co-expression | Wang-Maris-2006 |
| MKI67 | CENPE | 0.018373 | Co-expression | Wang-Maris-2006 |
| MKI67 | CENPF | 0.015405 | Co-expression | Wang-Maris-2006 |
| MKI67 | GINS1 | 0.01406 | Co-expression | Wang-Maris-2006 |
| MKI67 | KIF23 | 0.032641 | Co-expression | Wang-Maris-2006 |
| NDC80 | BUB1B | 0.008816 | Co-expression | Wang-Maris-2006 |
| NDC80 | CCNA2 | 0.014158 | Co-expression | Wang-Maris-2006 |
| NDC80 | CCNB1 | 0.010517 | Co-expression | Wang-Maris-2006 |
| NDC80 | CENPE | 0.012776 | Co-expression | Wang-Maris-2006 |
| NDC80 | GINS1 | 0.010156 | Co-expression | Wang-Maris-2006 |
| SMC4 | AURKB | 0.011315 | Co-expression | Wang-Maris-2006 |
| SMC4 | BUB1B | 0.009567 | Co-expression | Wang-Maris-2006 |
| SMC4 | CCNA2 | 0.017454 | Co-expression | Wang-Maris-2006 |
| SMC4 | CDK1 | 0.01486 | Co-expression | Wang-Maris-2006 |
| SMC4 | CENPE | 0.01337 | Co-expression | Wang-Maris-2006 |
| SMC4 | HMMR | 0.01533 | Co-expression | Wang-Maris-2006 |
| SMC4 | KIF11 | 0.01016 | Co-expression | Wang-Maris-2006 |
| SMC4 | NDC80 | 0.014332 | Co-expression | Wang-Maris-2006 |
| SMC4 | NEK2 | 0.020209 | Co-expression | Wang-Maris-2006 |
| SMC4 | TOP2A | 0.017157 | Co-expression | Wang-Maris-2006 |
| SMC4 | ZWINT | 0.013292 | Co-expression | Wang-Maris-2006 |
| TOP2A | BUB1B | 0.008972 | Co-expression | Wang-Maris-2006 |
| TOP2A | CCNA2 | 0.017037 | Co-expression | Wang-Maris-2006 |
| TOP2A | CCNB1 | 0.01022 | Co-expression | Wang-Maris-2006 |
| TOP2A | CENPE | 0.012219 | Co-expression | Wang-Maris-2006 |
| TOP2A | CENPF | 0.009117 | Co-expression | Wang-Maris-2006 |
| TOP2A | MKI67 | 0.02274 | Co-expression | Wang-Maris-2006 |
| TOP2A | NDC80 | 0.015748 | Co-expression | Wang-Maris-2006 |
| TOP2A | NEK2 | 0.019829 | Co-expression | Wang-Maris-2006 |
| ZWINT | AURKA | 0.019659 | Co-expression | Wang-Maris-2006 |
| ZWINT | BUB1B | 0.007471 | Co-expression | Wang-Maris-2006 |
| ZWINT | CCNA2 | 0.013278 | Co-expression | Wang-Maris-2006 |
| ZWINT | CDK1 | 0.012573 | Co-expression | Wang-Maris-2006 |
| ZWINT | CENPE | 0.011213 | Co-expression | Wang-Maris-2006 |
| ZWINT | GINS1 | 0.008435 | Co-expression | Wang-Maris-2006 |
| ZWINT | HMMR | 0.013446 | Co-expression | Wang-Maris-2006 |
| ZWINT | KIF11 | 0.010179 | Co-expression | Wang-Maris-2006 |
| ZWINT | KIF23 | 0.015833 | Co-expression | Wang-Maris-2006 |
| ZWINT | MKI67 | 0.019578 | Co-expression | Wang-Maris-2006 |
| ZWINT | NDC80 | 0.013215 | Co-expression | Wang-Maris-2006 |
| ZWINT | NEK2 | 0.013996 | Co-expression | Wang-Maris-2006 |
| ZWINT | TOP2A | 0.013025 | Co-expression | Wang-Maris-2006 |
| BUB1B | GINS1 | 0.003599 | Co-expression | Wu-Garvey-2007 |
| CDK1 | BUB1B | 0.00572 | Co-expression | Wu-Garvey-2007 |
| CENPE | KIF23 | 0.006567 | Co-expression | Wu-Garvey-2007 |
| HMMR | BUB1B | 0.002793 | Co-expression | Wu-Garvey-2007 |
| KIF11 | NDC80 | 0.003239 | Co-expression | Wu-Garvey-2007 |
| KIF23 | GINS1 | 0.005445 | Co-expression | Wu-Garvey-2007 |
| NDC80 | CCNB1 | 0.006576 | Co-expression | Wu-Garvey-2007 |
| ZWINT | BUB1B | 0.002852 | Co-expression | Wu-Garvey-2007 |
| AURKA | GINS1 | 0.017712 | Co-localization | Johnson-Shoemaker-2003 |
| AURKA | KIF23 | 0.038676 | Co-localization | Johnson-Shoemaker-2003 |
| AURKA | NDC80 | 0.068374 | Co-localization | Johnson-Shoemaker-2003 |
| AURKA | NEK2 | 0.051821 | Co-localization | Johnson-Shoemaker-2003 |
| AURKA | PRC1 | 0.040646 | Co-localization | Johnson-Shoemaker-2003 |
| AURKB | CCNA2 | 0.04439 | Co-localization | Johnson-Shoemaker-2003 |
| AURKB | CCNB1 | 0.03973 | Co-localization | Johnson-Shoemaker-2003 |
| AURKB | GINS1 | 0.04549 | Co-localization | Johnson-Shoemaker-2003 |
| BUB1B | NUSAP1 | 0.047577 | Co-localization | Johnson-Shoemaker-2003 |
| CCNA2 | BUB1B | 0.039647 | Co-localization | Johnson-Shoemaker-2003 |
| CCNA2 | CCNB1 | 0.011746 | Co-localization | Johnson-Shoemaker-2003 |
| CCNA2 | CCNF | 0.043613 | Co-localization | Johnson-Shoemaker-2003 |
| CCNA2 | CENPF | 0.042498 | Co-localization | Johnson-Shoemaker-2003 |
| CCNA2 | GINS1 | 0.012646 | Co-localization | Johnson-Shoemaker-2003 |
| CCNA2 | NUSAP1 | 0.013968 | Co-localization | Johnson-Shoemaker-2003 |
| CCNB1 | BUB1B | 0.036334 | Co-localization | Johnson-Shoemaker-2003 |
| CCNB1 | PRC1 | 0.024074 | Co-localization | Johnson-Shoemaker-2003 |
| CCNF | CCNB1 | 0.036468 | Co-localization | Johnson-Shoemaker-2003 |
| CCNF | CENPF | 0.142149 | Co-localization | Johnson-Shoemaker-2003 |
| CCNF | GINS1 | 0.043149 | Co-localization | Johnson-Shoemaker-2003 |
| CCNF | NUSAP1 | 0.051912 | Co-localization | Johnson-Shoemaker-2003 |
| CDC25C | AURKA | 0.055914 | Co-localization | Johnson-Shoemaker-2003 |
| CDC25C | GINS1 | 0.042917 | Co-localization | Johnson-Shoemaker-2003 |
| CDC25C | HMMR | 0.055156 | Co-localization | Johnson-Shoemaker-2003 |
| CDC25C | NUSAP1 | 0.044511 | Co-localization | Johnson-Shoemaker-2003 |
| CDC25C | ZWINT | 0.044308 | Co-localization | Johnson-Shoemaker-2003 |
| CDCA3 | AURKA | 0.068547 | Co-localization | Johnson-Shoemaker-2003 |
| CDCA3 | CCNA2 | 0.0439 | Co-localization | Johnson-Shoemaker-2003 |
| CDCA3 | CCNB1 | 0.04267 | Co-localization | Johnson-Shoemaker-2003 |
| CDCA3 | NUSAP1 | 0.05244 | Co-localization | Johnson-Shoemaker-2003 |
| CDCA3 | PRC1 | 0.121991 | Co-localization | Johnson-Shoemaker-2003 |
| CDK1 | BUB1B | 0.063687 | Co-localization | Johnson-Shoemaker-2003 |
| CDK1 | CENPE | 0.073697 | Co-localization | Johnson-Shoemaker-2003 |
| CDK1 | TOP2A | 0.049598 | Co-localization | Johnson-Shoemaker-2003 |
| CENPE | BUB1B | 0.160783 | Co-localization | Johnson-Shoemaker-2003 |
| CENPE | CCNA2 | 0.049308 | Co-localization | Johnson-Shoemaker-2003 |
| CENPE | CCNB1 | 0.04533 | Co-localization | Johnson-Shoemaker-2003 |
| CENPE | CENPF | 0.172221 | Co-localization | Johnson-Shoemaker-2003 |
| CENPE | NUSAP1 | 0.05485 | Co-localization | Johnson-Shoemaker-2003 |
| CENPF | CCNB1 | 0.039814 | Co-localization | Johnson-Shoemaker-2003 |
| CENPF | NUSAP1 | 0.04598 | Co-localization | Johnson-Shoemaker-2003 |
| DEPDC1 | AURKA | 0.037678 | Co-localization | Johnson-Shoemaker-2003 |
| DEPDC1 | HMMR | 0.039706 | Co-localization | Johnson-Shoemaker-2003 |
| DEPDC1 | KIF11 | 0.060704 | Co-localization | Johnson-Shoemaker-2003 |
| DEPDC1 | KIF23 | 0.072323 | Co-localization | Johnson-Shoemaker-2003 |
| DEPDC1 | KIF4A | 0.095822 | Co-localization | Johnson-Shoemaker-2003 |
| DEPDC1 | NEK2 | 0.093179 | Co-localization | Johnson-Shoemaker-2003 |
| DEPDC1 | PRC1 | 0.064263 | Co-localization | Johnson-Shoemaker-2003 |
| GINS1 | NUSAP1 | 0.014653 | Co-localization | Johnson-Shoemaker-2003 |
| HMMR | AURKA | 0.025534 | Co-localization | Johnson-Shoemaker-2003 |
| HMMR | BUB1B | 0.057764 | Co-localization | Johnson-Shoemaker-2003 |
| HMMR | CCNB1 | 0.016433 | Co-localization | Johnson-Shoemaker-2003 |
| HMMR | CDCA3 | 0.068973 | Co-localization | Johnson-Shoemaker-2003 |
| HMMR | CDK1 | 0.029004 | Co-localization | Johnson-Shoemaker-2003 |
| HMMR | CENPE | 0.069337 | Co-localization | Johnson-Shoemaker-2003 |
| HMMR | KIF11 | 0.036592 | Co-localization | Johnson-Shoemaker-2003 |
| HMMR | NDC80 | 0.067245 | Co-localization | Johnson-Shoemaker-2003 |
| HMMR | NEK2 | 0.060952 | Co-localization | Johnson-Shoemaker-2003 |
| HMMR | PRC1 | 0.043981 | Co-localization | Johnson-Shoemaker-2003 |
| HMMR | TOP2A | 0.047427 | Co-localization | Johnson-Shoemaker-2003 |
| KIF11 | AURKA | 0.034444 | Co-localization | Johnson-Shoemaker-2003 |
| KIF11 | GINS1 | 0.024208 | Co-localization | Johnson-Shoemaker-2003 |
| KIF11 | KIF23 | 0.05414 | Co-localization | Johnson-Shoemaker-2003 |
| KIF11 | NDC80 | 0.088941 | Co-localization | Johnson-Shoemaker-2003 |
| KIF11 | NEK2 | 0.069407 | Co-localization | Johnson-Shoemaker-2003 |
| KIF11 | NUSAP1 | 0.025559 | Co-localization | Johnson-Shoemaker-2003 |
| KIF11 | PRC1 | 0.055596 | Co-localization | Johnson-Shoemaker-2003 |
| KIF11 | TOP2A | 0.059744 | Co-localization | Johnson-Shoemaker-2003 |
| KIF23 | GINS1 | 0.023529 | Co-localization | Johnson-Shoemaker-2003 |
| KIF23 | PRC1 | 0.053692 | Co-localization | Johnson-Shoemaker-2003 |
| KIF4A | AURKA | 0.060505 | Co-localization | Johnson-Shoemaker-2003 |
| KIF4A | GINS1 | 0.038736 | Co-localization | Johnson-Shoemaker-2003 |
| KIF4A | HMMR | 0.052557 | Co-localization | Johnson-Shoemaker-2003 |
| KIF4A | KIF11 | 0.081168 | Co-localization | Johnson-Shoemaker-2003 |
| KIF4A | KIF23 | 0.095492 | Co-localization | Johnson-Shoemaker-2003 |
| KIF4A | PRC1 | 0.085972 | Co-localization | Johnson-Shoemaker-2003 |
| KIF4A | ZWINT | 0.036029 | Co-localization | Johnson-Shoemaker-2003 |
| MKI67 | CCNA2 | 0.048699 | Co-localization | Johnson-Shoemaker-2003 |
| MKI67 | GINS1 | 0.051945 | Co-localization | Johnson-Shoemaker-2003 |
| MKI67 | NUSAP1 | 0.060467 | Co-localization | Johnson-Shoemaker-2003 |
| NDC80 | CCNB1 | 0.040975 | Co-localization | Johnson-Shoemaker-2003 |
| NDC80 | NUSAP1 | 0.049272 | Co-localization | Johnson-Shoemaker-2003 |
| PRC1 | NEK2 | 0.090328 | Co-localization | Johnson-Shoemaker-2003 |
| PRC1 | NUSAP1 | 0.032548 | Co-localization | Johnson-Shoemaker-2003 |
| SMC4 | AURKA | 0.045651 | Co-localization | Johnson-Shoemaker-2003 |
| SMC4 | AURKB | 0.117103 | Co-localization | Johnson-Shoemaker-2003 |
| SMC4 | CCNA2 | 0.029105 | Co-localization | Johnson-Shoemaker-2003 |
| SMC4 | CCNB1 | 0.029716 | Co-localization | Johnson-Shoemaker-2003 |
| SMC4 | CDCA3 | 0.129006 | Co-localization | Johnson-Shoemaker-2003 |
| SMC4 | CENPF | 0.10669 | Co-localization | Johnson-Shoemaker-2003 |
| SMC4 | HMMR | 0.048885 | Co-localization | Johnson-Shoemaker-2003 |
| SMC4 | NDC80 | 0.125308 | Co-localization | Johnson-Shoemaker-2003 |
| SMC4 | PRC1 | 0.073996 | Co-localization | Johnson-Shoemaker-2003 |
| TOP2A | BUB1B | 0.108123 | Co-localization | Johnson-Shoemaker-2003 |
| TOP2A | CCNA2 | 0.029854 | Co-localization | Johnson-Shoemaker-2003 |
| TOP2A | CCNB1 | 0.027917 | Co-localization | Johnson-Shoemaker-2003 |
| TOP2A | CENPE | 0.123455 | Co-localization | Johnson-Shoemaker-2003 |
| TOP2A | CENPF | 0.108719 | Co-localization | Johnson-Shoemaker-2003 |
| TOP2A | MKI67 | 0.127915 | Co-localization | Johnson-Shoemaker-2003 |
| TOP2A | NDC80 | 0.125157 | Co-localization | Johnson-Shoemaker-2003 |
| TOP2A | NUSAP1 | 0.037851 | Co-localization | Johnson-Shoemaker-2003 |
| ZWINT | CCNA2 | 0.01308 | Co-localization | Johnson-Shoemaker-2003 |
| ZWINT | CCNF | 0.044382 | Co-localization | Johnson-Shoemaker-2003 |
| ZWINT | CDCA3 | 0.04763 | Co-localization | Johnson-Shoemaker-2003 |
| ZWINT | GINS1 | 0.015266 | Co-localization | Johnson-Shoemaker-2003 |
| ZWINT | KIF11 | 0.023685 | Co-localization | Johnson-Shoemaker-2003 |
| ZWINT | MKI67 | 0.053028 | Co-localization | Johnson-Shoemaker-2003 |
| ZWINT | NUSAP1 | 0.015703 | Co-localization | Johnson-Shoemaker-2003 |
| ZWINT | TOP2A | 0.032271 | Co-localization | Johnson-Shoemaker-2003 |
| CDC25C | KIF4A | 0.004424 | Genetic Interactions | Lin-Smith-2010 |
| CENPE | KIF23 | 0.001224 | Genetic Interactions | Lin-Smith-2010 |
| CENPF | NUSAP1 | 0.001656 | Genetic Interactions | Lin-Smith-2010 |
| DEPDC1 | CENPF | 5.16E-04 | Genetic Interactions | Lin-Smith-2010 |
| DEPDC1 | ZWINT | 4.36E-04 | Genetic Interactions | Lin-Smith-2010 |
| KIF23 | CENPF | 7.50E-04 | Genetic Interactions | Lin-Smith-2010 |
| MKI67 | BUB1B | 0.001066 | Genetic Interactions | Lin-Smith-2010 |
| PRC1 | NUSAP1 | 0.004099 | Genetic Interactions | Lin-Smith-2010 |
| SMC4 | HMMR | 0.003328 | Genetic Interactions | Lin-Smith-2010 |
| ZWINT | CCNA2 | 0.001035 | Genetic Interactions | Lin-Smith-2010 |
| ZWINT | CENPF | 3.80E-04 | Genetic Interactions | Lin-Smith-2010 |
| AURKB | BUB1B | 0.005437 | Pathway | REACTOME |
| AURKB | CENPE | 0.006394 | Pathway | REACTOME |
| AURKB | CENPF | 0.006394 | Pathway | REACTOME |
| AURKB | NDC80 | 0.006463 | Pathway | REACTOME |
| BUB1B | NEK2 | 0.004457 | Pathway | REACTOME |
| CDK1 | BUB1B | 0.003314 | Pathway | REACTOME |
| CDK1 | CCNA2 | 0.103504 | Pathway | REACTOME |
| CDK1 | CCNB1 | 0.007875 | Pathway | REACTOME |
| CDK1 | NEK2 | 0.003378 | Pathway | REACTOME |
| CENPE | BUB1B | 0.005142 | Pathway | REACTOME |
| CENPE | CENPF | 0.006047 | Pathway | REACTOME |
| CENPF | BUB1B | 0.005142 | Pathway | REACTOME |
| NDC80 | BUB1B | 0.005197 | Pathway | REACTOME |
| NDC80 | CENPE | 0.006112 | Pathway | REACTOME |
| NDC80 | CENPF | 0.006112 | Pathway | REACTOME |
| ZWINT | AURKB | 0.006394 | Pathway | REACTOME |
| ZWINT | BUB1B | 0.005142 | Pathway | REACTOME |
| ZWINT | CENPE | 0.006047 | Pathway | REACTOME |
| ZWINT | CENPF | 0.006047 | Pathway | REACTOME |
| ZWINT | NDC80 | 0.006112 | Pathway | REACTOME |
| AURKA | NDC80 | 0.053833 | Physical Interactions | BIOGRID-SMALL-SCALE-STUDIES |
| AURKB | BUB1B | 0.027351 | Physical Interactions | BIOGRID-SMALL-SCALE-STUDIES |
| AURKB | KIF20A | 0.08855 | Physical Interactions | BIOGRID-SMALL-SCALE-STUDIES |
| AURKB | NDC80 | 0.049858 | Physical Interactions | BIOGRID-SMALL-SCALE-STUDIES |
| BUB1B | NEK2 | 0.077907 | Physical Interactions | BIOGRID-SMALL-SCALE-STUDIES |
| CCNA2 | BUB1B | 0.038654 | Physical Interactions | BIOGRID-SMALL-SCALE-STUDIES |
| CCNA2 | PRC1 | 0.098866 | Physical Interactions | BIOGRID-SMALL-SCALE-STUDIES |
| CCNB1 | PRC1 | 0.066589 | Physical Interactions | BIOGRID-SMALL-SCALE-STUDIES |
| CCNF | CCNB1 | 0.05861 | Physical Interactions | BIOGRID-SMALL-SCALE-STUDIES |
| CCNF | NUSAP1 | 1.329665 | Physical Interactions | BIOGRID-SMALL-SCALE-STUDIES |
| CDC25C | CCNB1 | 0.036374 | Physical Interactions | BIOGRID-SMALL-SCALE-STUDIES |
| CDC25C | CDK1 | 0.025316 | Physical Interactions | BIOGRID-SMALL-SCALE-STUDIES |
| CDK1 | CCNA2 | 0.0161 | Physical Interactions | BIOGRID-SMALL-SCALE-STUDIES |
| CDK1 | CCNB1 | 0.010843 | Physical Interactions | BIOGRID-SMALL-SCALE-STUDIES |
| CDK1 | CCNF | 0.040792 | Physical Interactions | BIOGRID-SMALL-SCALE-STUDIES |
| CDK1 | KIF11 | 0.055631 | Physical Interactions | BIOGRID-SMALL-SCALE-STUDIES |
| CDK1 | MKI67 | 0.049914 | Physical Interactions | BIOGRID-SMALL-SCALE-STUDIES |
| CDK1 | PRC1 | 0.046345 | Physical Interactions | BIOGRID-SMALL-SCALE-STUDIES |
| CENPE | BUB1B | 0.110431 | Physical Interactions | BIOGRID-SMALL-SCALE-STUDIES |
| CENPE | PRC1 | 0.28245 | Physical Interactions | BIOGRID-SMALL-SCALE-STUDIES |
| HMMR | AURKA | 0.08814 | Physical Interactions | BIOGRID-SMALL-SCALE-STUDIES |
| KIF23 | PRC1 | 0.269571 | Physical Interactions | BIOGRID-SMALL-SCALE-STUDIES |
| KIF4A | PRC1 | 0.541663 | Physical Interactions | BIOGRID-SMALL-SCALE-STUDIES |
| NDC80 | NEK2 | 0.142019 | Physical Interactions | BIOGRID-SMALL-SCALE-STUDIES |
| ZWINT | NDC80 | 0.189211 | Physical Interactions | BIOGRID-SMALL-SCALE-STUDIES |
| ASPM | NDC80 | 0.014681 | Physical Interactions | IREF-BIOGRID |
| AURKA | NDC80 | 0.001274 | Physical Interactions | IREF-BIOGRID |
| AURKB | AURKA | 8.21E-04 | Physical Interactions | IREF-BIOGRID |
| AURKB | BUB1B | 0.001662 | Physical Interactions | IREF-BIOGRID |
| AURKB | KIF20A | 0.005867 | Physical Interactions | IREF-BIOGRID |
| AURKB | NDC80 | 0.001372 | Physical Interactions | IREF-BIOGRID |
| CCNA2 | PRC1 | 0.008869 | Physical Interactions | IREF-BIOGRID |
| CCNB1 | PRC1 | 0.007347 | Physical Interactions | IREF-BIOGRID |
| CCNF | CCNB1 | 0.011867 | Physical Interactions | IREF-BIOGRID |
| CCNF | NUSAP1 | 0.040369 | Physical Interactions | IREF-BIOGRID |
| CDC25C | CCNB1 | 0.005016 | Physical Interactions | IREF-BIOGRID |
| CDC25C | CDK1 | 0.002071 | Physical Interactions | IREF-BIOGRID |
| CDK1 | CCNA2 | 0.00116 | Physical Interactions | IREF-BIOGRID |
| CDK1 | CCNB1 | 9.61E-04 | Physical Interactions | IREF-BIOGRID |
| CDK1 | CCNF | 0.0049 | Physical Interactions | IREF-BIOGRID |
| CDK1 | KIF11 | 0.003103 | Physical Interactions | IREF-BIOGRID |
| CDK1 | MKI67 | 0.00175 | Physical Interactions | IREF-BIOGRID |
| CDK1 | PRC1 | 0.003034 | Physical Interactions | IREF-BIOGRID |
| CENPE | BUB1B | 0.00841 | Physical Interactions | IREF-BIOGRID |
| CENPE | PRC1 | 0.022913 | Physical Interactions | IREF-BIOGRID |
| HMMR | AURKA | 0.004422 | Physical Interactions | IREF-BIOGRID |
| HMMR | NDC80 | 0.007391 | Physical Interactions | IREF-BIOGRID |
| KIF23 | PRC1 | 0.015221 | Physical Interactions | IREF-BIOGRID |
| KIF4A | PRC1 | 0.031591 | Physical Interactions | IREF-BIOGRID |
| NDC80 | BUB1B | 0.00258 | Physical Interactions | IREF-BIOGRID |
| NDC80 | CCNB1 | 0.002226 | Physical Interactions | IREF-BIOGRID |
| NDC80 | NEK2 | 0.003632 | Physical Interactions | IREF-BIOGRID |
| ZWINT | BUB1B | 0.003317 | Physical Interactions | IREF-BIOGRID |
| ZWINT | NDC80 | 0.002739 | Physical Interactions | IREF-BIOGRID |
| AURKA | CCNA2 | 0.042096 | Predicted | Wu-Stein-2010 |
| AURKA | CCNB1 | 0.074055 | Predicted | Wu-Stein-2010 |
| AURKA | CCNF | 0.113663 | Predicted | Wu-Stein-2010 |
| CCNA2 | CCNB1 | 0.052715 | Predicted | Wu-Stein-2010 |
| CCNA2 | CCNF | 0.08091 | Predicted | Wu-Stein-2010 |
| CCNA2 | NEK2 | 0.0492 | Predicted | Wu-Stein-2010 |
| CCNB1 | NEK2 | 0.086552 | Predicted | Wu-Stein-2010 |
| CCNF | CCNB1 | 0.142336 | Predicted | Wu-Stein-2010 |
| CCNF | NEK2 | 0.132844 | Predicted | Wu-Stein-2010 |
| CDC25C | CCNA2 | 0.086134 | Predicted | Wu-Stein-2010 |
| CDK1 | AURKA | 0.016256 | Predicted | Wu-Stein-2010 |
| CDK1 | AURKB | 0.051019 | Predicted | Wu-Stein-2010 |
| CDK1 | CCNF | 0.031244 | Predicted | Wu-Stein-2010 |
| CDK1 | KIF11 | 0.024711 | Predicted | Wu-Stein-2010 |
| CDK1 | MKI67 | 0.104805 | Predicted | Wu-Stein-2010 |
| CDK1 | TOP2A | 0.01692 | Predicted | Wu-Stein-2010 |
| CENPE | KIF23 | 0.464382 | Predicted | Wu-Stein-2010 |
| KIF11 | AURKA | 0.089896 | Predicted | Wu-Stein-2010 |
| KIF11 | CENPE | 0.306163 | Predicted | Wu-Stein-2010 |
| KIF11 | KIF23 | 0.207276 | Predicted | Wu-Stein-2010 |
| NDC80 | NEK2 | 0.371184 | Predicted | Wu-Stein-2010 |
| ZWINT | NUSAP1 | 1.261725 | Predicted | Wu-Stein-2010 |
| AURKA | NEK2 | 0.003984 | Shared protein domains | INTERPRO |
| AURKB | AURKA | 0.003518 | Shared protein domains | INTERPRO |
| AURKB | NEK2 | 0.004014 | Shared protein domains | INTERPRO |
| BUB1B | NEK2 | 0.007875 | Shared protein domains | INTERPRO |
| CCNA2 | CCNB1 | 0.029667 | Shared protein domains | INTERPRO |
| CCNA2 | CCNF | 0.021717 | Shared protein domains | INTERPRO |
| CCNF | CCNB1 | 0.021717 | Shared protein domains | INTERPRO |
| CDK1 | AURKA | 0.003526 | Shared protein domains | INTERPRO |
| CDK1 | AURKB | 0.003552 | Shared protein domains | INTERPRO |
| CDK1 | NEK2 | 0.004023 | Shared protein domains | INTERPRO |
| CENPE | KIF20A | 0.020494 | Shared protein domains | INTERPRO |
| CENPE | KIF23 | 0.020211 | Shared protein domains | INTERPRO |
| KIF11 | CENPE | 0.020211 | Shared protein domains | INTERPRO |
| KIF11 | KIF20A | 0.020211 | Shared protein domains | INTERPRO |
| KIF11 | KIF23 | 0.019932 | Shared protein domains | INTERPRO |
| KIF23 | KIF20A | 0.020211 | Shared protein domains | INTERPRO |
| KIF4A | CENPE | 0.020181 | Shared protein domains | INTERPRO |
| KIF4A | KIF11 | 0.019902 | Shared protein domains | INTERPRO |
| KIF4A | KIF20A | 0.020181 | Shared protein domains | INTERPRO |
| KIF4A | KIF23 | 0.019902 | Shared protein domains | INTERPRO |
| AURKA | BUB1B | 1.73E-04 | Shared protein domains | PFAM |
| AURKA | NEK2 | 1.29E-04 | Shared protein domains | PFAM |
| AURKB | AURKA | 1.14E-04 | Shared protein domains | PFAM |
| AURKB | BUB1B | 1.53E-04 | Shared protein domains | PFAM |
| AURKB | NEK2 | 1.14E-04 | Shared protein domains | PFAM |
| BUB1B | NEK2 | 1.73E-04 | Shared protein domains | PFAM |
| CCNA2 | CCNB1 | 0.001582 | Shared protein domains | PFAM |
| CCNA2 | CCNF | 9.85E-04 | Shared protein domains | PFAM |
| CCNF | CCNB1 | 9.85E-04 | Shared protein domains | PFAM |
| CDK1 | AURKA | 1.29E-04 | Shared protein domains | PFAM |
| CDK1 | AURKB | 1.14E-04 | Shared protein domains | PFAM |
| CDK1 | BUB1B | 1.73E-04 | Shared protein domains | PFAM |
| CDK1 | NEK2 | 1.29E-04 | Shared protein domains | PFAM |
| CENPE | KIF20A | 0.001022 | Shared protein domains | PFAM |
| CENPE | KIF23 | 0.001022 | Shared protein domains | PFAM |
| KIF11 | CENPE | 0.001022 | Shared protein domains | PFAM |
| KIF11 | KIF20A | 0.001022 | Shared protein domains | PFAM |
| KIF11 | KIF23 | 0.001022 | Shared protein domains | PFAM |
| KIF23 | KIF20A | 0.001022 | Shared protein domains | PFAM |
| KIF4A | CENPE | 0.001021 | Shared protein domains | PFAM |
| KIF4A | KIF11 | 0.001021 | Shared protein domains | PFAM |
| KIF4A | KIF20A | 0.001021 | Shared protein domains | PFAM |
| KIF4A | KIF23 | 0.001021 | Shared protein domains | PFAM |
